# Nitrogen Sources and Iron Availability Affect Pigment Biosynthesis and Nutrient Consumption in *Anabaena* sp. UTEX 2576

**DOI:** 10.3390/microorganisms9020431

**Published:** 2021-02-19

**Authors:** Daniel A. Norena-Caro, Tara M. Malone, Michael G. Benton

**Affiliations:** Cain Department of Chemical Engineering, 3307 Patrick F. Taylor Hall, Louisiana State University and A&M College, Baton Rouge, LA 70803, USA; dnoren1@lsu.edu (D.A.N.-C.); tmalon5@lsu.edu (T.M.M.)

**Keywords:** cyanobacteria, β-carotene, phycobiliproteins, oxidative stress, metal homeostasis, mineral media

## Abstract

*Anabaena* sp. UTEX 2576 metabolizes multiple nitrogen (N) sources and is deemed a biotechnological platform for chemical production. Cyanobacteria have been identified as prolific producers of biofertilizers, biopolymers, biofuels, and other bioactive compounds. Here, we analyze the effect of different N-sources and Fe availability on the bioproduction of phycobiliproteins and β-carotene. We characterize nutrient demand in modified BG11 media, including data on CO_2_ fixation rates, N-source consumption, and mineral utilization (e.g., phosphorus (P), and 11 metallic elements). Results suggest that non-diazotrophic cultures grow up to 60% faster than diazotrophic cells, resulting in 20% higher CO_2_-fixation rates. While the production of β-carotene was maximum in medium with NaNO_3_, Fe starvation increased the cellular abundance of C-phycocyanin and allophycocyanin by at least 22%. Compared to cells metabolizing NaNO_3_ and N_2_, cultures adapted to urea media increased their P, calcium and manganese demands by at least 72%, 97% and 76%, respectively. Variations on pigmentation and nutrient uptake were attributed to changes in phycocyanobilin biosynthesis, light-induced oxidation of carotenoids, and urea-promoted peroxidation. This work presents insights into developing optimal *Anabaena* culture for efficient operations of bioproduction and wastewater bioremediation with cyanobacteria.

## 1. Introduction

Chemical production by cyanobacteria has gained interest for carbon dioxide (CO_2_) bio-sequestration applications. In this regard, most CO_2_ transformation studies have focused on the production of biofuels and commodities derived from the cyanobacterial central carbon metabolism [1]. Nevertheless, recent evidence indicates that feasible large-scale cyanobacterial biotechnology should not only focus on biofuel production, but also on synthesis of biofertilizers, biopolymers, pigments, antioxidants, vitamins, and secondary metabolites [2,3]. From this perspective, the biotechnological importance of *Anabaena* sp. UTEX 2576 (a.k.a., *Nostoc* sp. PCC 7120 and, henceforth, *Anabaena*) is enhanced when considering their ability to utilize different nitrogen sources (N-sources) to fuel photosynthetic biosynthesis.

Although CO_2_ fixation is the most attractive feature of cyanobacterial metabolism, their global metabolic network is also affected by the N-source and iron (Fe) concentration in the growth medium, especially for N_2_-fixing species like *Anabaena* [4,5,6,7]. While the N-source is used for synthesizing proteins, nucleic acids, co-factors, and secondary metabolites [8], Fe is essential for the synthesis of DNA and iron-sulfur proteins [9]. Given that iron-sulfur proteins are involved in photosynthesis and N assimilation, cyanobacterial cells demand at least 10 times more Fe than non-photosynthetic bacteria like *Escherichia coli* [10,11]. In addition, Fe requirements of diazotrophic species like *Anabaena* are even higher compared to non-N_2_-fixing cyanobacteria [12].

Fe is essential for bacterial metabolism and is also the most important transition metal added to the cyanobacterial growth media [13,14]. However, excessive illumination conditions trigger Fe-catalyzed formation of reactive oxygen species (ROS) like superoxide (O_2_^–^) and hydrogen peroxide (H_2_O_2_) during photosynthesis [10,15,16]. Therefore, Fe plays a contradictory role in cyanobacterial metabolism, both as a nutrient and catalyzer of damaging oxidative reactions. To deal with this paradox, cyanobacteria have evolved Fe homeostasis mechanisms mediated by ferric uptake regulator proteins (FUR) [9]. Although the FUR proteins are mainly responsible for maintaining the intracellular Fe balance in cyanobacterial cells, they are also crucial for keeping the balance of other essential metals like manganese (Mn), zinc (Zn), and nickel (Ni) [9,17,18,19]. The consumption of other important elements like phosphorus (P), calcium (Ca), magnesium (Mg), boron (B), molybdenum (Mo), copper (Cu), and cobalt (Co) is not under the direct influence of FUR proteins [4,5,9,20]. However, the question of how Fe availability affects the consumption of these nutrients remains under study.

Cyanobacteria can grow in mineral media without any source of organic carbon (C) because they are autotrophic organisms, capable of oxygenic photosynthesis. The light phase of photosynthesis is facilitated by light-sensitive pigment-metalloprotein complexes that catalyze electron transfer reactions to produce ATP and NADPH. In cyanobacteria, such pigment–protein complexes are photosystem II (PSII, EC 1.10.3.9), photosystem I (PSI, EC 1.97.1.12) and phycobilisomes (PBSs), which are abundant in the thylakoid membranes. In PSII, a Mn-Ca based cluster (Mn_4_CaO_5_) works in tandem with light-excited chlorophyll *a* P680, pheophytin *a*, plastoquinone (PQ-9) and β-carotene to draw electrons from water, producing molecular Oxygen (O_2_), O_2_^−^ and H_2_O_2_ [21,22,23]. Eventually, O_2_^−^ and H_2_O_2_ are degraded by the action of superoxide dismutase (SOD, EC 1.15.1.1), and Mn-catalase (EC 1.11.1.6) [10,24,25]. In PSI, light-excited chlorophyll *a* P700, phylloquinone (vitamin K_1_), β-carotene, and ferredoxins (iron-sulphur proteins), work together to transfer electrons from plastocyanin to NADP^+^, producing NADPH [26]. In a parallel transport reaction, the H^+^ gradient generated across the thylakoid membranes fuels the production of ATP by ATP-synthase [27]. Cyanobacteria have developed PBSs to take advantage of a wider portion of the visible light spectrum for photosynthesis. PBSs are light-harvesting protein complexes, responsible for the cyanobacterial chromatic acclimation mechanism [28]. In *Anabaena*, these complexes are composed of multiple disc-shaped stacked subunits of phycobiliproteins (PBPs), forming large antenna-like structures of five inner cylinders and eight peripheral rods connected to the photosystems [29,30]. The distinctive PBP subunits of the *Anabaena* genus are C-phycocyanin (CPC, ~35.8 kDa), phycoerythrocyanin (PEC, ~35.7 kDa) and allophycocyanin (APC, ~34.7 kDa) [31].

Although the main photosynthetic pigment in cyanobacteria is chlorophyll *a* (Chl*a*), a modified Mg-containing a chlorin ring with a side phytol chain, other auxiliary photosynthetic pigments are carotenoids and phycobilins [27,32]. The carotenoids are a group of isoprenoid compounds formed by different types of carotene (e.g., α-carotene, β-carotene, γ-carotene, lycopene, torulene) and their oxygenated derivatives, the xanthophylls (e.g., echinenone, myxoxanthophyll, canthaxanthin, zeaxanthin) [33,34,35]. These compounds play multiple roles in photosynthetic organisms, participating in light-harvesting and defense mechanisms like energy dissipation under excess illumination, non-photochemical quenching, and photo-oxidation and lipid peroxidation protection [36,37]. While β-carotene is the most common carotenoid in the photosystems of *Anabaena* and other cyanobacteria [23,26,33,34,35,38], echinenone, hydroxyechinenone, canthaxanthin, and zeaxanthin can be produced as a result of non-photochemical quenching. This occurs when β-carotene ketolase (EC 1.14.99.63) oxidizes β-carotene to counteract the damaging effect of ROS during oxidative stress, Fe surplus and deficit, and growth on urea [33,37,39,40,41]. The phycobilins are the chromophore molecules linked to the PBPs in cyanobacterial phycobilisomes. Chemically, these pigments are open-chain tertrapyrroles biosynthesized from heme and biliverdin [42,43]. Therefore, the synthesis of phycobilins is directly related with Chl*a* production and Fe metabolism [12]. In *Anabaena*, the most important phycobilin pigments are phycocyanobilin (PCB) and phycoviolobilin (PVB), which are present in CPC, APC and PEC proteins. It has been demonstrated that Fe limitation leads to increased expression of *furA* gene, which encodes a master transcriptional regulator of Fe metabolism that also affects pigment biosynthesis [12,44].

Considering all the previous relationships, quantifying the impact of different N-sources and the Fe availability on the autotrophic metabolism of *Anabaena* is essential. Specifically, it is important to determine the impact of these nutrients on the growth kinetics and the bioproduction of valuable products of biotechnological interest like CPC, APC, PEC and β-carotene (pro-vitamin A), which are used in nutraceutical and cosmetic products as natural colorants, dietary supplements and anti-oxidant ingredients [45,46,47]. In addition, having a clearer understanding of the intricate relationship between N and Fe metabolism in photosynthetic microorganisms can provide useful insights to optimize culture media for large-scale operations with cyanobacteria [3,48,49]. The aim of this study is to quantify the effect of three different N-sources, i.e., dinitrogen (N_2_), nitrate (NO_3_) and urea (CH_4_N_2_O), and Fe availability on the production of β-carotene, Chl*a* and PBPs, by *Anabaena*. Moreover, this work also discusses nutrient consumption kinetics and presents a novel approach for efficient quantification of P and metallic elements in BG11 mineral medium. This study provides a systemic analysis on the cultivation of *Anabaena*, taking into consideration the balance between nutrient demand and oxidative stress.

## 2. Materials and Methods

Additional methodology details are presented in the Supplementary Materials.

### 2.1. Pre-Culture Conditions

*Anabaena* sp. strain UTEX 2576 was consistently kept as an axenic 250 mL culture for more than 3 months in BG11(N^−^) medium to preserve heterocyst differentiation. Axenic cultures were grown autotrophically (75 ± 7 µmol m^−2^ s^−1^) at 28 °C in an illuminated New Brunswick Innova 4340 incubator shaker (Edison, NJ, USA) at 130 rpm, with atmospheric CO_2_ concentration ~410 ppm. Continuous shaking was necessary to promote air-exchange and atmospheric CO_2_ dissolution. New axenic cultures were prepared every week from the original culture. The formulation of BG11(N^−^) is similar to standard BG11 medium, but it does not contain NaNO_3_ (See Supplementary Materials). The presence of heterocysts was periodically verified with optical and scanning electron microscopy (SEM). Because of their additional carbohydrate layer, heterocysts are larger than vegetative cells and their surface is more irregular [30]. A SEM picture of pre-cultured *Anabaena* filaments is presented in Figure S1. The conservation of N_2_-fixation ability was also easily verified because diazotrophic *Anabaena* are clumpier and their green coloration is darker than non-diazotrophic cultures (See Figure S5). Pre-cultures in BG11(N^−^) were used to prepare *Anabaena* pre-cultures with different N-sources. Pre-cultures in alternative media with NaNO_3_ and urea grew for at least one month before they were used to aseptically prepare the experimental cultures.

### 2.2. Culture Conditions

*Anabaena* was cultivated autotrophically at 28 °C in an illuminated New Brunswick Innova 4340 incubator shaker at 130 rpm, with atmospheric CO_2_ (410 ppm) and sodium carbonate (0.19 mM) as C-sources. Constant photosynthetic photon flux density (PPFD) of 75 ± 7 µmol m^−2^ s^−1^ was provided with four 20 W white fluorescent light bulbs. Cultures were maintained in 500 mL glass Erlenmeyer flasks covered with foam plugs. The culture volume in each flask was 150 mL. Growth media were variants of standard BG11 medium [13,14]. For convenience, media were named after the main N-source (e.g., BG11_N2_, BG11_NO3_, and BG11_urea_) in each culture type. Fresh BG11_NO3_ contained NaNO_3_ (17.7 mM), BG11_urea_ contained urea (3.0 mM) and BG11_N2_ lacked any significant N-source to preserve heterocyst formation inherited from diazotrophic pre-cultures [8,30]. Soluble Fe^3+^ was supplied in the form of Ammonium ferric citrate (Fe^3+^ as C_6_H_8_FeNO_7_), with starting Fe levels of 0.3 mg/L (5.4 × 10^−3^ mM), 1.2 mg/L (0.02 mM) and 5.0 mg/L (0.09 mM). Since the ferric substrate also contains ammonium, the Fe source adds a negligible amount of elemental N to the growth medium. Three independent biological replicates were prepared for each medium type and Fe level combination, for a total of 27 independent cultures. Additional details on growth media properties are presented in Tables S3 and S4. Considering the composition of each culture medium, the salinity varied from 0.3 g/L (BG11_N2_ and BG11_urea_) to 1.8 g/L (BG11_NO3_).

### 2.3. Measurement of Cellular Growth

Cell reproduction was monitored by tracking the change on apparent absorbance at 730 nm (Abs_730_) every other day for two weeks. Abs_730_ was routinely measured with a Beckman Coulter DU730 Life science ultraviolet (UV)-visible spectrophotometer. Given the filamentous nature of *Anabaena*, short 10-W sonication pulses (2 to 5 s) were used to homogenize 1–1.5 mL culture samples prior to any OD_730_ measurement. A Fischer Scientific sonic dismembrator model 500 (Pittsburgh, PA, USA) was used to sonicate the liquid samples. Biomass generation was monitored using Abs_730_ measurements, cell densities, and dry biomass readings. Recorded Abs_730_ values were converted to cell density and biomass concentration using correlation equations for diazotrophic (BG11_N2_) and non-diazotrophic (BG11_NO3_ and BG11_urea_) cultures (see Figure S2). Flow cytometry readings were performed in a BD Accuri C6flow cytometer (Ann Arbor, MI, USA), using a sample preparation protocol for filamentous cyanobacteria [50]. Biomass concentration (as dry cell weight of 10 mL of culture) was measured through filtration on Whatman 0.22 µm cellulose nitrate membranes and vacuum drying at 90 °C for 12 h. The Abs_730_ at the beginning of each growth experiment was standardized at 0.1 to ensure starting cellular populations (*N*_o_) between 6 × 10^5^ and 8 × 10^5^ cells mL ^−1^. Cellular growth rates and generation times were determined after fitting Abs_730_ data to a saturation kinetic model [51].

### 2.4. Extraction and Quantification of Phycobiliproteins (PBPs)

PBPs were extracted from the cells by digesting with lysozyme. Briefly, a 1.5 mL-sample of cyanobacterial culture was centrifuged for 15 min at 15,000 RCF. The resulting pellet was resuspended in 1 mL of lysozyme solution (2.7 mg/mL in TE buffer at pH 8.0) and sonicated for 10 s at 40 W with a Fischer Scientific sonic dismembrator model 500. The cyanobacterial pellet was digested at 37 °C for 8 h in a dry block incubator. During the digestion reaction, PBPs were released from the cells forming a blue extract. Lysed cells were separated from the extract after a second centrifugation step at 15,000 RCF for 15 min. The concentration of PBPs in the blue extract was estimated from spectrophotometric readings at 570 nm for PEC, 620 nm for CPC, and 650 nm for APC using specific equations for *Anabaena azollae* [52]. Total concentration of PBPs was calculated as the sum of PEC, CPC, and APC concentrations. Concentrations of PBPs were recorded every other day.

### 2.5. Extraction and Quantification of Chlorophyll a (Chla), Total Carotenoids (CaroT) and β-Carotene

Chlorophyll *a* (Chl*a*) and total carotenoids (CaroT) concentrations were determined every other day after solvent extraction with cold acetone. The concentration of Chl*a* was calculated from the absorbance of the extract at 664 nm using Beer’s law and an extinction coefficient of 87.67 L g^−1^ cm^−1^ [53].The same extract was analyzed to determine the concentration of CaroT (xanthophylls and carotenes) by measuring the absorbance at 470 nm and using an extinction coefficient of 250 L g^−1^ cm^−1^ [54,55].

The concentration of β-carotene and the ion abundances of other photosynthetic pigments (e.g., echinenone, pheophytin *a*, and chlorophyll *a*) were determined after extracting with cold methanol. These extracts were immediately analyzed after preparation with an Agilent 6230 Electrospray ionization Time-of-flight mass spectrometry (ESI-TOF MS) analyzer (Santa Clara, CA, USA). A 0.2 μL- aliquot of methanol extract was injected to the MS analyzer and combined with a continuous stream (0.4 mL/min) of 70% *v*/*v* acetonitrile and 30% *v*/*v* aqueous solution (0.1% *v*/*v* formic acid in water) at 30 °C. Mass acquisition in positive mode covered a range from 100 to 3200 m/z, keeping a fragmentor voltage of 150 V. The ion abundance of β-carotene was measured following the signal of the molecular ion ([C_40_H_56_]^+^, m/z = 536.4382) [46]. A calibration curve relating β-carotene concentration in methanol extracts with the abundance of the molecular ion is presented in Figure S16. Ion abundance for echinenone was determined by observing the peak of the protonated molecule ([C_40_H_54_O + H]^+^, m/z = 551.4253). Pheophytin *a* and Chl*a* ion abundances were related to the signals of their protonated molecules: [C_55_H_74_N_4_O_5_ + H]^+^, m/z = 871.5737 for pheophytin *a* and [C_55_H_74_MgN_4_O_5_ + H]^+^, m/z = 893.5431 for Chl*a*. MS signals for pure methanol were subtracted from MS data recorded for methanolic extracts. See supporting information for additional details about solvent extraction procedures.

### 2.6. Analysis of Mineral Elements with Inductively Coupled Plasma Optical Emission Spectroscopy (ICP-OES)

Inductively coupled plasma optical emission spectroscopy (ICP-OES) was used for growth medium quality control and to track changes in the concentration of mineral elements in liquid media during cellular growth. A multi-element ICP-OES detection method based on the U.S. Environmental Protection Agency (EPA) 200.7 protocol was developed for efficient quantification of B, Na, Mg, P, K, Ca, Mn, Fe, Co, Ni, Cu, Zn in fresh media and supernatants [56]. The minimum sample volume required for this analysis was 4 mL. Optical emission was measured with a PerkinElmer Optima 8000 ICP-OES spectrometer (Waltham, MA, USA). Radio frequency power was 1500 W and plasma viewing was set to axial mode. Plasma, auxiliary gas and nebulizer gas flow rates were set to 8 L/min, 0.2 L/min and 0.7 L/min, respectively. Samples were analyzed at a flow rate of 1 mL/min, using HNO_3_ 5% *v*/*v* as washing fluid. Detection wavelengths were iterated to minimize spectral interference. The calibration standards were selected considering the composition of standard mineral BG11 medium [13]. Initial measurements conducted on liquid samples digested with HNO_3_ 2% *v*/*v* at 85 °C demonstrated that acid digestion of cyanobacterial growth medium was not necessary. Calibration curves and detection wavelengths for each element are summarized in Figure S18. Concentrations of calibration standards are presented in Table S9.

### 2.7. Measurement of Total Organic Carbon (TOC), N-Source Concentration and Urease Activity

Total organic carbon (TOC) was measured using a commercial HACH high range total organic carbon reagent set (product #2760445). The concentrations of sodium nitrate and urea were measured for cell-free supernatants and growth media to determine consumption profiles of these nutrients in BG11_NO3_ and BG11_urea_ media. The consumption rate of N_2_ in BG11_N2_ (i.e., N2-fixation rate of diazotrophic *Anabaena*) was determined by measuring the total nitrogen (N_T_) content of cultures over time. Nitrate in BG11_NO3_, urea in BG11_urea_, and N_T_ in BG11_N2_ cultures were determined every other day from day zero to day 14, for a total of eight sampling points per biological replicate. The urease activity was measured with a BioVision Urease activity kit (Milpitas, CA, USA) [57], after sample homogenization with sonic dismembration at 40 W (three cycles of 30 s on ice), following the manufacturer’s instructions. Urease concentration in the lysed sample was determined with a Pierce BCA kit (Rockford, IL, USA) and results were measured with a BioTek Epoch 2 microplate spectrophotometer (Winooski, VT, USA). Detailed explanation of these methods is provided in the supporting information.

## 3. Results

### 3.1. Cellular Growth in Modified BG11 Media

**Figure 1:**
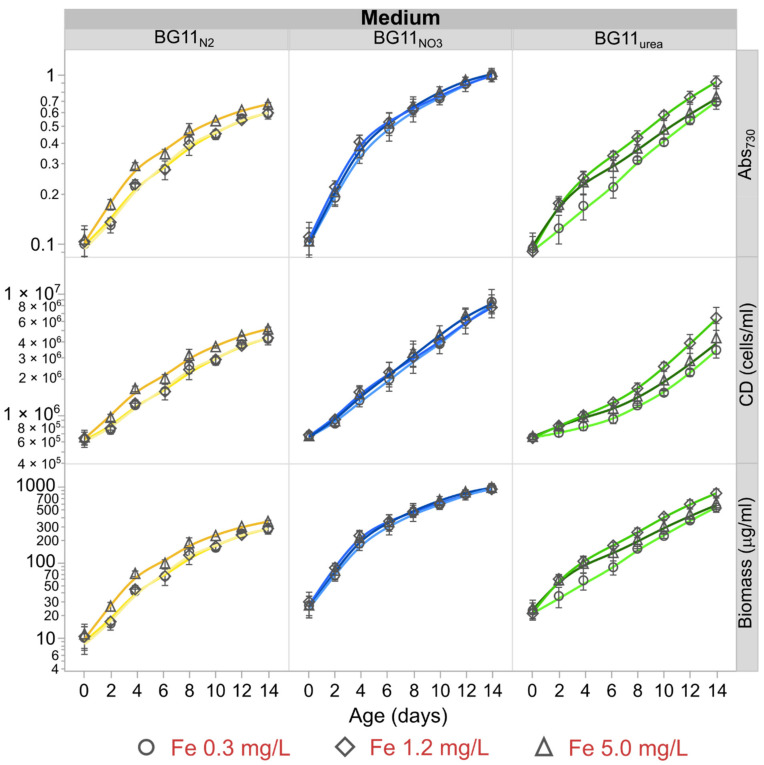
Growth kinetics of *Anabaena* in BG11_N2_, BG11_NO3_ and BG11_urea._ Fe concentration did not significantly affect cellular growth in BG11_NO3_, but higher biomass generation in BG11_urea_ cultures was observed with initial Fe supply of 1.2 mg/L. High Fe availability (5.0 mg/L) also favored growth and biomass generation in diazotrophic cultures (BG11_N2_). Overall, cell growth was lowest in BG11_N2_ medium, while BG11_urea_ cultures exhibited a longer exponential phase. Slowest growth was observed in BG11_urea_ with low Fe (0.3 mg/L). Highest biomass generation was observed in BG11 cultures. Absorbance at 730 nm was used as indicator of cellular density (second row) and biomass generation over time (third row). Starting nominal OD_730_ (at day 0) was 0.1 for all cultures. Markers represent average values of three biological replicates and error bars are constructed using one standard error from the mean. Optical densities were measured in triplicate.

Figure 1 summarizes the 14-days evolution of Abs_730_, cell density and biomass concentration for *Anabaena* cultured in BG11_N2_, BG11_NO3_, and BG11_urea_ media with different starting Fe levels. The results of fitting growth data to a saturation kinetic model are presented in Figure S3.

### 3.2. Effect of N-Source and Fe Levels on Growth Parameters

The N-source had the most significant effect on the growth rates, generation times and biomass production over 14 days as presented in Table 1 and Figure S4. The chemical properties of fresh media are presented in Tables S3 and S4. On average, growth rates in BG11_NO3_ medium were at least 60 ± 6% higher than in BG11_N2_ and BG11_urea_ media (*N* = 9 biological replicates per medium type). Remarkably, the average biomass generation of BG11_NO3_ cultures was more than two times the biomass generation of BG11_N2_ cultures over two weeks. Although Fe concentration did not significantly impact the growth kinetics in BG11_NO3_ media, Fe starvation (0.3 mg/L) significantly reduced the growth rate of cultures in BG11_urea_ by up to 44 ± 6%. For BG11_N2_ cultures, high Fe availability (5.0 mg/L) increased the growth rate by 28 ± 1%. Reduction in growth rates also meant lower biomass generation over 14 days. While cell cultures in BG11_N2_ and BG11_urea_ media exhibited comparable growth rates, the biomass generation was lowest for diazotrophic filaments in BG11_N2_. Cultures in BG11_NO3_ and BG11_urea_ also exhibited brighter green color than BG11_N2_ cultures (See Figure S5). Growth parameters are summarized in Table 1.

### 3.3. Effect of N-Source and Fe Levels on Accumulation of PBPs

**Figure 2:**
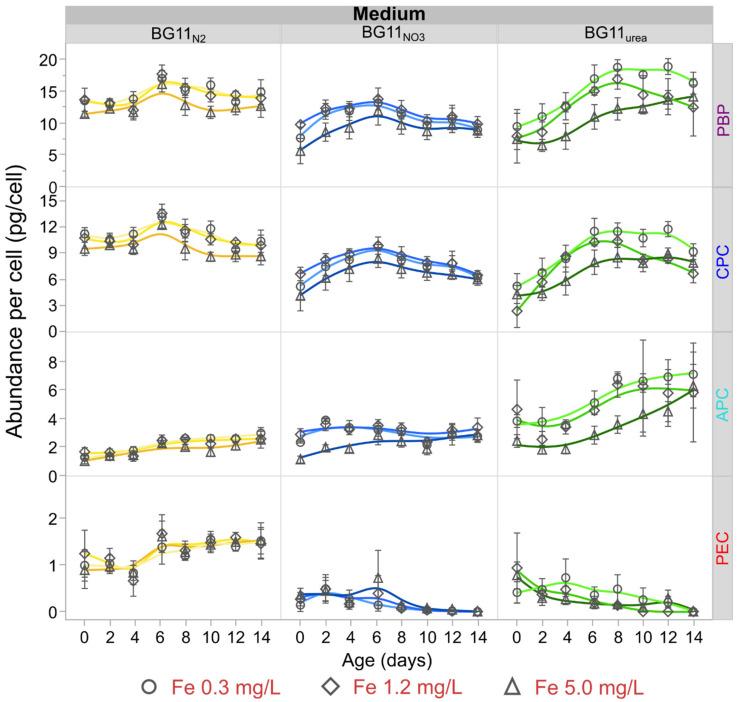
Abundance of phycobiliproteins (PBPs) per cell over time. Cells in BG11_N2_ and BG11_urea_ media presented higher abundances per cell of PBPs, compared to the cultures in BG11_NO3_. High Fe levels (5.0 mg/L) led to a statistically significant reduction on the abundance of C-phycocyanin (CPC) and allophycocyanin (APC) accumulation in BG11_NO3_ and BG11_urea_ media. The most pronounced reduction in the APC content per cell with increasing Fe was observed for BG11_urea_ cultures. Phycoerythrocyanin (PEC) abundance was higher for diazotrophic BG11_N2_ cultures. The PEC content in BG11_NO3_ and BG11_urea_ was consistently reduced over time, as non-diazotrophic filaments of *Anabaena* lowered the production of PEC. The highest abundance of APC was registered for low Fe cultures in BG11_urea_, specifically at the end of the experiments. Markers represent average values of three independent replicates and error bars are constructed using one standard error from the mean.

The concentration of CPC, APC, PEC, and total PBPs of each culture was determined and compared with the cell density to determine average abundance of these pigment–protein complexes per cell over time. Direct measurements of the concentration of PBPs for each medium type and Fe level treatment are summarized in Figure S6. Furthermore, Figure 2 presents the cellular abundance profiles of CPC, APC, PEC, and total PBPs over time. Remarkably, the cellular abundances of all PBPs were higher for BG11_N2_ and BG11_urea_ cultures, especially at low Fe levels. Using day 8 as reference, cells grown in BG11_N2_ accumulated 31 ± 3% more PBPs than BG11_NO3_ cells, and cells grown in BG11_urea_ accumulated 44 ± 6% more PBPs than BG11_NO3_ cells (See Table S5). In each growth medium, low Fe-levels had a positive effect on the average accumulation of CPC and APC (Figure 2). Considering data from Table S5, low Fe levels (0.3 mg/L) increased the CPC accumulation by 22 ± 7%, 24 ± 9%, and 31 ± 15% in BG11_N2_, BG11_NO3_, and BG11_urea_ cultures, respectively. Similarly, low Fe-levels (0.3 mg/L) increased the cellular abundance of APC by 31 ± 14%, 43 ± 15%, and 90 ± 30% in BG11_N2_, BG11_NO3_, and BG11_urea_ cultures, respectively. Accumulation of PEC was not significantly impacted by the Fe-level in BG11_N2_, nor BG11_NO3_ media. However, low Fe-levels (0.3 mg/L) were correlated with higher abundance of PEC in BG11_urea_ cultures from day 4. Figure 2 suggests that APC accumulation is favored in BG11_urea_ cultures and PEC accumulation is favored in BG11_N2_ cultures.

### 3.4. Effect of N-Source and Fe Levels on Accumulation of Chla and Carotenoids

**Figure 3:**
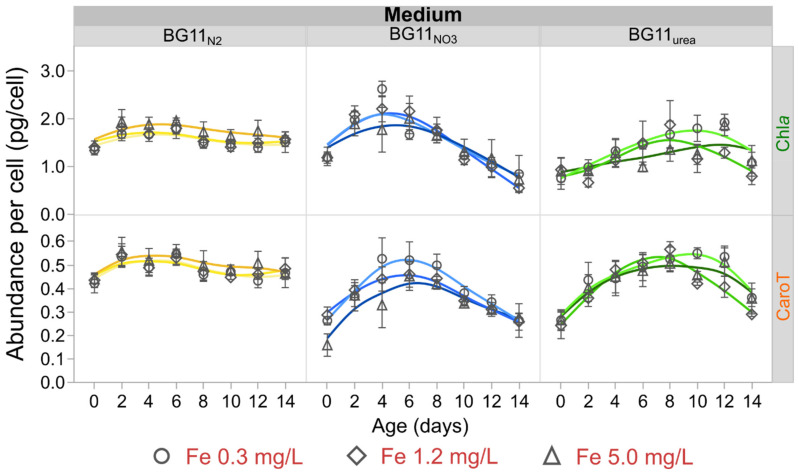
Relative abundance of Chlorophyll *a* (Chl*a*) and total carotenoids (CaroT) per cell. Chl*a* and CaroT profiles were constant in diazotrophic cultures, showing steady production of hydrophobic pigments until the end of the experiment. Non-diazotrophic cultures in BG11_NO3_ and BG11_urea_ media presented maximum values of Chl*a* and CaroT abundance per cell during their exponential phase. Markers represent average values of three independent replicates and error bars are constructed using one standard error from the mean.

Chl*a* and CaroT concentration profiles constructed with cold acetone extraction data are presented in Figure S7. After considering the cell density, relative cellular abundance data for Chl*a* and CaroT are summarized in Figure 3. Cultures in BG11_NO3_ and BG11_urea_ media exhibited similar profiles, with maximum values of Chl*a* and CaroT abundance in the exponential phase (between 4 and 8 days). In contrast, cellular abundances of Chl*a* and CaroT in diazotrophic cells (BG11_N2_) were almost constant. The production rate of Chl*a* and CaroT in BG11_N2_ was also constant (Figure S7). Although low Fe-levels (0.3 mg/L) apparently promoted higher abundance of Chl*a* and CaroT in BG11_NO3_ and BG11_urea_ cultures, statistical analysis did not provide definitive evidence to justify a significant effect of Fe-availability on cellular abundance of these hydrophobic pigments (Figure 3 and Table S6). However, the N-source of the growth medium significantly affected the cellular abundance of Chl*a*. Using the average values of Chl*a* abundance on day 4 (Table S6), cultures in BG11_urea_ accumulated around 50% less Chl*a* than cells grown in BG11_NO3_ and BG11_N2_ media.

Cold methanol extraction was performed for accurate quantification of β-carotene using TOF-MS (See Section 2.5). The ion abundance intensity of the β-carotene peak (molecular ion, [C_40_H_56_]^+^, m/z = 536.4382) was compared with the peak intensities of echinenone (protonated molecule, [C_40_H_54_O + H]^+^, m/z = 551.4253), pheophytin *a* (protonated molecule, [C_55_H_74_N_4_O_5_ + H]^+^, m/z = 871.5737) and Chl*a* (protonated molecule, [C_55_H_74_MgN_4_O_5_ + H]^+^, m/z = 893.5431). The β-carotene abundance per cell and the ratios of relative abundance (as peak intensity ratios) were used to analyze the oxidation of carotenoids in *Anabaena*. Methanol extraction was performed only for samples on days 6, 10 and 14. A statistical analysis of the data indicated that Fe levels did not have a significant impact on the β-carotene abundance per cell, at least for BG11_urea_ and BG11_NO3_ cultures (Table S6). Therefore, the abundance and oxidation of β-carotene were analyzed as a function of time and medium type. TOF-MS spectra from Figure S8 correspond to Methanol extractions on day 10, where the Chl*a* signals for cultures in all media were similar.

**Figure 4:**
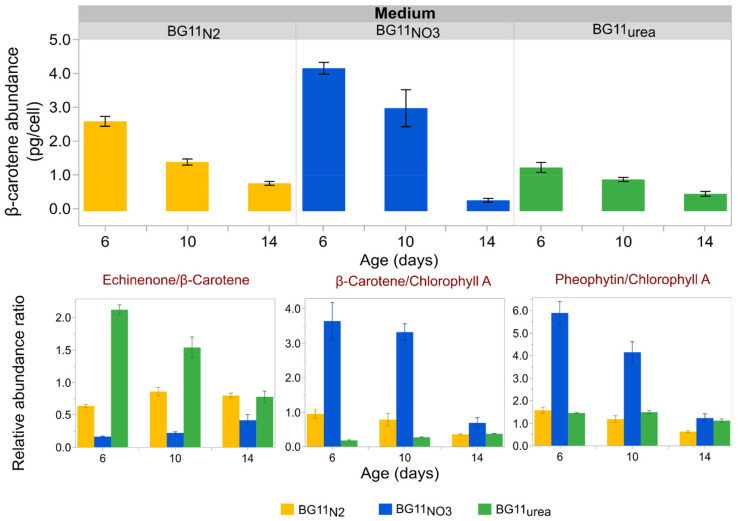
Cellular abundance and oxidation of β-carotene. Cellular abundance of β-carotene is reduced over time for BG11_N2_ and BG11_NO3_ cultures and is lowest for cells grown in BG11_urea_ medium (except for day 14). The echinenone to β-carotene ratio is used as an indicator of β-carotene oxidation, suggesting that cultures in BG11_urea_ are exposed to higher carotene-oxidation levels. The β-carotene to Chl*a* ratio indicates the abundance of non-oxidized carotenoid relative to the abundance of the main photosynthetic pigment. The pheophytin *a* to Chl*a* ratio is used as an indicator of photosystem II (PSII) integrity. Bars represent the mean of three measurements per sample and each error bar is constructed using one standard error from the mean. Data represent results for six biological replicates per medium type.

Figure 4 summarizes the cellular abundance of β-carotene in different growth media and compares the echinenone to β-carotene ratio using peak intensities. Cellular content of β-carotene was higher in younger cultures grown in BG11_N2_, BG11_NO3_, and BG11_urea_ media. Cells grown in BG11_urea_ exhibited relatively low abundance of β-carotene during the entire duration of the growth experiments. However, the lowest cellular abundance of β-carotene was observed for old BG11_NO3_ cultures (day 14). It was observed that younger cells grown in BG11_NO3_ medium presented the lowest echinenone to β-carotene ratios (days 6 and 10). The echinenone to β-carotene ratios were significantly higher in cells grown in BG11_N2_ and BG11_urea_ media. Figure 4 also presents ratios of relative abundance of β-carotene to Chl*a* and pheophytin *a* to Chl*a*. The β-carotene to Chl*a* ratios were highest for cells in BG11_NO3_ medium, specially at the beginning of the growth experiments. Cells grown in BG11_urea_ medium presented the lowest β-carotene to Chl*a* ratios. The pheophytin *a* to Chl*a* ratio was highest for younger cultures in BG11_NO3_ and lowest for older cells in BG11_N2_. The ratio of pheophytin *a* to Chl*a* was similar for cultures in BG11_N2_ and BG11_urea_, but significantly higher for younger *Anabaena* cells cultured in BG11_NO3_.

### 3.5. Consumption Rates of CO_2_ and N-Sources

Figure S9 presents the consumption profiles of CO_2_ and N-sources in BG11_N2_, BG11_NO3_, and BG11_urea_ cultures. These results are not presented for different Fe levels because changes in the initial Fe availability did not affect N or C demands significantly. CO_2_-fixation rates in BG11_N2_, BG11_NO3_, and BG11_urea_ were estimated after fitting the TOC formation data to a zero-order kinetic model. Given that *Anabaena* cells utilize inorganic carbon sources through the carbon concentrating mechanism (CCM), change in TOC is a direct result of atmospheric CO_2_-hydroxilation or consumption of Na_2_CO_3_ supplied by the growth medium [3]. CO_2_ consumption rates are directly obtained from the slopes of the straight lines depicted in Figure S9A. Considering molecular weights of C and CO_2_ (12 g/mol and 44 g/mol), a conversion factor of 3.67 was used to convert TOC formation rates to CO_2_ fixation rates. Consumption data of N-sources were fitted to an apparent first-order reaction model. The first-order rate constant, *k*, can be obtained from the slope of the straight lines presented in Figure S9B. NO_3_ and urea consumption rate constants equal the absolute value of the corresponding slope. Since fresh BG11_N2_ media contained negligible quantities of elemental N (Table S3), the N_2_ fixation rate can be directly obtained from the N_T_ slope describing the N-source consumption in BG11_N2_. Table 2 summarizes CO_2_ fixation and N-source kinetic parameters for *Anabaena* cultures. From Table 2, it can be concluded that cells grown in BG11_NO3_ and BG11_urea_ media (i.e., non-diazotrophic cultures) presented CO_2_ consumption rates 20 ± 5% higher than diazotrophic cultures in BG11_N2_. Given that urea degradation in the aqueous medium produces NH_4_^+^ and HCO_3_^−^, higher levels of CO_2_ consumption in BG11_urea_ cultures can also be associated with high urease (EC 3.5.1.5) activity levels in BG11_urea_ medium (see Figure S10). Higher CO_2_ consumption rates are also associated with faster consumption of the N-source during the exponential phase for non-diazotrophic cultures. On day 4, the consumption of elemental N in BG11_NO3_ was ~6.5 times the N-consumption in BG11_N2_. Likewise, the consumption of elemental N in BG11_urea_ was ~3.5 times the N consumption in BG11_N2_.

### 3.6. Effect of N-Source and Fe Levels on P Consumption and Micronutrient Utilization

ICP-OES was used to monitor changes in the concentration of mineral elements in liquid media during cellular growth. For most elements, concentration profiles were converted into consumption profiles after subtracting the element concentration at a given time from their initial concentration in fresh media. This was important for analyzing excess levels of mineral nutrients in the growth media. Figure S11 presents Fe consumption profiles in BG11_N2_, BG11_NO3_ and BG11_urea_. Regardless of the initial Fe concentration in the growth media, this element was almost immediately consumed at the beginning of the growth experiments. Figures S12 and S13 present consumption profiles of P, Ca, Mg, Mn, B, Mo, Zn, and Cu, suggesting that all these elements were supplied in excess. However, the amount of P supplied in the growth media was close to exhaustion after 14 days, especially for cultures in BG11_N2_ and BG11_NO3_ media with high Fe levels at the start. The consumption of Mo in BG11_N2_ cultures was also close to exhaustion after 14 days, especially under low and high starting Fe levels. This was expected because Mo is an essential component of nitrogenase (EC 1.18.6.1) [4,8]. Figure S14 presents concentration profiles of Na, K, Ni, and Co. These profiles were not converted to consumption data because they did not present significant variations over time in the growth medium. Moreover, the Co level in the growth media was so low that it approached the lower detection limit of the method. These consumption profiles suggest that it is possible to adjust the concentrations of micronutrients and minimize excess of these ingredients depending on the duration of each growth experiment. This is important for large-scale continuous operations, where growth medium formulation needs to be optimized.

**Figure 5:**
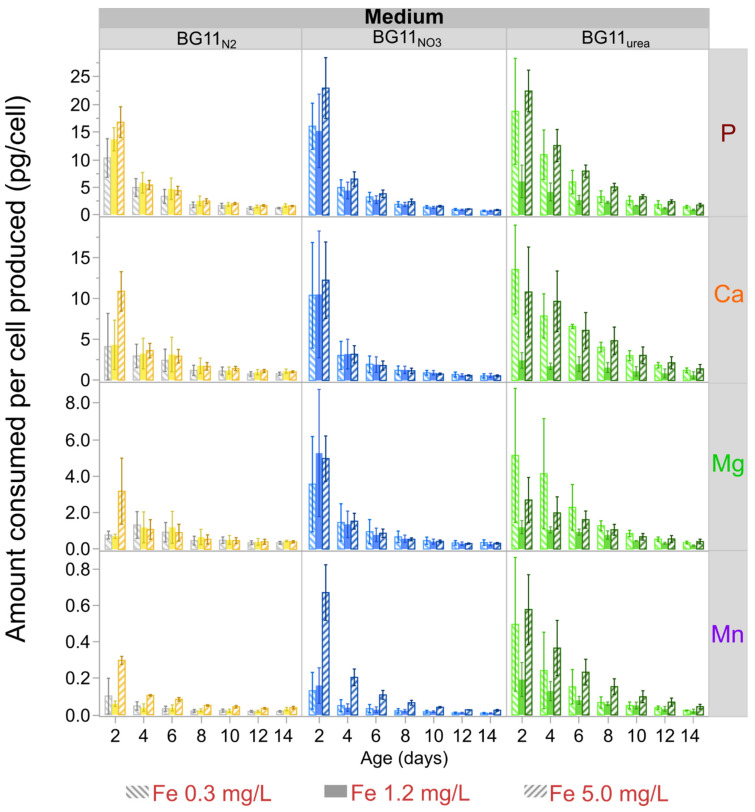
Consumption profiles of P, Ca, Mg and Mn per cell. The demand of these nutrient elements per cell is pronounced during the early stages of cellular growth. High initial Fe levels maximize the demand of P and Ca in all growth media. Low initial Fe levels increased Mg and Mn consumption in BG11_urea_ medium. High starting Fe levels also maximized the consumption of Mn in BG11_N2_ and BG11_NO3_ media, probably as a defense strategy against Fe promoted oxidation. Markers represent average values of three independent replicates and error bars are constructed using one standard error from the mean.

After considering the number of cells produced at a given time, the consumption profiles of P, Ca, Mg, and Mn per cell are presented in Figure 5. Similar consumption profiles for B, Mo, Zn, and Cu are summarized in Figure S15. These figures show that the demands for mineral nutrients per cell produced were higher during the exponential phase of culture growth (e.g., up to day 8). Using consumption data for day 4 as a reference (Figure 5 and Table S7), nutrient demands of P, Ca, Mg, and Mn offer additional information. The average P demand of cells grown in BG11_NO3_ and BG11_N2_ media was similar. However, the average P demand per cell in BG11_urea_ cultures was almost two times the P demand per cell in BG11_NO3_ and BG11_N2_ cultures. For all types of media, high Fe level (5.0 mg/L) also maximized P-consumption per cell. On day 4, the P consumption per cell in BG11_urea_ cultures was at least 2.5 times higher with Fe 0.3 mg/L and Fe 5.0 pmg/L compared to Fe 1.2 mg/L. Compared to BG11_NO3_ and BG11_N2_ cultures, higher average Ca-demands per cell were observed for cells grown in BG11_urea_ (~2 times more) (Table S7). Compared to cultures in BG11_urea_ with Fe 1.2 mg/L, the Ca consumption per cell in BG11_urea_ cultures was ~4.5 times higher with Fe 0.3 mg/L and ~5.5 times with Fe 5.0 pmg/L. Mn consumption increased in all media with Fe 5.0 ppm (See Figure 5). Mg-consumption per cell produced was similar for cultures in BG11_NO3_ and BG11_N2_, especially after day 4. However, the average Mg demand in BG11_urea_ cultures was consistently higher than in BG11_NO3_ and BG11_N2_ (~50% higher on day 4). On BG11_urea_, the highest Mg demands were consistently observed for cultures with low Fe-level (0.3 mg/L). High Mn demands were also evident for BG11_urea_ cultures with both low (0.3 mg/L) and high (5.0 mg/L) Fe levels. On the other hand, the lowest Mn demands were observed for BG11_NO3_ cultures with medium (1.2 mg/L) and low (0.3 mg/L) starting Fe levels. Comparing the data for day 4, the average Mn-demand of BG11_urea_ cultures was at least 2.5 times the Mn required by BG11_NO3_ and BG11_N2_ cultures (See Table S7). The Mn consumption in BG11_urea_ is up to 4 times higher than the Mn demand in BG11_NO3_ and BG11_N2_ when only lower Fe levels are considered (Fe 0.3 mg/L and Fe 1.2 mg/L).

### 3.7. Ranking of Mineral Elements Utilization

**Figure 6:**
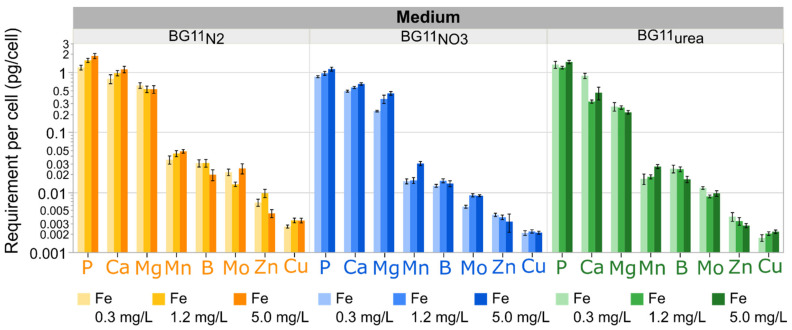
Ranking of mineral element demands per cell. Element demand are calculated based on the cellular production in the exponential phase. P, Ca and Mg are the most important inorganic nutrients, excluding C, Fe, and N-sources. These elements participate in ATP biosynthesis, cellular signaling and Chl*a* production. Mn is essential for photosynthesis and oxidative stress defense. B and Mo are more important for diazotrophic cells in BG11_N2_. Zn and Cu are involved in gene regulation and electron transfer through PSI, respectively. Each bar represents estimated requirements using the derivatives of nutrient consumption expressions with respect to cellular density (Table S8). Error bars represent the confidence fit interval of estimated demands with a significance level α = 0.05. Actual sizes of the error bars may differ due to the logarithmic scale on the Y-axis. Data represent results for three biological replicates per medium type and Fe level combination.

C- and N-sources are the most important nutrients for cyanobacterial growth. However, it is possible to determine an extended nutrient hierarchy based on the amount of element required per cell produced during the exponential phase. The consumption profiles presented in Figures S12 and S13 were expressed in terms of cells produced instead of time. These profiles were used to estimate element requirements per cell in the exponential phase. Transformed linear regression was used to fit element consumption data to cell density change over time. Regression equations are summarized in Table S8. The derivatives of these expressions with respect to cell density were used to estimate nutrient demands with a reference cell density change of 1 × 10^6^ cells/mL, which was typical during the first 6 days of growth in all cultures (See Figure 1). Element demands, as well as the effect of starting Fe concentration in each growth media, are summarized in Figure 6. Considering the vast differences in mineral element demands, a logarithmic scale was used to present all nutrient requirements on the same plot.

The effect of varying Fe levels on the consumption of P, Ca, and Mg is not similar for all types of media, but P and Ca requirements per cell increased for BG11_N2_ and BG11_NO3_ cultures with higher Fe levels. While Mg consumption was not evidently affected by Fe levels in BG11_N2_, the requirements of this element were affected in BG11_NO3_ and BG11_urea_ media (non-diazotrophic metabolism). These data demonstrate that the mineral nutrient consumption is affected by N and Fe metabolism in *Anabaena*, probably because of changing cellular processes during diazotrophic and non-diazotrophic metabolism. A second group of mineral elements is composed by Mn, B and Mo. As previously presented, higher Mn demands are associated with increasing Fe-levels in BG11_N2_, BG11_NO3_, and BG11_urea_. Interestingly, B requirements were negatively affected by increasing Fe levels in BG11_N2_ and BG11_urea_ cultures. Figure 6 successfully captures the amplified importance of B and Mo in BG11_N2_ cultures. Zn and Cu appeared at the end of the nutrient consumption ranking. Figure 6 also shows that Zn consumption was lowest with urea in Fe sufficient conditions (BG11_urea_ medium with Fe 1.2 mg/L and 5.0 mg/L).

## 4. Discussion

### 4.1. Faster Growth Rates and Higher Biomass Production Are Observed in BG11_NO3_ Cultures

Batch cultures of *Anabaena* were grown in three types of growth medium (i.e., BG11_N2_, BG11_NO3_ and BG11_urea_) using N_2_, NaNO_3_ and urea as N-sources, respectively. While N_2_ is the most abundant component of the atmosphere, nitrates are the most abundant form of dissolved inorganic N in surface and deep-water bodies. Urea, a typical component of the urine of mammals, is often used as a fertilizer and is abundant in agro-industrial wastewaters. Ammonium (NH_4_^+^) was not considered because it is rapidly metabolized by multiple microorganisms and it is notably less abundant in water than nitrates [4]. Even when *Anabaena* cells can successfully metabolize each N-source, differences in growth rates and biomass production suggest that dissimilar metabolic stress phenomena in the growth media affect pigment production and nutrient consumption. Overall, abundance of PBPs and β-carotene were affected by the N-source and the initial elemental Fe concentration in *Anabaena* cultures. While CO_2_ fixation rates were mostly affected by the medium N substrate, consumption of phosphorus and micronutrients (e.g., Ca, Mn, B, and Zn) was also affected by the Fe availability in the growth media.

In this work, the standard formulation of BG11 medium was used as a starting point to design growth media for pigment production in *Anabaena* because media with high N:P ratios can be used for increased pigment production in microalgae and cyanobacteria [58,59,60]. Additional details regarding medium formulation are presented in the Supporting Information. For the BG11_urea_ medium, preliminary experiments were conducted in a medium with 9 mM urea, supplying comparable molar N levels to BG11_NO3_ medium [13]. However, such levels of urea led to inconsistent growth kinetics and rapid cellular death. The BG11_urea_ medium was defined after lowering the urea concentration to 3 mM and including compulsory buffering with TES-NaOH at pH 8.0. Cultivation of *Anabaena* with urea is not a trivial task, given the increased metabolic stress arising from the utilization of this nutrient [39]. *Anabaena* cultures were routinely cultivated in the laboratory ensuring complete adaptation to grow in BG11_N2_, BG11_NO3_ and BG11_urea_ media (see Section 2.1). *Anabaena* was originally maintained in BG11(N^-^) medium to preserve the heterocyst-forming phenotype, which can be lost over time after prolonged cultivation in N-rich media [61,62,63]. BG11_N2_ pre-cultures were directly prepared from diazotrophic cultures in BG11(N-), but BG11_NO3_ and BG11_urea_ cultures were prepared from precultures that had already been adapted to grow in media with sodium nitrate and urea for more than one month. The presence of heterocysts was verified with optical end electronic microscopy. SEM was used to observe the presence of heterocysts in BG11_N2_ cultures with more detail (See Figure S1). Although inactive heterocysts can be occasionally found in N replete cultures (e.g., BG11_urea_ or BG11_NO3_), these do not perform N_2_ fixation because of nitrate and ammonium induced inhibition of nitrogenase [64,65,66,67]. The starting Fe levels were selected after considering reports on Fe^3+^ concentrations that allow cellular growth under Fe-depleted (0.3 mg/L) and Fe-surplus (5.0 mg/L) conditions [68]. Although *Anabaena* can slowly grow in liquid media with 20 to 50 mg/L of elemental Fe, these organisms are normally grown in mineral medium with Fe 1.2 mg/L [13,68,69]. Maximum growth rates for *Anabaena* have been reported in culture media with Fe 2.8 mg/L [68]. Given that the growth rates are lower above this concentration, it is implied that the damaging effects of Fe begin to outweigh the benefits of increased Fe availability. In this study, maximum growth rates were observed for cultures in BG11_NO3_ medium, regardless of the Fe level. However, *Anabaena* cultures in BG11_urea_ grew faster with starting Fe-levels between 1.2 and 5.0 mg/L. A significant increase in growth rate was observed for BG11_N2_ cultures with Fe 5.0 mg/L, probably because higher Fe availability favors N_2_ fixation in diazotrophic cyanobacteria [12]. Higher growth rates were also associated with higher biomass production in *Anabaena* cultures (highest biomass accumulation in BG11_NO3_ cultures). Although similar growth rates were observed in BG11_N2_ and BG11_urea_, cell biomass in BG11_N2_ was significantly lower. This can be explained by the fact that diazotrophic cyanobacteria release exopolysaccharides (EPS) to their surroundings when N_2_ is used as their main N-source [70,71]. The C:N ratio of BG11_N2_ media was significantly higher than the C:N ratio of BG11_NO3_ and BG11_urea_ media (Table S3), which can promote carbohydrate production and EPS liberation in *Anabaena* and *Nostoc* species [72]. Since released EPS can affect the turbidity of cultures (Abs_730_), carbohydrate release can also explain the differences in cell density and biomass prediction methods presented for diazotrophic and non-diazotrophic *Anabaena* (See Figure S2).

### 4.2. Low Fe Levels Amplify Accumulation of PBPs

The cellular abundance of PBPs was higher in BG11_N2_ and BG11_urea_ cultures than in BG11_NO3_ cultures. On average, BG11_N2_ cultures developed 31 ± 2.5% more PBPs than BG11_NO3_ cultures. Similarly, BG11_urea_ cultures developed 44 ± 6.5% more PBPs than BG11_NO3_ cultures. Cells in BG11_N2_ and BG11_urea_ also presented slower growth rates. Increased abundance of PBPs in cultures with lower growth rates results from a tradeoff in microbial cellular economics, where slower growth rates are associated with more energetically efficient synthesis of complex molecules [73]. In this regard, higher doubling times allowed the formation of more phycobilisomes (PBSs) before another replication event. The composition of PBPs was also affected by the N-source. Although BG11_N2_ only contained negligible quantities of elemental N (Table S3), heterocysts supplied sufficient levels of N_2_ fixation to sustain the base N demand required for diazotrophic metabolism. As a result, BG11_N2_ cultures effectively produced PBPs, which also act as special N-reserves of cyanobacteria [30]. In average, the PBPs composition of BG11_N2_ cultures was ~75% *w*/*w* CPC, ~16% *w*/*w* APC and ~9% *w*/*w* PEC. In BG11_NO3_ medium, this composition changed to ~73% *w*/*w* CPC, ~28% ***w*/*w*** APC, and ~1% *w*/*w* PEC, exhibiting a dramatic inhibition of PEC biosynthesis. In BG11_urea_ medium, the average PBP composition was ~63% *w*/*w* CPC, ~35% *w*/*w* APC, and ~2% *w*/*w* PEC. These differences in composition are responsible for the changes in cell pigmentation, which are directly associated with the N-source metabolism or the chromatic acclimation mechanisms in the PBSs [30]. The differences in pigmentation of BG11_NO3_, BG11_N2_, and BG11_urea_ cultures are illustrated in Figure S5. While CPC remained the most important PBP in *Anabaena*, regardless of the N-source, PEC gained importance in BG11_N2_, and APC abundance increased in BG11_urea_. Considering that PEC is minimized in non-diazotrophic cultures, regardless of the Fe level, diazotrophic *Anabaena* cultures could be used for large-scale PEC production. In addition, urea-rich wastewater could be used to produce CPC and APC in large-scale operations combining biosynthesis and bioremediation. Although BG11_NO3_ contained 3 times more elemental N than BG11_urea_, the cellular abundance levels of PBPs in these media were similar. This suggests that high N:P ratios in the growth medium do not increase the pigment production indefinitely. Instead, N:P ≤ 20 would be sufficient for efficient large-scale pigment production in *Anabaena*.

Increasing Fe availability resulted in consistent reduction of the average cellular abundance of CPC and APC in *Anabaena* cultures. Higher Fe levels reduced the average cellular abundance levels of CPC and APC in BG11_NO3_, at least during the early stages of growth (Figure 2, up to day 10). These results agree with semi-quantitative reverse transcription-polymerase chain reaction (PCR) data suggesting downregulation of tetrapyrrole and phycocyanobilin biosynthesis (i.e., the PCB chromophore) in wild-type *Anabaena* at low levels of FurA redox-sensing transcription factor in Fe replete conditions. Under low Fe availability conditions, high FurA levels increase the transcript abundance of Heme oxygenases I and II (EC 1.14.99.3), promoting the synthesis of PCB, which is the blue pigment of CPC and APC proteins [12]. As cells reproduced over time, Fe became scarcer on a per-cell basis, leading to upregulation of PCB production at later stages of cell growth. In BG11_N2_ cultures, CPC abundance was also higher at lower Fe-levels, but PEC and APC abundance values were not affected by changing Fe levels. In BG11_urea_, low Fe levels (initial Fe 0.3 mg/L) also amplified the production of PCB, which is the only chromophore in CPC and APC. Low Fe availability generated a remarkable increase of APC accumulation in BG11_urea_. These results also agree with RNA-Seq experiments presenting transcription levels of genes encoding the subunits α and β of APC (*alr0021* and *alr0022*) in *Anabaena.* Transcript abundance of these genes in medium with NH_4_^+^, a product of intracellular and extracellular degradation of urea, was two times the transcription level in diazotrophic cells [74,75,76].

### 4.3. Carotenoid Composition Is Related to Oxidative Stress

The cellular abundance of Chl*a*, CaroT and β-carotene was principally affected by the N-source in the growth medium. In BG11_urea_, slower Chl*a* production rate (Figure S7) resulted in lower cellular abundance values on the early exponential phase, compared to BG11_N2_ and BG11_NO3_ cultures. In contrast, Chl*a* abundance was almost constant in BG11_N2_ cultures. This can be explained because *Anabaena* have naturally evolved to perform N_2_ fixation and diazotrophic growth represents the most stable metabolic state. Constant production of Chl*a* in diazotrophic cells has been previously attributed to the chlorophyll regulator (ChlR), which activates Chl*a* biosynthesis in anoxic environments [77,78,79]. In *Anabaena*, oxygen-free conditions occur in the heterocysts (N_2_-fixing cells), which are differentiated in BG11_N2_ medium [8,30]. The cellular abundance of CaroT was highest for BG11_N2_ cultures (Figure 3), but the mean abundance of β-carotene was lower in BG11_N2_ than in BG11_NO3_ cultures (Figure 4). This suggests that the conversion of β-carotene into other carotenoids was amplified during diazotrophic growth. β-Carotene transformation was probably due to oxidation, either induced by light-stress (light-induced oxidative stress at low optical densities) or by excessive generation of free-radicals from unstable metabolic processes.

In Figure 4, oxidation of β-carotene can be inferred by observing the echinenone/β-carotene ratios. Echinenone is the most abundant product of β-carotene oxidation in *Anabaena*, mediated by β-carotene ketolase (EC 1.14.99.63) [33,34,35]. Therefore, the signal ratios of Echinenone to β-carotene were used as an indicator of β-carotene oxidation in different growth media. Based on this, lowest β-carotene oxidation corresponds to BG11_NO3_ cultures. On the contrary, the highest β-carotene oxidation was observed in BG11_urea_ cultures. This could have occurred because BG11_urea_ cultures presented lower optical and cellular densities during the exponential growth phase (Figure 1), becoming more vulnerable to light-stress events. Higher β-carotene oxidation in BG11_urea_ cultures is also consistent with previous reports describing that growth on urea as a stressful condition for cyanobacteria, triggering peroxidation and cellular death [39,80]. Although the Echinenone to β-carotene signal ratio decreased over time, this does not necessarily mean a reduction of oxidation levels. Instead, it is possible that other carotenes and xanthophylls are produced by *Anabaena* in BG11_urea_ medium after continued metabolic stress. This is partially exemplified in Figure S8, where a new peak of significant height (m/z = 535.0288) was observed only in methanolic extractions from cells in BG11_urea_. Further analysis is necessary to identify this, but it might correspond to torulene, which participates in the synthesis of myxoxanthophylls [81,82]. Considering that carotenoid composition analysis of *Anabaena* have been mostly performed with cells grown in standard BG11 medium with NaNO_3_ [33,34,35], further analysis is required to determine differences in the carotenoid composition of diazotrophic and non-diazotrophic cells. One important finding of this study is that the cellular abundance of β-carotene is negatively affected in *Anabaena* BG11_urea_ cultures.

Although Chl*a* is the main photosynthetic pigment in PSI and PSII, β-carotene plays an essential role for light-harvesting and defense against light-induced oxidation [10,27,36,37]. Therefore, the β-carotene to Chl*a* ratio was interpreted as an indicator of photodamage protection in the photosystems. Based on this, BG11_NO3_ cultures were better protected against photodamage. Exponential phase cellular densities of BG11_urea_ and BG11_N2_ cultures were lower than cell densities in BG11_NO3_ cultures. This can explain the lower levels of photodamage protection inferred from the β-carotene to Chl*a* ratios. Cells grown in BG11_urea_ medium presented the lowest β-carotene to Chl*a* ratios, which can also be explained by increased β-carotene oxidation. The pheophytin *a* to Chl*a* ratio was used as an indicator of Chl*a* metabolism. Since pheophytin *a* is Chl*a* lacking a central Mg^2+^ atom in the chlorin ring [83], it can be observed either as a precursor or as product of chlorophyll breakdown. A more detailed study on the Chl*a* biosynthesis dynamics in *Anabaena* would be necessary to better explain the high pheophytin *a* to Chl*a* ratios in BG11_NO3_ cultures. However, it is possible that faster growth rates in BG11_NO3_ can be associated with accelerated Chl*a* breakdown or incomplete Chl*a* biosynthesis. This can happen because faster microbial growth rates are associated with less efficient biosynthesis of complex molecules (e.g., Chl*a*) [73]. This could also explain why the pheophytin *a* to Chl*a* ratios were significantly lower, and closer to unity, in BG11_N2_ and BG11_urea_ cultures, which presented slower growth rates than BG11_NO3_ cultures. In general, this analysis suggests that large-scale production of β-carotene (pro-vitamin A) by *Anabaena* would be enhanced in growth media with NaNO_3_.

### 4.4. Growth Medium Composition Affects Nutrient Consumption

Growth media for freshwater cyanobacteria are mainly composed of a nitrogen substrate, phosphates, inorganic carbon, and mineral salts that supply essential metallic micronutrients. While C:N ratios of commonly used media range from 0 to 6.8, N:P ratios can be as high as 77 (See Table S1). Excluding *Spirulina*, which can grow in highly alkaline environments [58], it is common practice to culture freshwater cyanobacteria in growth media with very small quantities of inorganic carbon to promote CO_2_-fixation from the atmosphere (see Table S2). Although C- and N-sources are the most important nutrients, all the components of the growth medium are used to sustain autotrophic metabolism and cell reproduction. In the end, cyanobacteria are sophisticated microbial photosynthetic bio-factories with simple nutrient requirements, most of which are inorganic compounds. Despite this highly simplified description of the cyanobacterial metabolism, lack and availability of some nutrients have important implications for biomass generation and biotechnological applications of *Anabaena*. The composition of growth media needs to be properly assessed to standardize cellular reproduction of microorganisms. Although growth requirements for *Anabaena* cultures are simpler than those of other organisms with biotechnological interest, it is important to learn about nutrient demands in these model cyanobacteria. The consumption rates of CO_2_ were mostly affected by the type of N-source in the growth medium. These rate constants can be used to determine or validate metabolic constraints for mathematical modeling purposes. In average, CO_2_ consumption rates of non-diazotrophic *Anabaena* cultures (BG11_NO3_ and BG11_urea_) were 20 ± 5% higher than in diazotrophic cultures (BG11_N2_). These differences can be explained by higher growth rates in BG11_NO3_ and higher activity levels of urease in BG11_urea_. Although *Anabaena* has evolved to utilize atmospheric N_2_ as their main N-source, it has been presented that the molar consumption rates of other N-sources (e.g., nitrate and urea) are significantly faster (Table 2). This can be explained because diazotrophic metabolism demands energetically demanding N_2_-fixation reactions [8], which resulted in a typical diazotrophic growth regime of BG11_N2_ cultures. The exponential-phase N_2_-fixation rate of BG11_N2_ *Anabaena* cultures (0.06 mmol N_2_ L^−1^ day^−1^, Table 2) corresponds to a cellular density of ~1.2 × 10^6^ cells/mL (Figure 1, day 4) and Chl*a* concentration of ~2.5 μg/mL (Figure S7, day 4). This translates into a call-specific N_2_ fixation rate of 3.8 fmol-N cell^−1^ h^−1^ or 0.92 nmol-N_2_ (μg Chl*a*) ^−1^ h^−1^. These values are comparable to cell-specific N_2_-fixation rates of N_2_ fixing cyanobacteria, either cultured under controlled laboratory conditions or in their natural habitats [84,85,86,87]. For additional details, refer to Table S10, which compares cell-specific N_2_ fixation rates for different cyanobacteria.

Besides C- and N-sources, cyanobacterial growth media contained other mineral elements (e.g., B, Na, Mg, P, K, Ca, Mn, Fe, Co, Ni, Cu, Zn and Mo). From these, the mineral demands of P, Ca, Mg, Mn, B, Mo, Zn, and Cu were higher during the early exponential phase (Figure 5 and Figure S15). Concentration and consumption profiles of these elements in *Anabaena* cultures were assessed with a multi-element ICP-OES detection method (Section 2.6). The election of Fe availability as a factor impacting pigment production and nutrient consumption in *Anabaena* was supported by the vital role of this element in cyanobacteria and its involvement in the dynamic behavior of the master global regulator FurA [7,9,10,11,12,16]. Fe is extremely valuable for cyanobacteria, as occurs for any bacterial species [10,11]. Fe consumption profiles presented in Figure S11 indicated that the starting population of *Anabaena* cells in each culture immediately incorporated this nutrient, activating siderophores and bacterioferritin mediated mechanisms of Fe accumulation [6,11,88]. These profiles confirm the fact that cyanobacteria will incorporate as much Fe source as is available in the growth medium [11]. Given that no extra Fe source was added to the cultures during the growth experiments, global Fe concentrations were constant in the batch experiments. Moreover, the initial Fe reserves had to be distributed from mother to daughter cells during reproduction.

Excluding C- and N-sources, the most important mineral elements in cyanobacterial growth medium are P, Ca, Mg, and Mn (see ranking in Figure 6). This is not surprising because these elements participate in essential metabolic processes. While P is incorporated in the phosphate groups of ATP, NADPH, DNA, RNA and membrane lipids, Ca plays a pivotal role in cell signaling [30,89]. Although Mg is necessary for the biosynthesis of Chl*a*, this versatile element is also involved in nucleic acid stabilization, circadian rhythm regulation, DNA replication, ribosomal stabilization, and regulation of RuBisCo (ribulose-1,5-bisphosphate carboxylase/oxygenase, EC 4.1.1.39) [10,90,91]. Mn is an essential micronutrient in cyanobacterial growth media because it is present in the Mn_4_CaO_5_ cluster of PSII, which is responsible for O_2_ evolution and electron transfer from water [22,23]. Remarkably, Mn is also involved in the defense mechanisms of *Anabaena* against oxidative stress, as this element is present in Mn catalase (EC 1.11.1.6) and Mn superoxide dismutase (E.C. 1.15.1.1) [92,93]. These enzymes are responsible for the degradation of reactive oxygen species (ROS, i.e., O_2_^−^ and H_2_O_2_) produced during photosynthesis [21,24,25].

Figure 5 indicates that cellular P-demands were initially higher for cultures in BG11_NO3_ and BG11_urea_. After day 4, the cellular P-demands remained significantly high for BG11_urea_ cultures. Higher P-demands in fast-growing BG11_NO3_ cultures are understandable because phosphates are essential for synthesis of DNA, RNA, lipids, carbohydrates, and proteins during biomass generation. In BG11_urea_ cultures, higher P demands may be interpreted as a sign of higher enzymatic activity. In general, higher P demands in non-diazotrophic media, are consistent with several studies dealing with coupled N and P consumption in eutrophic waters [94,95,96,97]. The lower P demand in BG11_urea_ with Fe-1.2 mg/L might suggest that these cultures experience lower metabolic stress. In BG11_urea_ cultures with high (5.0 mg/L) and low (0.3 mg/L) initial Fe levels, higher ATP requirements can be related to increased activity of defense mechanisms against peroxidation [39]. Although high Ca demands were observed in BG11_urea_, increased Ca demands with low and high Fe-levels are not necessarily related to more frequent Ca^2+^-mediated signaling triggered during cellular stress [89]. While multiple interconnected metabolic processes could explain higher Mg consumption in non-diazotrophic cells, high Mn demand may be related to increased oxidative stress promoted by ROS in *Anabaena* [10,12,15]. Low levels of Mn consumption per cell in BG11_NO3_ and BG11_N2_ with Fe 1.2 and Fe 0.3 mg/L, indicate reduced oxidative stress when NaNO_3_ or N_2_ are used as N-sources. High Mn requirements in BG11_urea_ cultures can be interpreted as increased levels of oxidative stress (Table S7). Increased Fe availability also amplified Mn requirements for all growth media. Analysis of consumption of other micronutrients in the growth media are presented in the supporting information.

## 5. Conclusions

Selection of a suitable N-source and definition of Fe levels in the growth media are fundamental parameters to analyze pigment production and nutrient consumption by *Anabaena* cultures. N-source type, Fe availability and oxidative stress events can be manipulated to maximize the efficiency of a desired biotechnological application for *Anabaena*. While diazotrophic *Anabaena* cultures can be used for maximum accumulation of phycoerythrocyanin (PEC), growth media with NaNO_3_ and urea (N:P ≤ 20) are recommended for C-phycocyaninin (CPC) and allophycocyanin (APC) production, respectively. Here, CPC and APC accumulation was enhanced by low initial Fe levels (0.3 mg/L). Low oxidative stress in growth medium with NaNO_3_ enhance the bioproduction potential of β-carotene, a commercially important precursor of vitamin A. For cyanobacteria-mediated bioremediation of wastewater with high loads of urea and phosphates, high oxidative stress could affect the carotenoid composition of non-diazotrophic cultures. Although *Anabaena* can metabolize multiple N-sources, diazotrophic metabolism (N_2_ fixation) results in most stable production of Chl*a* and higher cellular abundance of phycobiliproteins (PBPs).

N-source type and Fe availability also affected the consumption of mineral elements (e.g., P, Ca, Mg, Mn, B, Mo, Zn, Cu), which play essential roles on the metabolism of cyanobacteria. Controlling the concentration of all nutrients in the growth medium is extremely important for the development of sustainable cyanobacterial biofactories. Therefore, the development of efficient chemical analysis methods for mineral media represents a significant step forward for standardization of cyanobacteria cultivation at a larger scale. Pigments and vitamins are some of the most valuable biotechnological products of cyanobacteria and their biosynthesis is also influenced by the presence of metallic elements and N-sources. Epigenetic interactions between cyanobacteria and metals represent a highly under-explored research field that could complement the field of synthetic biology. The results presented in this paper demonstrate the importance of having a systemic interpretation of the cyanobacterial metabolism to take full advantage of versatile organisms like *Anabaena*.

## Figures and Tables

**Table 1:** Growth and biomass generation of *Anabaena* in different growth media. Values represent the average and standard errors from three independent biological replicates per Medium–Fe level treatment. Biomass data correspond to the biomass generated over 14 days of growth. Two-way analysis of variance (ANOVA) was used to analyze statistically significant differences with a significance level α = 0.05. Medium type and Fe-level were used as independent predictors. Medium levels not connected by same group letter are significantly different. Fe-levels not connected by same number of stars within medium type are significantly different. Growth rate (Medium *p*-value = 0.0050; Fe level *p*-value = 0.4102; interaction *p*-value = 0.7536), Generation time (Medium *p*-value = 0.0037; Fe level *p*-value = 0.1419; interaction *p*-value = 0.1918), biomass-14 days (Medium *p*-value < 0.0001; Fe level *p*-value = 0.6376; interaction *p*-value = 0.5518).

Medium/Fe-Level	Growth Rate (h^−1^)	Group	Generation Time (h)	Group	Biomass-14 Days (μg/mL)	Group
a.BG11_N2_	0.02 ± 0.003	A	53.59 ± 7.69	A	312.03 ± 34.24	A
Fe 0.3 ppm	0.018 ± 0.003	*	57.2 ± 7.83	*	285.24 ± 24.94	*
Fe 1.2 ppm	0.018 ± 0.003	*	58.2 ± 8.76	*	291.91 ± 45.59	*
Fe 5.0 ppm	0.023 ± 0.004	**	45.38 ± 6.91	**	358.95 ± 18.05	**
b.BG11_NO3_	0.032 ± 0.006	B	33.61 ± 5.9	B	772.68 ± 88.1	B
Fe 0.3 ppm	0.031 ± 0.006	*	34.38 ± 6.97	*	784.38 ± 137.21	*
Fe 1.2 ppm	0.033 ± 0.006	*	32.6 ± 5.23	*	746.69 ± 60.11	*
Fe 5.0 ppm	0.033 ± 0.008	*	33.85 ± 7.92	*	786.96 ± 89.99	*
c.BG11_urea_	0.019 ± 0.004	A	59.87 ± 13.09	A	474.33 ± 94.18	C
Fe 0.3 ppm	0.013 ± 0.001	*	80.67 ± 8.78	*	384.46 ± 28.05	*
Fe 1.2 ppm	0.024 ± 0.003	**	43.91 ± 7.05	**	585 ± 85.7	**
Fe 5.0 ppm	0.021 ± 0.005	**	55.04 ± 14.26	**	453.52 ± 130.26	*

**Table 2:** Rate constants for C- and N-source consumption. Total organic carbon (TOC) formation rates represent the zero-order rate constant. N-source consumption is shown in Figure S9. Equivalent CO_2_ consumption rate was calculated by multiplying TOC formation rate by 3.67. Numbers represent averages and standard deviations for consumption/ fixation rates considering at four biological replicates per medium type and a total of 56 TOC measurements.

Formation or Consumption Rates	BG11_N2_	BG11_NO3_	BG11_urea_
TOC formation rate (mg L^−1^ day ^−1^)	14.5 ± 1.1	17.2 ± 0.7	18 ± 1.3
CO_2_ consumption rate (mg L^−1^ day ^−1^)	53.2 ± 4	63.1 ± 2.6	66 ± 4.8
CO_2_ consumption rate (mM day ^−1^)	1.2 ± 0.09	1.4 ± 0.06	1.5 ± 0.11
N-source consumption rate constant, *k* (day ^−1^)	0.21 ± 0.013	0.06 ± 0.004	0.08 ± 0.012
N-source consumption rate @ day 4 (mg L^−1^ day ^−1^) ^a^	1.55 ± 0.1	65.8 ± 4.4	11.87 ± 1.8
N-source consumption rate @ day 4 (mM day ^−1^)	0.06 ± 0.004	0.77 ± 0.05	0.2 ± 0.03

^a^: First-order consumption rates change in time. The reaction rate at a given time (e.g., day 4) is *k* times the N-source concentration at a given time point (note the logarithmic scale in Figure S9).

## Data Availability

Data is contained within the article or Supplementary Materials. Additional information is available upon request from the corresponding author.

## Supplementary Materials

The following are available online at https://www.mdpi.com/2076-2607/9/2/431/s1, Figure S1. SEM picture of diazotrophic filaments of *Anabaena*; Figure S2. Correlation for estimating *Anabaena* biomass concentration and cell density from apparent absorbance; Figure S3. Normalized growth data with saturation kinetic model; Figure S4. Growth rates and generation times of *Anabaena* cultures in different media; Figure S5. Pigmentation of *Anabaena* cultures; Figure S6. Concentration of PBPs in media with different N-sources; Figure S7. Concentration of Chl*a* and CaroT in different growth media; Figure S8. Mass-spectrometry time-of-flight (MS-TOF) analysis of methanol extracts; Figure S9. Consumption rates of CO2 and N-sources; Figure S10. *Anabaena* urease activity in different media; Figure S11. Fe consumption profiles in growth media; Figure S12. Consumption profiles of P, Ca, Mg and Mn; Figure S13. Consumption profiles of B, Mo, Zn and Cu; Figure S14. Concentration profiles of Na, K, Ni and Co; Figure S15. Consumption profiles of B, Mo, Zn and Cu per cell; Figure S16. Calibration curve for quantification of β-carotene; Figure S17. Calibration curve for urea quantification; Figure S18. Inductively coupled plasma optical emission spectroscopy (ICP-OES) calibration for elemental analysis of BG11 media; Table S1. Properties of freshwater and marine growth media for microalgae; Table S2. C:N ratios of growth media used for laboratory and large-scale cultures of cyanobacteria; Table S3. Properties of fresh growth media; Table S4. Concentration of mineral elements in growth media; Table S5. PBPs abundance per cell in different growth media Concentration of mineral elements in growth media; Table S6. Chl*a*, CaroT, and β-carotene abundance per cell in different growth media; Table S7. P, Ca and Mn requirements per cell produced in different growth media; Table S8. Regression equations for mineral consumption; Table S9. Element concentrations of calibrations standards for ICP-OES; Table S10. N_2_-fixation rates of diazotrophic cyanobacteria.
