# Supplementary material for: Nitrogen Sources and Iron Availability Affect Pigment Biosynthesis and Nutrient Consumption in Anabaena sp. UTEX 2576
Source: Microorganisms. 2021 Feb 19;9(2):431. doi: 10.3390/microorganisms9020431 (PMC7922959; doi:10.3390/microorganisms9020431)
Supplement: Supplementary file 1 [file microorganisms-09-00431-s001.pdf]

Nitrogen sources and Iron availability affect pigment biosynthesis and nutrient consumption in *Anabaena* sp. UTEX 2576

Daniel A. Norena-Caro, Tara M. Malone and Michael G. Benton*

Cain Department of Chemical Engineering, Louisiana State University and A&M College, Baton Rouge, Louisiana, United States of America, 70803

*For correspondence (e-mail: benton@lsu.edu, phone: +1-225-578-3056)

SUPPORTING INFORMATION

Supplementary methods and results

Selection of culture medium

Anabaena are freshwater cyanobacteria with lower ionic requirements compared to marine or estuarine species [1,2]. As opposed to coastal marine microalgae and cyanobacteria, which are commonly cultured in ASN-III, f/2, or L1 media, axenic cultures of freshwater cyanobacteria are best grown in BG11, Chu-10, Zarrouk, or Bold's Basal media [3]. From these, BG11 medium is widely used for laboratory-scale cultivation, even at the largest and most recognized collections of cyanobacteria like the Pasteur Culture Collection (PCC), the American Type Culture Collection (ATCC), and the Culture Collection of Algae at the University of Texas (UTEX) [2,3]. BG11 medium was specifically designed to maintain large collections of freshwater, soil, and thermophilic "Blue-Green algae", by changing the original formulation of medium number 11 [4]. Like any other growth medium, BG11 contains macronutrients, trace elements, and vitamins [2,3]. The main feature of BG11 medium is that the N:P ratio is deliberately high (~ 80:1) for simple and convenient cultivation of unicellular and filamentous cyanobacteria [2,3,5]. However, species-specific medium optimization is necessary for a given biotechnological application [2,6]. For heterocyst-forming cyanobacteria like *Anabaena*, the main Nitrogen source ($NaNO_3$) is completely removed from the BG11 medium formulation to preserve the N_2-fixation ability. Thus, BG11(N-) is commonly used for culturing diazotrophic cyanobacteria. The main text describes additional alterations of the standard BG11 medium considered in this project. For additional reference, Table S1 compares the composition of different media used for culturing algae and cyanobacteria. Note that C:N ratios in Table S1 are calculated from C and N-source concentrations of the base formulation. The C:N ratios change in the final growth medium if additional carbonates or bicarbonates are added. This especially applies to f/2 and L1 media that are normally prepared with sea water (natural or artificial), which adds additional bicarbonate to the growth medium (~2 mM) [3].

Table S1. Properties of freshwater and marine growth media for microalgae

Table presents chemical composition of commonly used media for cyanobacteria and microalgae. BG11, Bold'd basal medium (BBM), Chu-10, and Zarrouk medium (ZM) are commonly used for freshwater cyanobacteria. ASN-III, f/2, and L1 are commonly used for marine microalgae and cyanobacteria [3,7].

	Concentration in final medium (mM)						
Chemical compound	BG11	BBM	ZM	Chu-10	ASN-III	f/2	L1
$Ca(NO_3)_2 \cdot 4H_2O$	0	0	0	8.5E-03	0	0	0
$NaHCO_3$	0	0	200.0	0	0	0	0
$NaNO_3$	17.6	2.9	29.4	0	8.8	0.9	0.9
KH_2PO_4	0	1.3	0	4.6E-03	0	0	0
K_2HPO_4	0.2	0.4	2.9	0	4.3	0	0
$NaH_2PO_4 \cdot H_2O$	0	0	0	0	0	3.6E-02	3.6E-02
$MgSO_4 \cdot 7H_2O$	0.3	0.3	0.8	1.0E-02	14.2	0	0
$MgCl_2 \cdot 6H_2O$	0	0	0	0	9.8	0	0
$CaCl_2 \cdot 2H_2O$	0.2	0.2	0.3	0	3.4	0	0
NaCl	0	0.4	17.1	0	427.8	0	0
Na_2CO_3	0.2	0	0	1.9E-02	0.2	0	0
$Na_2SiO_3 \cdot 9H_2O$	0	0	0	8.8E-03	0	0.1	0.1
Na_2EDTA	2.7E-03	0.3	2.1E-04	5.4E-04	0	1.2E-05	1.2E-02
MgEDTA	0	0	0	0	1.4E-03	0	0
$FeCl_3 \cdot 6H_2O$	0	0	0	3.7E-04	0	1.2E-05	1.2E-02
KOH	1.1	0	0	0	0	0	0
H_3BO_3	0	1.9	0	4.0E-03	0	0	0
$FeSO_4 \cdot 7H_2O$	1.8E-01	3.6E-02	0	0	0	0	0
$(NH_4)_6Mo_7O_{24} \cdot 4H_2O$	0	0	0	8.1E-05	0	0	0
K_2SO_4	0	0	5.7	0	0	0	0
$MnCl_2 \cdot 4H_2O$	0	0	0	7.0E-04	0	9.1E-07	9.0E-04
$ZnSO4 \cdot 7H_2O$	0	0	0	0	0	7.7E-08	8.0E-05
$CoCl_2 \cdot 6H_2O$	0	0	0	0	0	4.2E-08	5.0E-05
$CuSO_4 \cdot 5H_2O$	0	0	0	0	0	3.9E-08	1.0E-05
$Na_2MoO_4 \cdot 2H_2O$	0	0	0	0	0	2.6E-08	8.2E-05
Citric acid	2.9E-02	0	0	0	1.4E-02	0	0
Ferric ammonium citrate	2.3E-02	0	0	0	1.1E-02	0	0
Vitamin B12	7.4E-07	7.4E-07	7.4E-07	7.4E-07	7.4E-06	3.7E-10	1.8E-07
Thiamine HCl	3.0E-07	3.0E-07	3.0E-07	3.0E-07	0	3.0E-04	1.5E-04
Biotin	4.1E-07	4.1E-07	4.1E-07	4.1E-07	0	2.0E-06	1.0E-06
Trace metal mix A5 (mL)	1	1	1	0	1	0	0
pH	7.2	7.5	10	7.6	-[a]	-[a]	-[a]
Total volume (L)	1	1	1	1	1	1	1
C:N[b]	0.01	-	6.8	1.1	0.02	-	-
N:P	76.9	1.7	10.2	3.7	2.05	24.4	24.4

a: Recommended final pH depends on the pH of the Sea water used as a base.

b: The C:N ratios are affected if additional dissolved inorganic carbon is incorporated in the medium

In general, Table S1 indicates that the standard formulation of cyanobacterial growth media has low C:N ratio (ranging from 0 to 6.8). According to the “Redfield ratio”, the ideal C:N ratio for maximum growth of phytoplankton should be between 6 and 7 [8]. However, a C:N ratio in this range is observed only for Zarrouk medium, which is commonly used for culturing *Spirulina* (*Arthrospira*) [7,9]. This is possible because *Spirulina* filaments thrive on growth media with high pH, which results from keeping a significantly high concentration of sodium bicarbonate (NaHCO3, 200 mM) [3]. For other cyanobacterial species like *Anabaena*, such levels of bicarbonate in the growth medium are prohibitive because optimal culture pH lies between 7.5 and 8.5 [10,11]. For massive culturing of these cyanobacteria, the addition of zwitterionic buffering agents (e.g., Good’s buffers) for pH control is not normally regarded as a solution to maintain very high concentrations of dissolved inorganic carbon (DIC) [12]. Instead, recent efforts to develop autotrophic cyanobacterial biofactories have employed growth media with C:N ratios outside the ideal “Redfield ratio” range. Table S2 presents the C:N ratio of growth media used for laboratory and large-scale cultures of cyanobacteria. Depending on the species and its ability to perform N_2-fixation, the growth medium C:N ratio of large-scale preparations of cyanobacteria can be as low as 0.003:1, or as high as ~2000:1. In the case of diazotrophic cultures, the medium C:N ratio is excessively high because the liquid only contains negligible quantities of elemental Nitrogen from iron or trace element additives.

The N:P ratio of the growth medium is another important factor for cyanobacterial culturing. Depending on the desired application, the N:P ratio may be modified to either promote biomass generation or lipid accumulation for biofuel applications. While N:P ratios above 30:1 can limit growth because of Phosphorus insufficiency, N:P ratios below 10:1 have been explored to promote fast accumulation of lipids [6]. In this work, we analyze the effect of different N-sources and Fe-levels on pigment production by *Anabaena*. In this regard, high pigment production has been observed in cyanobacteria grown in media with very high N:P ratios [10,11,13]. Considering that this study also aims to quantify the impact of the N-source on CO_2-fixation, the initial C:N ratio of the growth medium needed to be kept at the minimum. Therefore, BG11 formulation was selected as the base growth medium to conduct the experiments. Given that *Anabaena* is a versatile user of nitrogenous compounds, the growth media considered in this work are variants of standard BG11, where dinitrogen ($BG11_{N2}$), sodium nitrate ($BG11_{NO3}$), or urea ($BG11_{urea}$) are regarded as the main N-sources.

Table S2. C:N ratios of growth media used for laboratory and large-scale cultures of cyanobacteria

Table presents recent applications of cyanobacteria for CO_2 sequestration and some details of the culture growth medium. In these applications, the C-sources are atmospheric CO_2 in equilibrium with the liquid and added Na_2CO_3 or $NaHCO_3$. For non-N2-fixing species, nitrates are commonly used as N-source. For N_2-fixing species, growth media contain only slight quantities of elemental N from Fe or trace element additives.

Cyanobacteria	N_2-fixing	CO_2 (%v/v)	CO_2 eq (mM)[a]	Na_2CO_3 or $NaHCO_3$ (mM)	Medium	Medium N-source [c]	Total N conc. (mM)	C:N ratio	Ref.
Aphanothece microscopica Nageli	No	15.0%	4.61	0	BGN	$NaNO_3$	1764.7	0.003	[14]
Synechocystis aquatilis	No	10.0%	3.07	59.5	SOT	$NaNO_3$	47.1	1.33	[15]
Synechocystis sp.	No	10.0%	2.34	0	MM-F	$NaNO_3$ and $Ca(NO_3)_2$	8.5	0.28	[16]
Anabaena sp. ATCC 33047	Yes	0.04%	0.01	50	-[b]	$(NH_4)_5[Fe(C_6H_4O_7)_2]$	0.02	2183.70	[17]
Spirulina platensis UTEX LB 2340	No	2.0%	0.67	200	Zarrouk	$NaNO_3$	29.4	6.83	[18]
Anabaena sp.	Yes	10.0%	4.12	0	Arnon	NH_4VO_3	0.02	209.30	[19]
Nostoc sp. PCC 7120	Yes	5.0%	1.45	0.19	BG11(N-)	$(NH_4)_5[Fe(C_6H_4O_7)_2]$	0.02	71.62	[20]
Synechococcus elongatus PCC 7942	No	5.0%	1.54	0.19	BG11	$NaNO_3$	17.6	0.10	[21]
Microcystis icthyoblabe	No	10.0%	3.35	0	CB	KNO_3 and $Ca(NO_3)_2$	2.3	1.5	[22]
Microcystis aeruginosa	No	10.0%	3.35	0	CB	KNO_3 and $Ca(NO_3)_2$	2.3	1.5	[22]
Spirulina platensis LEB-52	No	5.0%	1.54	200	Zarrouk	$NaNO_3$	29.4	6.85	[23]
Synechocystis salina	No	10.0%	3.72	0.60	-[b]	$NaNO_3$	2.9	1.47	[24]

a: CO_2 in equilibrium with the liquid was calculated with Henry's law using a reference Henry's constant $K°_H$ of 0.035 mol L^{-1} bar $^{-1}$ at 298.15 K and the CO_2 partial pressure at the experiment conditions

b: The study did not use a standard medium, but a personalized formulation

c: For N_2-fixing species, the main N-source is N_2, but the medium also supplied slight amounts of other N-compounds

Heterocyst-formation verification

Axenic cultures of *Anabaena* were consistently kept in our laboratory. Verification of heterocyst formation for N_2-fixation was conducted with optical and scanning electron microscopy (SEM). For SEM, *Anabaena* cells were fixed and dried for observation with a JSM-6610 scanning electron microscope. The method was adapted from a standard protocol used for *Chlamydomonas reinhardtii* at the Louisiana State University shared instrumentation facility (SIF) [25,26]. Filaments from pre-cultures in BG11(N-) media were mixed with a solution of 1%v/v osmium tetraoxide (OsO4), 2% v/v formaldehyde and 2% v/v glutaraldehyde. The mixture was drawn through a 13 mm-diameter, 0.2 µm pore-size polycarbonate filter using a 10-mL syringe with a Swinney filter holder. Filtered cells rested for 2 hours to promote fixation onto the filter. Then, cells were rinsed twice with deionized (5 minutes per wash), *en bloc* stained with 0.5% uranyl acetate for 30 minutes in the dark and rinsed again with DI water twice. Dehydration was performed with sequential ethanol wash, rinsing first with 50%v/v ethanol for 15 minutes, then with 67% v/v ethanol for 15 minutes, and twice with 100% ethanol at the end of the series. Hexamethyldisilazane (HMDS) was used for critical point drying of biological specimens. Cells were first rinsed with 50% v/v HMDS and 50% v/v ethanol for 15 minutes, then with 67% v/v HMDS and 33% v/v ethanol for another 15 minutes, and finally twice with 100% HMDS for 15 minutes. After this, cells rested in the syringe for one extra hour. Filters with fixed and dried cells were withdrawn from the syringe and mounted on aluminum SEM stubs. Specimens were coated with gold for 4 minutes at 0.1 mBar vacuum and 25 mA using an Edwards S150 sputter coater. SEM pictures of *Anabaena* filaments were obtained with acceleration voltage of 15 kV and working distance of 12 mm.

The cell diameter distribution of vegetative cells and heterocysts was estimated from *Anabaena* SEM pictures. The diameter of 165 vegetative cells (V) and 193 heterocysts was determined using the reference bar and the size estimation tool from Image J software. Heterocysts could be easily identified in *Anabaena* filaments because they were larger than vegetative cells and occurred every 10 to 20 cells [27]. The average diameter of heterocysts and vegetative cells was estimated from the distribution. Figure S1 presents a typical SEM picture of diazotrophic *Anabaena* filaments. While the mean diameter of vegetative cells was 2.84 ± 0.44 µm, the average diameter of heterocysts was 4.40 ± 0.61 µm. The larger size of heterocysts is due to the heterocyst glycolipid layer (HGL) and the heterocyst envelope polysaccharide (HEP) [11].

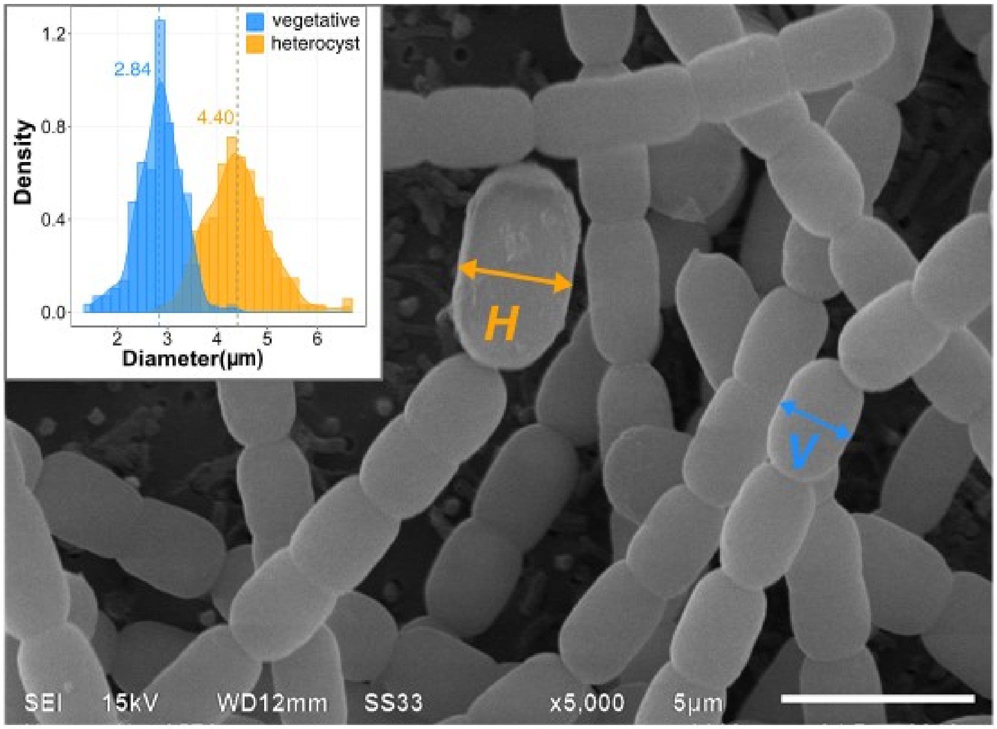

Figure S1. SEM picture of diazotrophic filaments of *Anabaena*.

Heterocysts (H) are larger than vegetative cells (V) due to the protective carbohydrate layers that prevent oxygen from entering these cells.

Changes on growth media formulation

The urea concentration in $BG11_{urea}$ media was limited to 3.0 mM because higher levels led to rapid chlorosis (bleaching) and cellular death within the first two-days of cultivation, regardless of pH control. This observation was consistent with previous observations setting the urea toxicity threshold level for *Anabaena* under 5.0 mM [28,29]. The nominal starting pH of fresh media was adjusted to 8.0 with NaOH or HCl 0.5 N. Buffer addition was not necessary for sustained growth of *Anabaena* in $BG11_{N2}$ nor $BG11_{NO3}$ media, but TES buffer (10 mM) was needed for long term growth in $BG11_{urea}$ medium because the liquid becomes more acidic as urease degrades urea into NH4+ and carbonic acid over time [30,31]. The nominal initial concentration of P (supplied as K_2HPO_4 •$2H_2O$) was 0.27 mM, which is ~20% higher than the standard BG11 concentration (0.23 mM). The N:P ratio was lower than the standard BG11 ratio (77:1), to correct possible growth limitation associated with P starvation [6,8]. Considering that Zn is needed for essential cyanobacterial processes like DNA protection during oxidative stress, gene regulation and carbonic anhydrase activity [32,33], the initial nominal availability of Zn (as $ZnSO_4$•$7H_2O$) was two times higher than in standard BG11 (1.5×10^{-3} mM vs 7.7×10^{-4} mM). Although the standard BG11 formulation does not contain Ni, 1.5 µM $NiSO_4$•$6H_2O$ (0.01 mg/L of Ni) was added to the growth media to ensure a basal level of urease activity in each culture [29].

Regarding $BG11_{N2}$ media, increasing amounts of Ferric ammonium citrate to adjust initial Fe-availability can also be thought of as additional N-sources. Although heterocyst formation can be inhibited by ammonium (NH_4^+, 3 to 7 µM), this only applies when heterocysts have not been formed yet. After the heterocysts have been formed, the activity of Nitrogenase is still maximum in *Anabaena*, even with extracellular NH_4^+ concentrations between 0.6 and 0.8 mM [34,35]. Additionally, continued heterocyst development is almost insensitive to repression by NH_4^+ ions at pH 7-8, requiring higher pH levels (~10) for rapid inactivation of Nitrogenase [36]. In *Anabaena*, illumination also affects the inhibitory effect of NH_4^+. It has been reported that the inhibitory effect of NH_4^+ on heterocyst differentiation is favored at low light intensity (Under 5 µmol m^{-2} s^{-1}). At high illumination (140 µmol m^{-2} s^{-1}), Nitrogenase activity is preserved even at 2 mM NH_4^+ in the environment [37]. In our experiments, $BG11_{N2}$ cultures were prepared from precultures with active heterocysts, the culture pH was around 8, and the illumination level was 75 µmol m^{-2} s^{-1}. Therefore, initial elemental N levels between 0.01 to 0.09 mM (Table S3), supplied by the Ferric ammonium citrate, were not expected to affect the heterocyst-differentiation ability of cells that had already been adapted to grow diazotrophically. Differences in elemental compositions with respect to standard BG11 medium are highlighted in Table S3. The initial concentrations of the N-sources, total organic Carbon (TOC), pH, and levels of mineral elements in fresh growth media were measured for quality assurance purposes (see Table S3 and Table S4).

Table S3. Properties of fresh growth media

Table presents initial concentration of C, N and Fe in growth media. Total organic carbon (TOC) values indicate concentration of organic compounds in fresh media. Initial TOC values of $BG11_{N2}$ and $BG11_{NO3}$ media were negative, indicating negligible traces of organic compounds in these mineral media. TOC values of fresh $BG11_{urea}$ media are attributed to urea and TES-NaOH buffer. Values represent the mean of three replicates and intervals represent the standard deviation.

Medium	Fe level (mg/L)	Main Nitrogen substrate	Theoretical conc. (mg/L)	Theoretical conc. (mM)	Elemental N (mM)	Measured conc. (mM)[b]	Na_2CO_3 conc. (mM)	Equil. CO_2 (mM)[c]	Measured TOC (mg/L)	Measured pH	initial C:N ratio	initial N:P ratio
$BG11_{N2}$	0.3	N_2	0	0	0.01[a]	0	0.19	0.01	-1.8 ± 0.7	8.04 ± 0.01	37.6	0.02
$BG11_{N2}$	1.2	N_2	0	0	0.02[a]	0	0.19	0.01	-0.8 ± 0.4	8.01 ± 0.08	9.4	0.08
$BG11_{N2}$	5.0	N_2	0	0	0.09[a]	0	0.19	0.01	-1.2 ± 0.6	8.01 ± 0.06	2.3	0.33
$BG11_{NO3}$	0.3	$NaNO_3$	1500	17.7	17.7	19.6 ± 3.1	0.19	0.01	-11 ± 2.2	7.98 ± 0.05	0.01	65.4
$BG11_{NO3}$	1.2	$NaNO_3$	1500	17.7	17.7	18.5 ± 4.1	0.19	0.01	-19 ± 2.8	7.98 ± 0.03	0.01	65.4
$BG11_{NO3}$	5.0	$NaNO_3$	1500	17.7	17.7	20.0 ± 2.1	0.19	0.01	-20 ± 3.3	8.03 ± 0.01	0.01	65.7
$BG11_{urea}$	0.3	Urea	180	3.0	6.0	3.3 ± 0.2	0.19	0.01	625 ± 6.3	7.95 ± 0.06	0.03	22.2
$BG11_{urea}$	1.2	Urea	180	3.0	6.0	3.2 ± 0.1	0.19	0.01	616 ± 6.0	7.92 ± 0.03	0.03	22.3
$BG11_{urea}$	5.0	Urea	180	3.0	6.1	3.3 ± 0.3	0.19	0.01	647 ± 7.9	7.97 ± 0.03	0.03	22.6

a: Initial concentration of elemental N in $BG11_{N2}$ media is not zero because they also contain Ferric Ammonium citrate ($C_6H_8FeNO_7$) and 1.7E-4 mM Cobalt Nitrate ($Co(NO_3)_2$)

b: To obtain NaNO3 concentration measurements in mg/L multiply by MW: 84.995 g/mol

c: Equilibrium CO_2 describes maximum aqueous CO_2 in liquid media corresponding to 410 mg/L of CO_2 in atmosphere (Henry's constant @ 301.15 K = 0.032 mol L^{-1} bar

Table S4. Concentration of mineral elements in growth media

Levels of P, Ni, and Zn (in parenthesis) were deliberately higher than in standard BG11 medium. Actual levels of Na and K were affected by pH adjustment. Values represent mean and standard error (N=3).

	Element	Nominal conc. (mg/L)	Conc. in low Fe (mg/L)	Conc. in medium Fe (mg/L)	Conc. in high Fe (mg/L)
Initial concentration in $BG11_{N2}$	B	0.50	0.52 ± 0.03	0.51 ± 0.04	0.47 ± 0.06
	Na	8.87	33.83 ± 2.09	32.7 ± 1.33	32.88 ± 1.94
	Mg	7.40	7 ± 0.41	6.96 ± 0.45	7.01 ± 0.51
	P	7.11 (8.50)	8.74 ± 0.66	8.76 ± 0.59	8.9 ± 0.93
	K	17.96	14.96 ± 0.71	13.95 ± 0.81	14.52 ± 0.69
	Ca	9.81	10.59 ± 1.04	10.28 ± 0.75	10.63 ± 1.14
	Mn	0.50	0.55 ± 0.12	0.56 ± 0.1	0.52 ± 0.13
	Fe	1.28	0.27 ± 0.01	1.17 ± 0.19	5.68 ± 0.55
	Co	0.01	0.009 ± 0.01	0.01 ± 0.003	0.001 ± 0.01
	Ni	0.00 (0.01)	0.011 ± 0.003	0.011 ± 0.003	0.011 ± 0.003
	Cu	0.02	0.017 ± 0.003	0.018 ± 0.003	0.022 ± 0.003
	Zn	0.05 (0.10)	0.096 ± 0.005	0.099 ± 0.011	0.087 ± 0.009
	Mo	0.15	0.17 ± 0.011	0.16 ± 0.008	0.18 ± 0.032
Initial concentration in $BG11_{NO3}$	B	0.50	0.507 ± 0.02	0.544 ± 0.03	0.512 ± 0.05
	Na	414.62	541.976 ± 48.15	451.864 ± 18.32	446.314 ± 10.2
	Mg	7.40	8.368 ± 0.53	7.99 ± 0.64	8.142 ± 0.58
	P	7.11 (8.50)	8.351 ± 0.24	8.351 ± 0.81	8.623 ± 0.76
	K	17.96	23.834 ± 1.28	21.487 ± 0.8	21.219 ± 0.86
	Ca	9.81	10.461 ± 0.5	10.191 ± 0.54	9.844 ± 0.65
	Mn	0.50	0.519 ± 0.11	0.52 ± 0.09	0.5 ± 0.11
	Fe	1.28	0.331 ± 0.01	0.95 ± 0.17	5.274 ± 0.53
	Co	0.01	0.009 ± 0.001	0.009 ± 0.002	0.009 ± 0.002
	Ni	0.00 (0.01)	0.009 ± 0.003	0.01 ± 0.003	0.01 ± 0.004
	Cu	0.02	0.018 ± 0.002	0.017 ± 0.002	0.019 ± 0.003
	Zn	0.05 (0.10)	0.091 ± 0.001	0.085 ± 0.004	0.094 ± 0.012
	Mo	0.15	0.129 ± 0.019	0.13 ± 0.019	0.141 ± 0.012
Initial concentration in $BG11_{urea}$	B	0.50	0.596 ± 0.16	0.611 ± 0.15	0.585 ± 0.15
	Na	8.87	154.41 ± 18.11	153.238 ± 15.76	157.381 ± 21.34
	Mg	7.40	7.361 ± 0.26	7.378 ± 0.3	7.317 ± 0.27
	P	7.11 (8.50)	8.745 ± 0.73	8.535 ± 0.61	8.619 ± 0.76
	K	17.96	17.135 ± 0.84	17.122 ± 1	17.443 ± 0.79
	Ca	9.81	8.23 ± 0.92	7.285 ± 0.97	7.553 ± 1.1
	Mn	0.50	0.539 ± 0.08	0.528 ± 0.09	0.48 ± 0.13
	Fe	1.28	0.264 ± 0.02	1.193 ± 0.14	5.919 ± 0.59
	Co	0.01	0.008 ± 0.001	0.007 ± 0.002	0.008 ± 0.002
	Ni	0.00 (0.01)	0.009 ± 0.006	0.008 ± 0.004	0.005 ± 0.004
	Cu	0.02	0.017 ± 0.004	0.02 ± 0.004	0.02 ± 0.001
	Zn	0.05 (0.10)	0.088 ± 0.004	0.09 ± 0.003	0.079 ± 0.007
	Mo	0.15	0.137 ± 0.01	0.154 ± 0.01	0.145 ± 0.01

Equations for biomass and cell density estimation

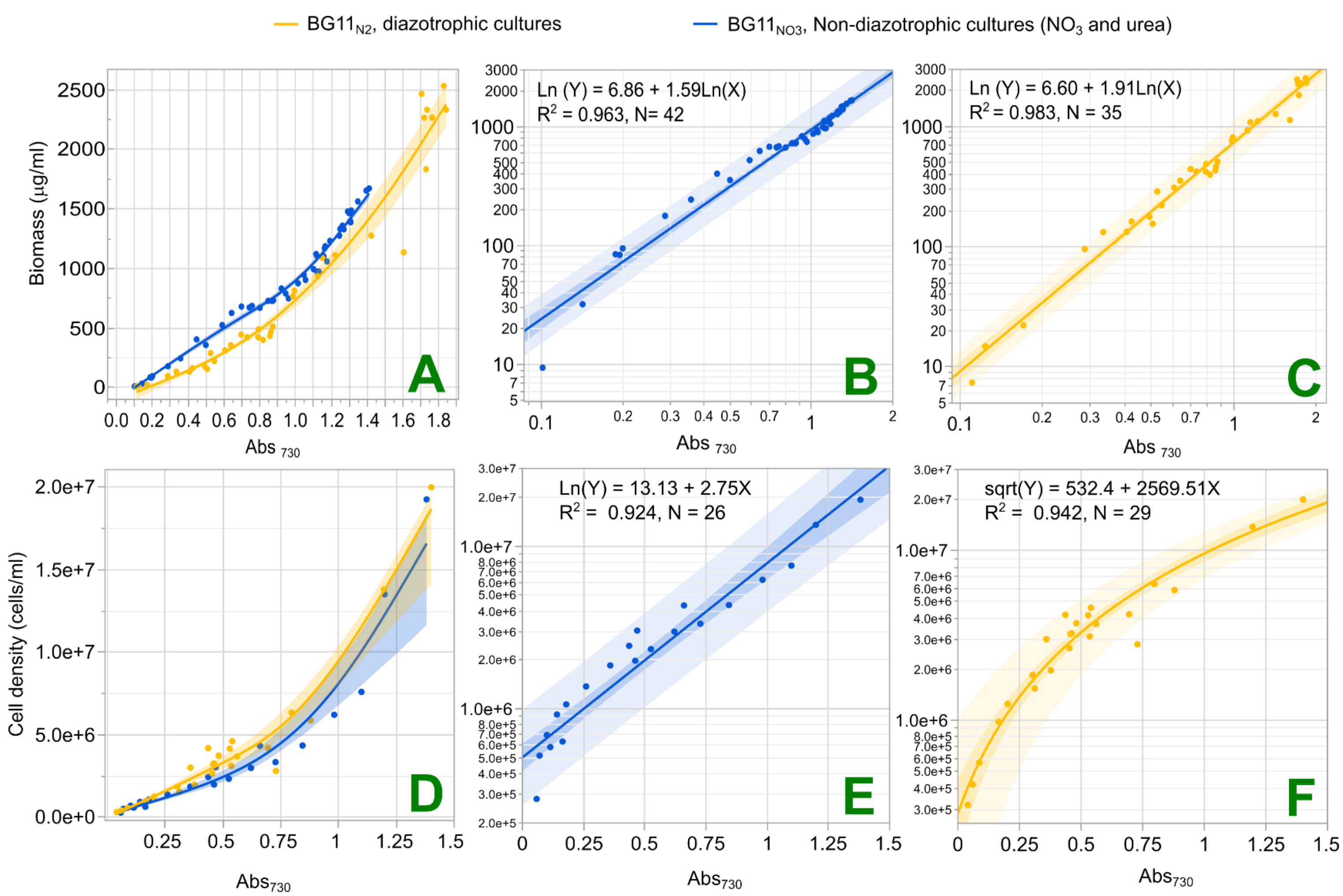

Figure S2. Correlation for estimating *Anabaena* biomass concentration and cell density from apparent absorbance

A: Biomass concentration is proportional to culture apparent absorbance @ 730 nm (Abs_{730}). The relationship is similar for diazotrophic and non-diazotrophic cultures, but not the same. B: Prediction model for biomass of Non-diazotrophic cultures (N=42 pairs). C: Prediction model for biomass of diazotrophic cultures (N=35 pairs). D: Cell density is proportional to culture Abs_{730}. The relationship is similar for diazotrophic and non-diazotrophic cultures, but not the same. E: Prediction model for cell density of Non-diazotrophic cultures (N=26 pairs). F: Prediction model for cell density of diazotrophic cultures (N=29 pairs). Shaded areas represent the confidence interval of the predicted values, with significance level α=0.05.

Saturation kinetic model and culture appearance

The saturation kinetic model (Eq. 1) involves two parameters: the intrinsic growth rate constant (μ_{max}, day $^{-1}$), and the normalized ratio between final and initial populations (y_∞). Since bacterial reproduction implies bipartition, the experimental growth rate was calculated as μ_{max}/Ln(2) and generation times were calculated as Ln(2)/μ_{max}

$$\mathrm{Ln}\left(\frac{N}{N_o}\right) = \mathrm{Ln}\left(\frac{Abs_{730}}{Abs_{730_{t=0}}}\right) = y_\infty \cdot \left(1 - \exp\left(\frac{-\mu_{max} \cdot t}{y_\infty}\right)\right) \quad (1)$$

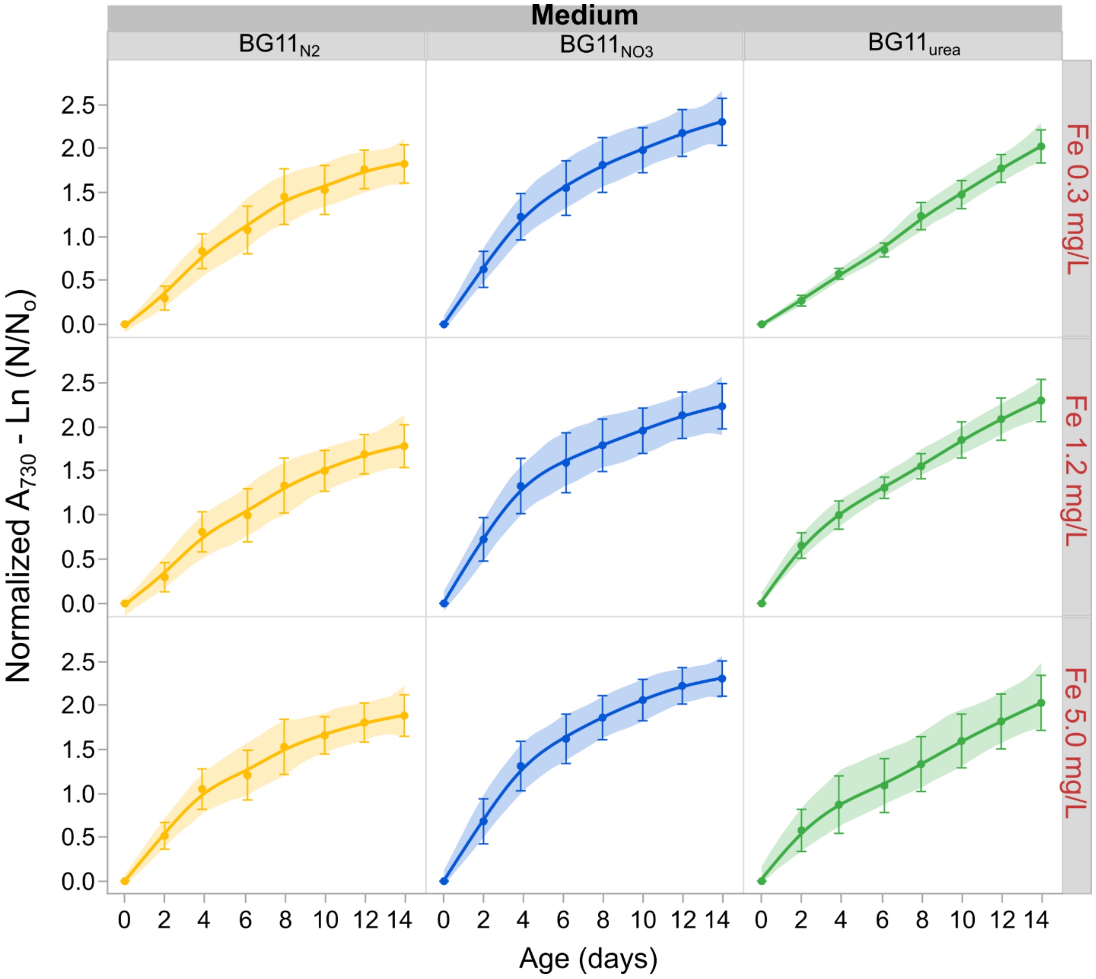

Figure S3. Normalized growth data with saturation kinetic model

Abs_{730} readings were normalized to have a clearer view of exponential and stationary phases. The term Ln (N/N_o) represents the natural logarithm of the ratio between Abs730 measurements over time and the starting Abs_{730} (normally 0.1). Shaded areas represent the confidence interval of mean values, with significance level α=0.05. Error bars are constructed using one standard error from the mean. Data summarizes information from three biological replicates for each combination of medium and Fe concentration, for a total of 27 cultures. $BG11_{urea}$ cultures presented longer exponential phase.

Figure S4 summarizes growth parameters obtained from adjusting growth data in different media to the saturation kinetic model. Figure S5 illustrates the differences in pigmentation of *Anabaena* cells cultured in $BG11_{N2}$, $BG11_{NO3}$, and $BG11_{urea}$.

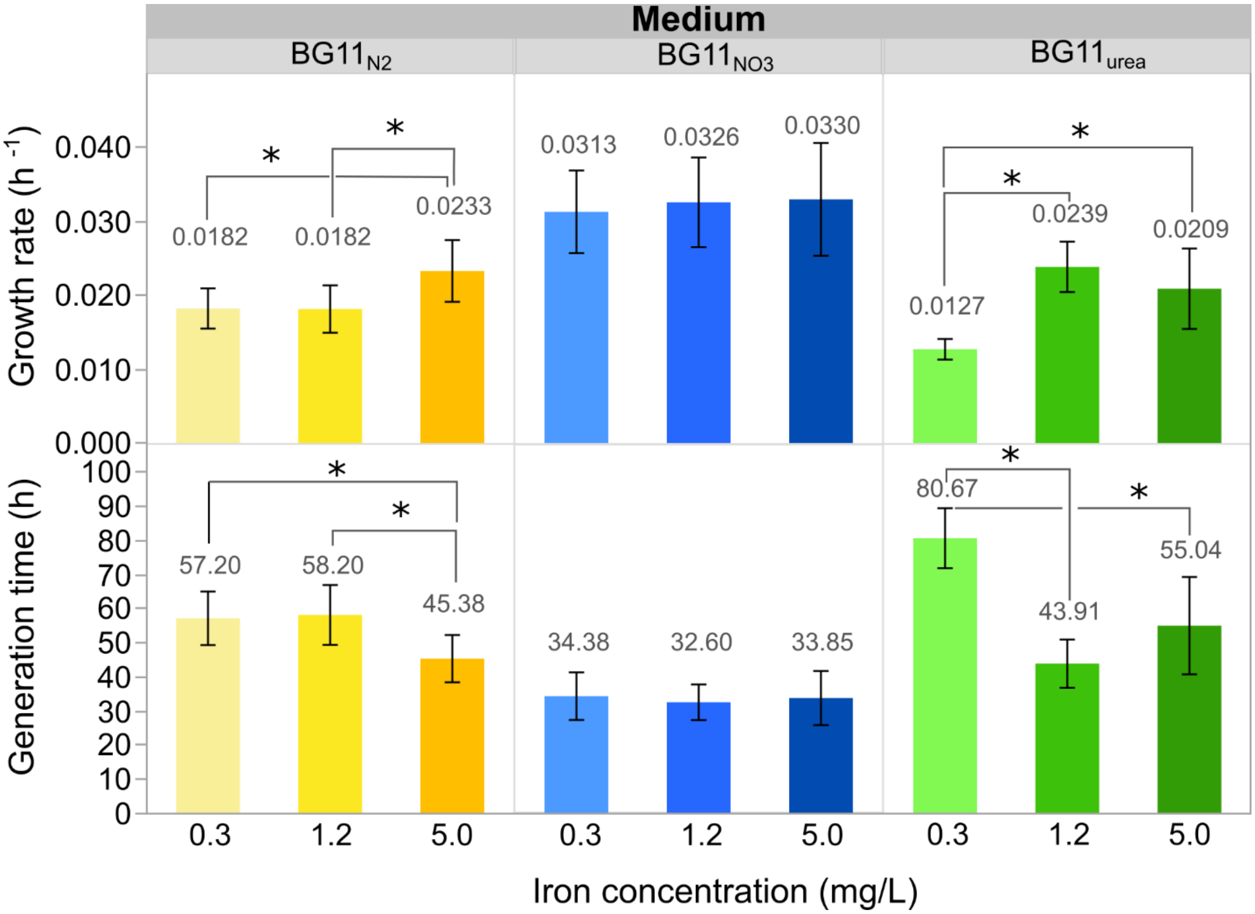

Figure S4. Growth rates and generation times of *Anabaena* in different media

Varying initial Fe concentrations affect growth rates significantly in $BG11_{N2}$ and $BG11_{urea}$ media. Growth rates and generation times are also affected by the N-source. Fastest growth rates and shortest generation times correspond to cultures in $BG11_{NO3}$ medium. Cultures in $BG11_{N2}$ and $BG11_{urea}$ presented comparable growth rates but low initial Fe concentration reduces growth speed. Bar heights represent the average of three independent replicates. Error bars are constructed considering one standard error from the mean. Stars represent statistically significant differences among treatments with a significance level α=0.05. Lighter color shades represent lower Fe-levels.

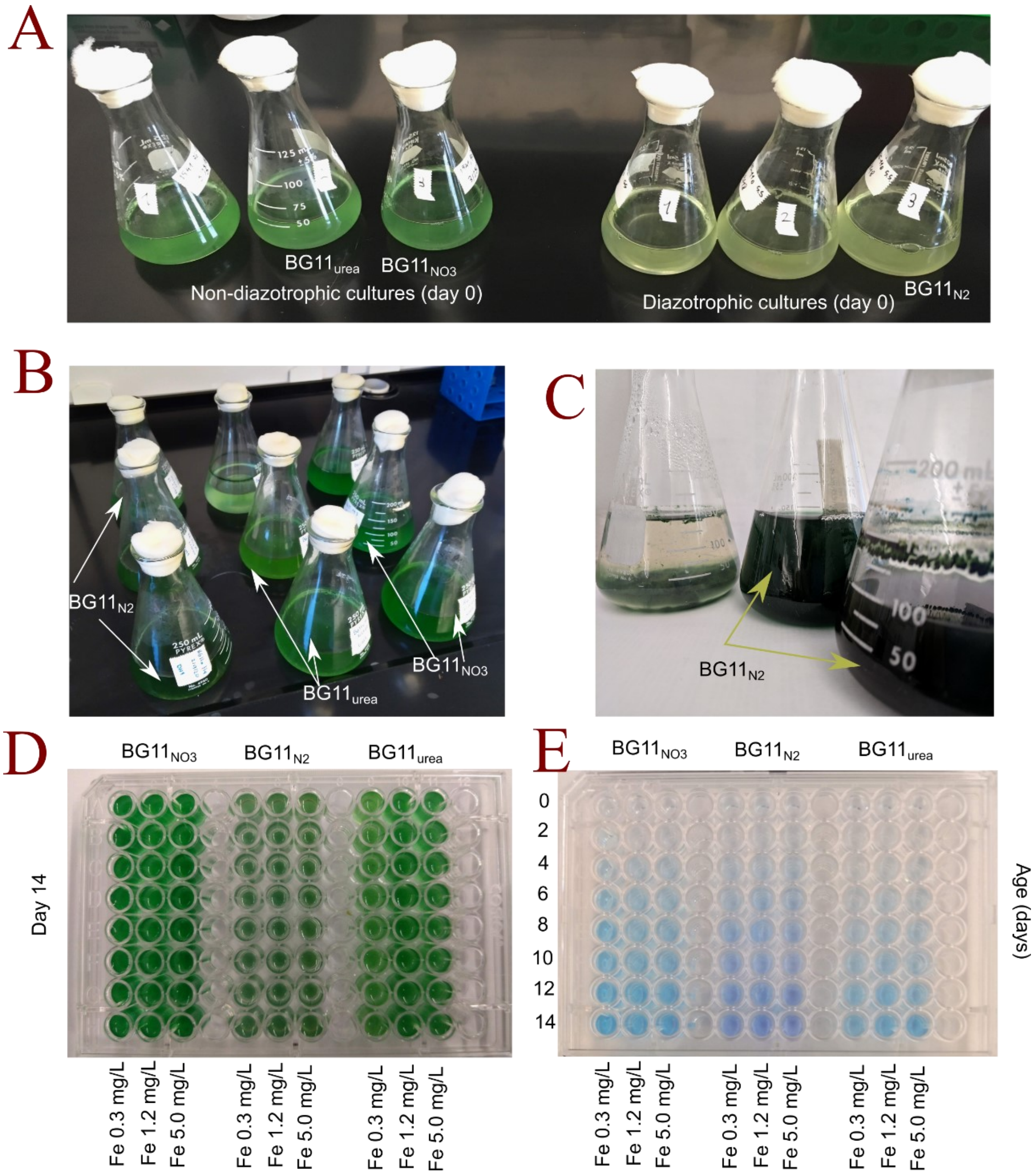

Figure S5. Pigmentation of *Anabaena* cultures

A: Difference in pigmentation between diazotrophic and non-diazotrophic *Anabaena* at the beginning of growth experiments (Abs_{730} = ~0.2). B: Difference in pigmentation among Anabaena cultures in $BG11_{N2}$, $BG11_{urea}$, and $BG11_{NO3}$ (Abs_{730} = ~0.4). C: Appearance of Anabaena cultures in $BG11_{N2}$ (Abs_{730} = ~1.2). Diazotrophic cultures are darker and clumpier. D: Microplate culture appearance at day 14. Healthy $BG11_{NO3}$ and $BG11_{urea}$ cultures are also brighter than $BG11_{N2}$ cultures. E: Progressive accumulation of PBPs. The differences in cellular pigmentation are evident between diazotrophic ($BG11_{N2}$) and non-diazotrophic cultures ($BG11_{NO3}$ and $BG11_{urea}$). Increased content of PEC (purple) in $BG11_{N2}$ cells grants darker pigmentation. Production of PBPs is faster for cells grown in $BG11_{NO3}$ and $BG11_{N2}$ media. Fe levels have a more significant effect on the production of PBPs in $BG11_{urea}$.

Production of PBPs in different N-sources

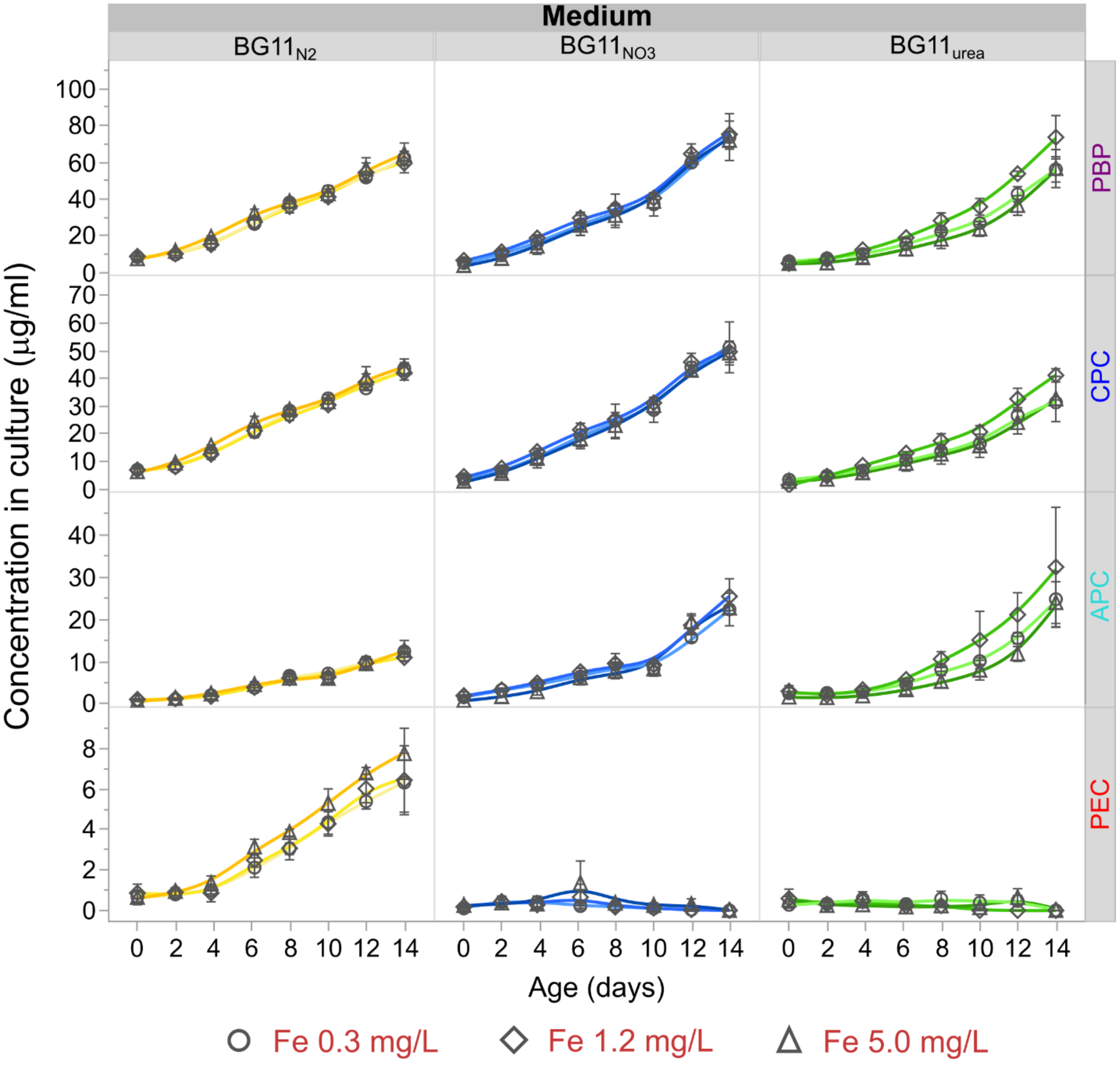

Figure S6. Concentration of PBPs in media with different N-sources

While Phycoerythrocyanin (PEC) production was significantly higher in $BG11_{N2}$ cultures, C-phycocyanin (CPC) and Allophycocyanin (APC) production profiles were more pronounced in $BG11_{NO3}$ and $BG11_{urea}$ cultures, respectively. *Anabaena* cells in $BG11_{NO3}$ and $BG11_{urea}$ produced comparable profiles of PBPs, but pigment-protein production was slower in $BG11_{urea}$ cultures. Markers represent the average of three biological replicates and error bars are constructed considering one standard error from the mean. Lighter color shades represent lower Fe-levels.

Table S5. PBPs abundance per cell in different growth media

Values represent the average and standard deviation from three independent biological replicates per Medium – Fe level treatment. Two-way ANOVA was used to analyze statistically significant differences with a significance level α=0.05. Medium type and Fe-level used as independent predictors. Cellular abundance data correspond to PBPs accumulated on day 8 only. Medium levels not connected by same group letter are significantly different. Fe-levels not connected by same number of stars within medium type are significantly different. PEC (Medium p-Value < 0.0001; Fe-level p-Value = 0.7619; interaction p-Value = 0.4833), CPC (Medium p-Value = 0.0095; Fe-level p-Value = 0.0516; interaction p-Value = 0.8768), APC (Medium p-Value < 0.0001; Fe-level p-Value = 0.0006; interaction p-Value = 0.03415), PBP (Medium p-Value = 0.0012; Fe-level p-Value = 0.0098; Interaction p-Value = 0.4817)

Medium	PEC (pg/cell)	Group	CPC (pg/cell)	Group	APC (pg/cell)	Group	Total PBP (pg/cell)	Group
**a.BG11$_{N2}$**	**1.26 ± 0.24**	**A**	**10.81 ± 2.04**	**A**	**2.39 ± 0.35**	**A**	**14.46 ± 2.47**	**A**
Fe 0.3 ppm	1.2 ± 0.1	*	11.3 ± 1.72	*	2.59 ± 0.08	*	15.09 ± 1.87	*
Fe 1.2 ppm	1.31 ± 0.35	*	11.63 ± 2.23	*	2.58 ± 0.17	*	15.52 ± 2.62	*
Fe 5.0 ppm	1.27 ± 0.3	*	9.49 ± 2.17	**	1.99 ± 0.32	**	12.76 ± 2.74	**
**b.BG11$_{NO3}$**	**0.09 ± 0.1**	**B**	**8.02 ± 1.47**	**B**	**2.92 ± 0.66**	**A**	**11.03 ± 2.09**	**B**
Fe 0.3 ppm	0.08 ± 0.13	*	8.2 ± 0.7	*	3.08 ± 0.39	*	11.36 ± 0.56	*
Fe 1.2 ppm	0.07 ± 0.11	*	8.7 ± 1.81	*	3.3 ± 0.65	*	12.07 ± 2.53	*
Fe 5.0 ppm	0.13 ± 0.08	*	7.16 ± 1.75	**	2.38 ± 0.7	**	9.67 ± 2.51	**
**c.BG11$_{urea}$**	**0.26 ± 0.32**	**B**	**10.12 ± 1.97**	**A**	**5.56 ± 1.8**	**B**	**15.94 ± 3.71**	**A**
Fe 0.3 ppm	0.48 ± 0.53	*	11.49 ± 1.7	*	6.77 ± 0.61	*	18.74 ± 2.09	*
Fe 1.2 ppm	0.14 ± 0.08	*	10.39 ± 1.24	*	6.36 ± 1.54	*	16.88 ± 2.72	*
Fe 5.0 ppm	0.15 ± 0.06	*	8.49 ± 2.03	**	3.56 ± 1.06	**	12.2 ± 3.03	**

Extraction methods for Chl*a* and CaroT

Two different methods of solvent extraction were used to measure the concentration of Chl*a* and carotenoids in liquid cultures of *Anabaena*. In the first approach, solvent extraction with cold acetone 90% v/v was used to have an indicator of the concentration of Chl*a* and total carotenoids (CaroT) every other day. Since acetone is a less effective solvent for extracting these hydrophobic pigments [38–40], measured concentrations of these compounds are significantly lower than the actual abundances. However, these data were used as an indicator of the relative abundance of hydrophobic pigments in different growth media. In the second method, cold extraction with Methanol was used for precise measurement of β-Carotene and other hydrophobic pigments. Specific details about acetone and methanol extraction are described next.

Cold Acetone Extraction

Samples of *Anabaena* cultures (1.5 mL) were centrifuged for 15 minutes at 15000 RCF to isolate the cells from their medium. The supernatant was separated from the cell pellet and replaced by 1 mL of 90% v/v acetone. The mixture was homogenized for 10 seconds at 40 W with a sonic dismembrator and stored in darkness at 4°C for 4 hours. The resulting yellow extract was separated from spent cells by a second round of centrifugation at 15000 RCF for 15 minutes.

Cold Methanol Extraction

The extraction method was like the cold acetone extraction process, but the supernatant from the first centrifugal separation was replaced by 1.5 mL of pure cold methanol in 1.5-mL microcentrifuge tubes. For highest quantification accuracy and lowest possible carotenoid degradation, the presence of air in the tubes was kept at the minimum by avoiding headspace. After sonic homogenization, samples were extracted in darkness at 4°C for 2 hours. Extraction samples were always kept cold, either on ice or at 4°C, to minimize degradation. Carotene extracts from lyophilized pellets of *Anabaena* did not present apparent differences in concentration, compared to extracts from pellets that were not subject to freeze drying. Final Methanol extracts were obtained by separating spent cells via centrifugation (15 minutes at 15000 RCF and 4°C).

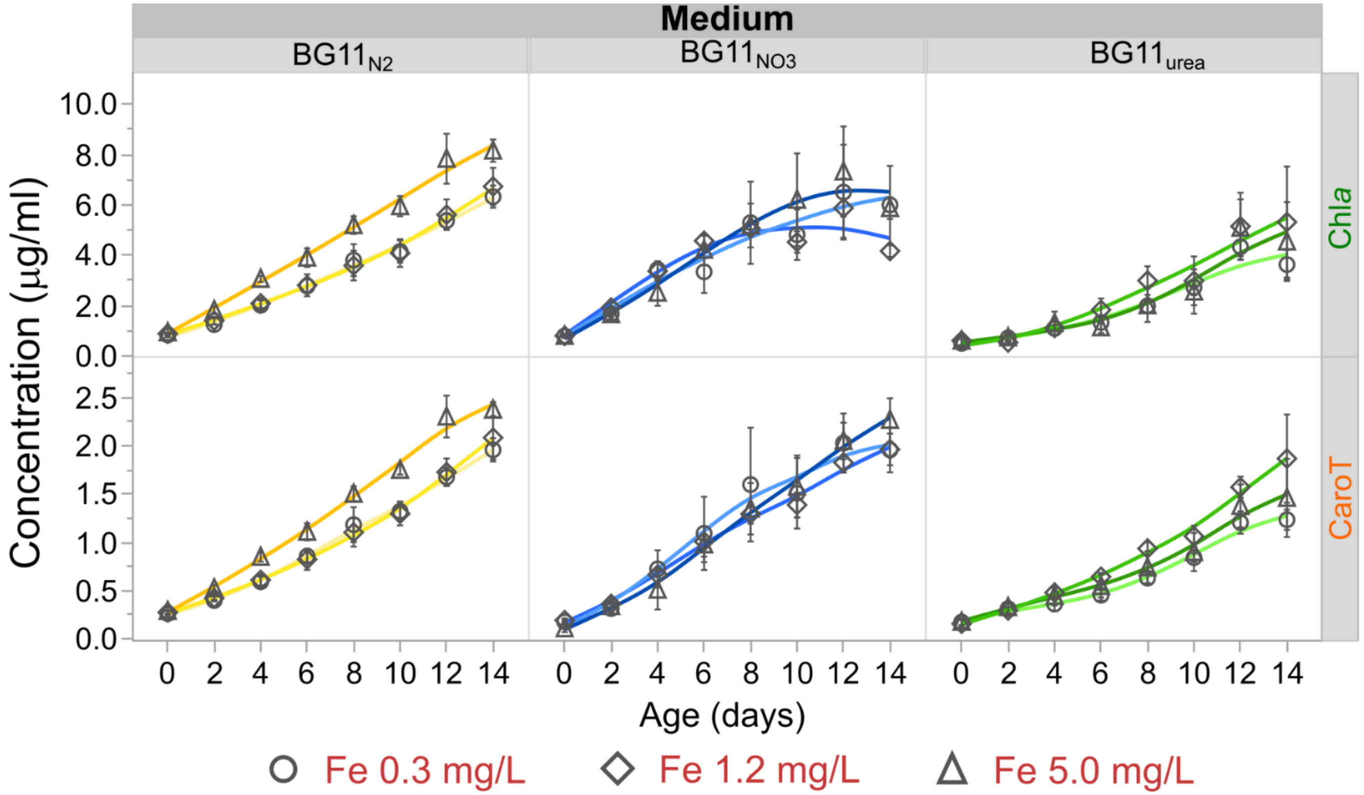

Figure S7. Concentration of Chl*a* and CaroT in different growth media

Anabaena cells in all media presented steady increase in concentration of hydrophobic pigments (Chl*a* and CaroT). Highest Chl*a* and CaroT production at 14 days was achieved in $BG11_{N2}$ medium with Fe 5.0 mg/L. Markers represent the average of three biological replicates and error bars are constructed considering one standard error from the mean. Lighter color shades represent lower Fe-levels.

Table S6. Chl*a*, CaroT, and β-Carotene abundance per cell in different growth media

Values represent the average and standard deviation from three independent biological replicates per Medium – Fe level treatment. Two-way ANOVA was used to analyze statistically significant differences with a significance level α=0.05. Medium type and Fe-level used as independent predictors. Cellular abundance data correspond to Chl*a*, CaroT and β-Carotene accumulated on day 6 only. Medium levels not connected by same group letter are significantly different. Fe-levels not connected by same number of stars within medium type are significantly different. Chl*a* (Medium p-Value = 0.0084; Fe-level p-Value = 0.5757; interaction p-Value = 0.4018), CaroT (Medium p-Value = 0.3131. Fe-level p-Value = 0.8196; interaction p-Value = 0.9257), β-Carotene (Medium p-Value < 0.0001; Fe-level p-Value = 0.8377; interaction p-Value < 0.0001)

Medium	Chl*a* (pg/cell)	Group	CaroT (pg/cell)	Group	β-Carotene (pg/cell)	Group
**a.BG11$_{N2}$**	**1.83 ± 0.22**	**A**	**0.54 ± 0.05**	**A**	**2.41 ± 0.56**	**A**
Fe 0.3 ppm	1.77 ± 0.32	*	0.55 ± 0.07	*	2.47 ± 0.24	*
Fe 1.2 ppm	1.79 ± 0.2	*	0.53 ± 0.06	*	3 ± 0.29	*
Fe 5.0 ppm	1.94 ± 0.12	*	0.55 ± 0.03	*	1.77 ± 0.1	**
**b.BG11$_{NO3}$**	**1.94 ± 0.45**	**A**	**0.48 ± 0.1**	**A**	**3.84 ± 0.67**	**B**
Fe 0.3 ppm	1.66 ± 0.18	*	0.52 ± 0.14	*	3.54 ± 0.07	*
Fe 1.2 ppm	2.15 ± 0.55	**	0.46 ± 0.1	*	3.38 ± 0.15	*
Fe 5.0 ppm	2 ± 0.54	**	0.45 ± 0.11	*	4.59 ± 0.68	**
**c.BG11$_{urea}$**	**1.31 ± 0.48**	**B**	**0.49 ± 0.09**	**A**	**1.17 ± 0.57**	**C**
Fe 0.3 ppm	1.45 ± 0.15	*	0.49 ± 0.1	*	1.29 ± 0.65	*
Fe 1.2 ppm	1.48 ± 0.79	*	0.51 ± 0.07	*	1.18 ± 0.65	*
Fe 5.0 ppm	0.99 ± 0.18	*	0.48 ± 0.12	*	1.05 ± 0.48	*

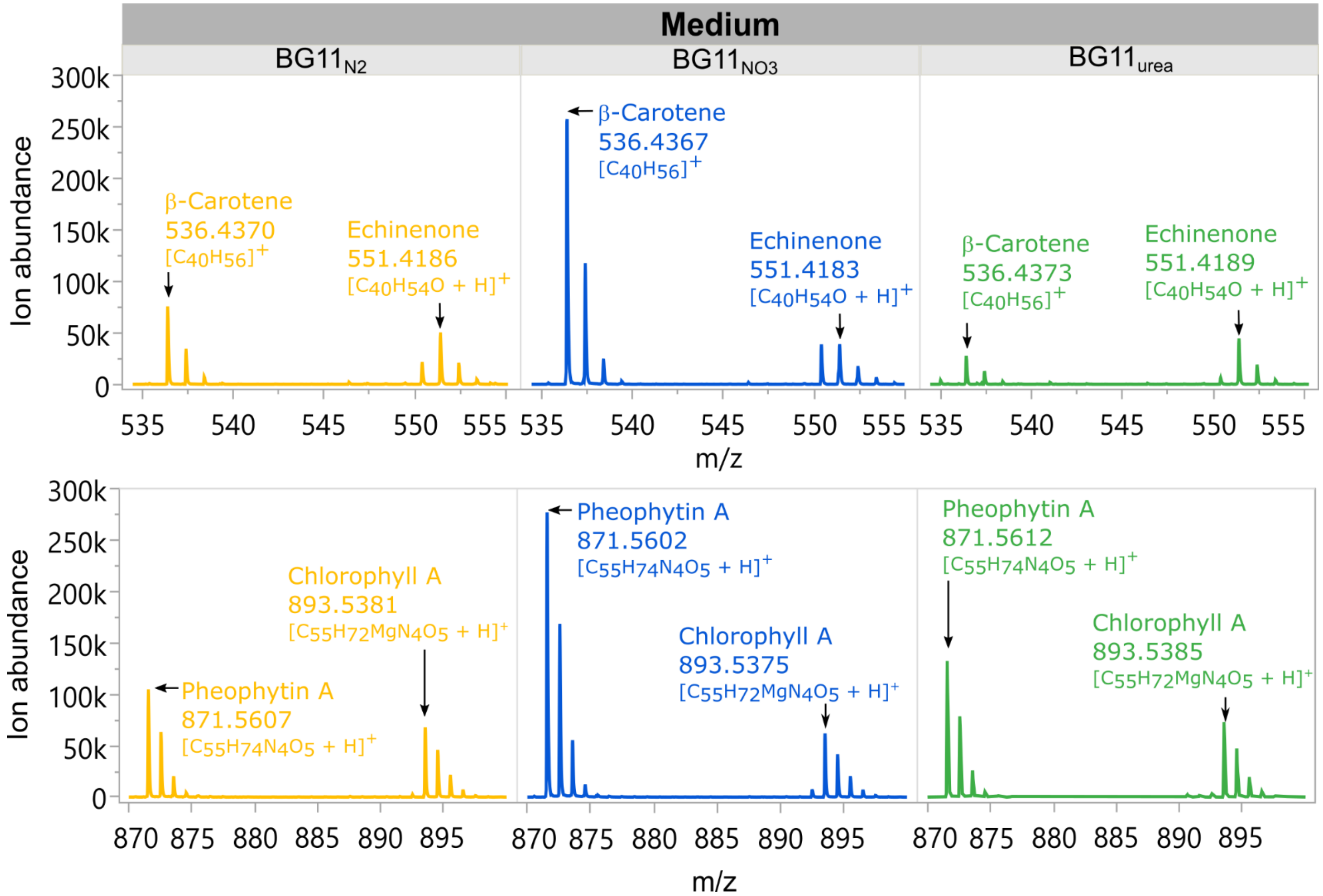

Figure S8. MS-TOF analysis of methanol extracts

MS was used to identify β-Carotene, Echinenone, Pheophytin *a* and Chl*a* in methanol extracts of *Anabaena* based on their representative masses. Peak heights indicate abundance of the corresponding ions. Extracts from $BG11_{N2}$, $BG11_{NO3}$ and $BG11_{urea}$ cultures with similar levels of Chl*a* are compared.

Methods for estimation of TOC and N-sources consumption

TOC was measured with a HACH high range total organic Carbon reagent set (product #2760445). Manufacturer's instructions were followed to measure TOC in a HACH DR 6000 spectrophotometer (Program 426). Standards with 200 and 500 mg/L of organic Carbon were used for quality control. Blanks were prepared with organic-carbon free HPLC grade water. Samples of cells grown in $BG11_{urea}$ medium were diluted four times before TOC analysis. Samples from $BG11_{NO3}$ and $BG11_{N2}$ cultures did not require prior dilution.

Nitrate concentration was measured on supernatants resulting from centrifuging 1-mL samples of $BG11_{NO3}$ cultures. 100-µL aliquots of supernatant were diluted with 9.9 mL of DI water (100 times dilution). Nitrate was determined with the Cadmium reduction method using NitraVer 5 Nitrate Reagent powder pillows [41] and a DR300 Pocket colorimeter (HACH# LPV445.97.02110), following manufacturer's instructions. Urea concentration in cell-free liquid was quantified with the O-phthalaldehyde (OPA) reaction with Naphtylethylenediamine (NED) [42]. Briefly, 500 µl of OPA reagent were mixed with 25 µl of sample and 250 µl of deionized water and thoroughly mixed. Then, 500 µl of NED reagent were added to the mixture. The liquid samples were placed in a water bath at 37°C for 4 minutes and the absorbance at 480 nm was immediately measured. A reagent blank prepared with DI water instead of sample was used as blank. The urea calibration curve is presented in Figure S17.

N_T content of diazotrophic cultures in $BG11_{N2}$ medium was measured by adapting the HACH Test 'N tube persulfate digestion method [43]. This method determined global N_T concentration in each $BG11_{N2}$ culture. Change on global N_T concentration over time was only possible if any N-source was entering the system during the experiments (principle of mass conservation). Briefly, persulfate digestion reagent was prepared in DI water with potassium persulfate ($K_2S_2O_8$, 50 mg/mL), NaOH (16.8 mg/mL) and boric acid (H_3BO_3, 30 mg/mL). For digestion, 1 mL of persulfate reagent was mixed with 1 mL of diazotrophic culture sample in 3 -mL glass vials. Culture samples were directly taken from well-shaken $BG11_{N2}$ cultures, so that both cells and growth medium were digested. The vials were placed inside an autoclave at 121°C for 1 hour to oxidize the organic matter and transform all N-containing compounds in the culture suspension into NO_3. The NO_3 concentration of the digested $BG11_{N2}$ samples was quantified with the Cadmium reduction method (NitraVer 5 Nitrate Reagent) in a DR300 Pocket colorimeter as previously described. NO_3 concentrations were converted to N_2 equivalents using reaction stoichiometry. 10 mM glycine and serine standards were oxidized with the adapted persulfate digestion method to determine the oxidation efficiency of the process. The minimum conversion efficiency of glycine and serine into nitrates after persulfate digestion was 96%. Hence, the stoichiometric transformation to N_2 (and N_T equivalents) did not involve correction factors. Over-time change on N_T content of $BG11_{N2}$

cultures was used as an indicative of N_2-fixation because this growth medium only contained negligible initial concentration of elemental N coming from Fe and trace element sources (See Table S3). No additional nutrients were added as the experiments progressed. Therefore, the only explanation to increasing concentration in global N_T was fixation of N_2 from the atmosphere surrounding the culture. Note that the foam plugs covering the flasks allowed for gas exchange. Even with the initial small quantities of ammonium ferric citrate in $BG11_{N2}$ cultures, these do not contribute to the estimated N_2-fixation rate because the slopes of the straight lines in Figure S9B are not affected by the initial concentration of N-source (y-axis intercept).

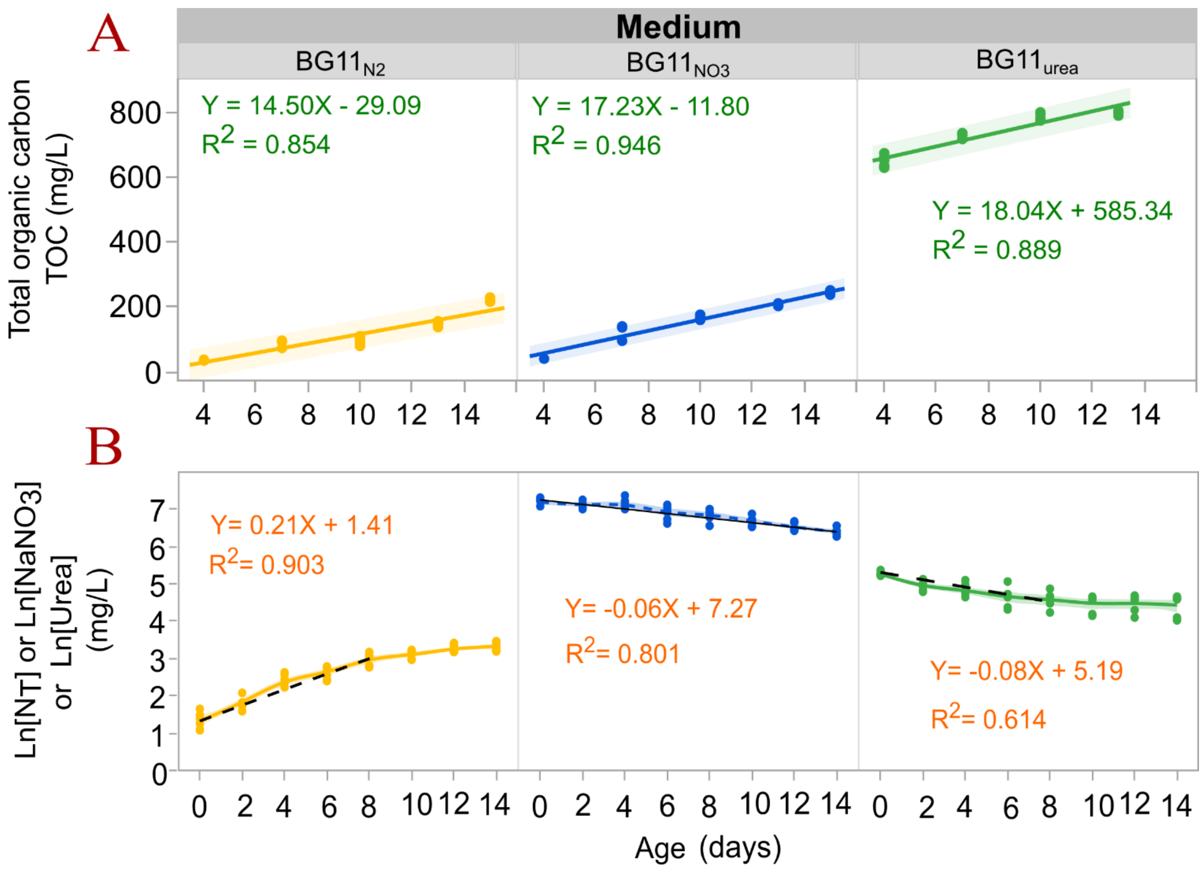

Figure S9. Consumption rates of CO_2 and N-sources

A: TOC formation profiles; $BG11_{urea}$ medium presented higher initial content of organic carbon due to the TES buffer. TOC profiles were fitted to a linear model to determine zero-order CO_2 fixation rates in each medium. Data represent four biological replicates per medium type. Shaded areas represent confidence fit intervals using a significance level α=0.05. **B:** N-source consumption profiles (N_2, $NaNO_3$ and urea) were adjusted to a first order kinetic model. Data represent the mean of six biological replicates per medium type. Rate constants for N_2-fixation ($BG11_{N2}$) and urea consumption ($BG11_{urea}$) were calculated with data from the exponential phase (up to day 8). NO_3 was continuously consumed for two weeks in $BG11_{NO3}$

Urease activity analysis

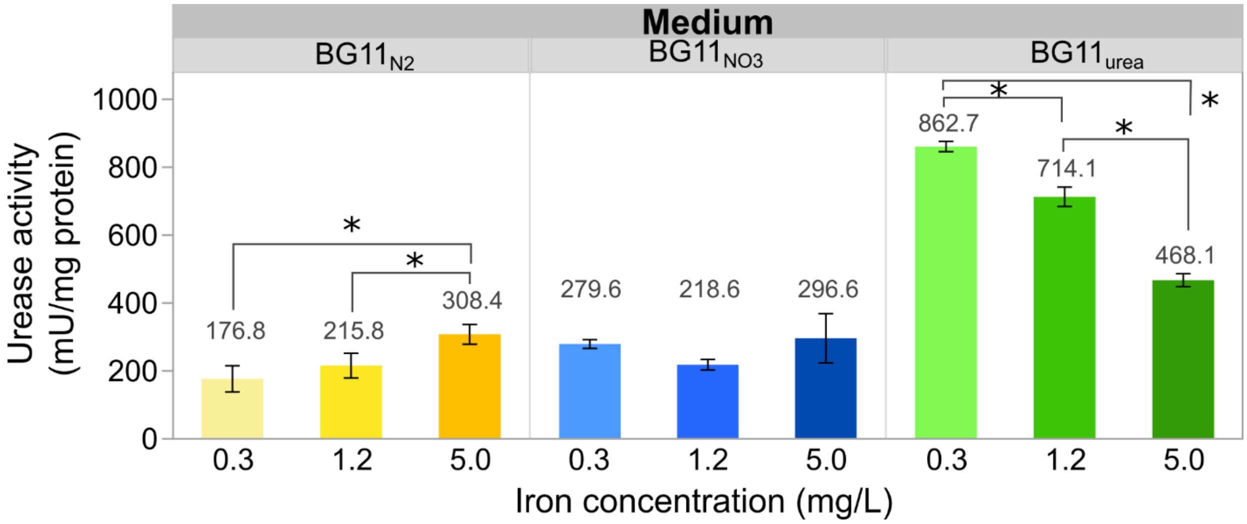

Figure S10. *Anabaena* urease activity in different media

The urease activity of *Anabaena* was highest in $BG11_{urea}$ medium at Fe 0.3 mg/L. Increasing Fe-levels in $BG11_{urea}$ reduced the urease enzyme activity. Higher Fe-levels increased urease activity in $BG11_{N2}$ medium. Bars represent the mean of two biological replicates. Error bars were constructed considering one standard error from the mean. Stars represent statistically significant differences among treatments with a significance level α=0.05. Lighter color shades represent lower Fe-levels.

Consumption profiles of Fe, P, and micronutrients

Concentration of Fe, P, and micronutrient elements in the growth medium was determined by Inductively coupled plasma optical emission spectroscopy (ICP-OES), as presented in the main text of this article.

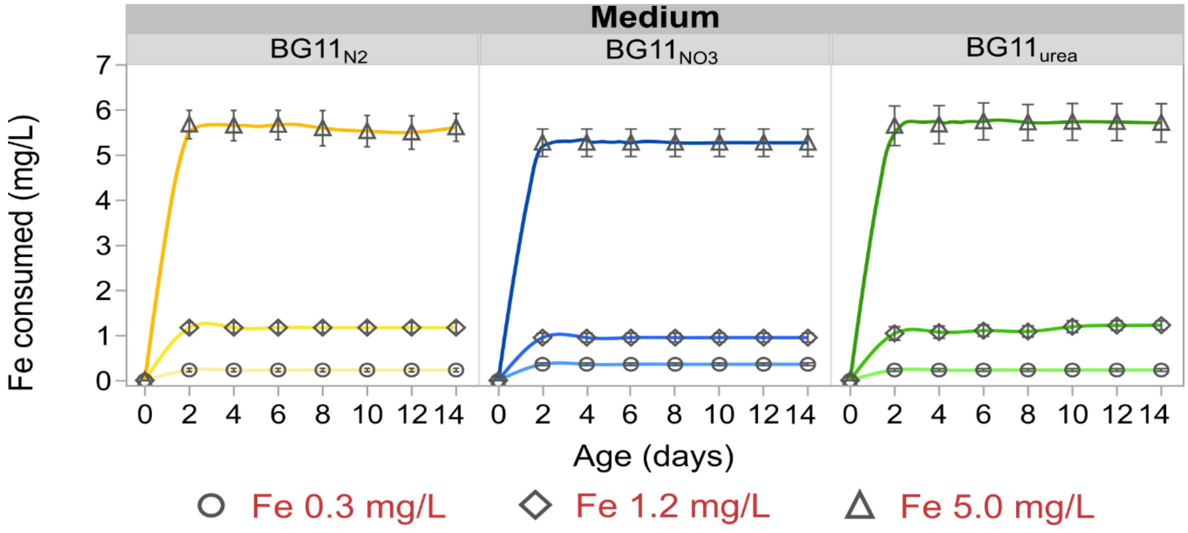

Figure S11. Fe consumption profiles in growth media

Fe was immediately consumed by *Anabaena* cells, regardless of the concentration level supplied in fresh media. Markers represent the average of three biological replicates and error bars are constructed using one standard error from the mean. Lighter color shades represent lower Fe-levels.

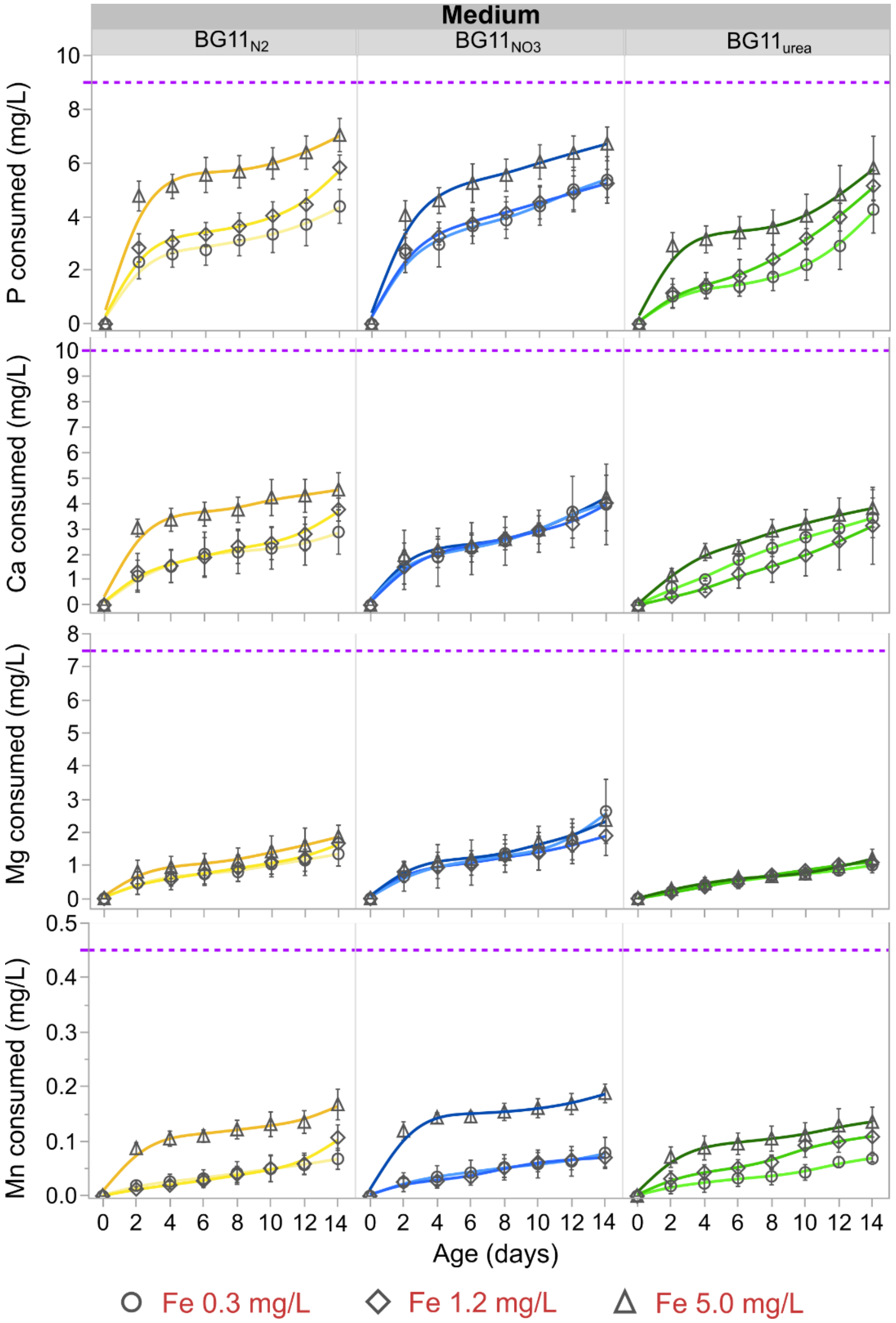

Figure S12. Consumption profiles of P, Ca, Mg and Mn

P, Ca, Mg and Mn were consumed over time. Consumption profiles were affected by starting Fe-levels in the growth medium. Purple dashed lines represent the initial concentration of each element in fresh growth media. P was closest to exhaustion at the end of growth experiments, especially in $BG11_{N2}$ cultures. Markers represent mean values for three biological replicates and error bars are constructed using one standard error from the mean. Lighter color shades represent lower Fe-levels.

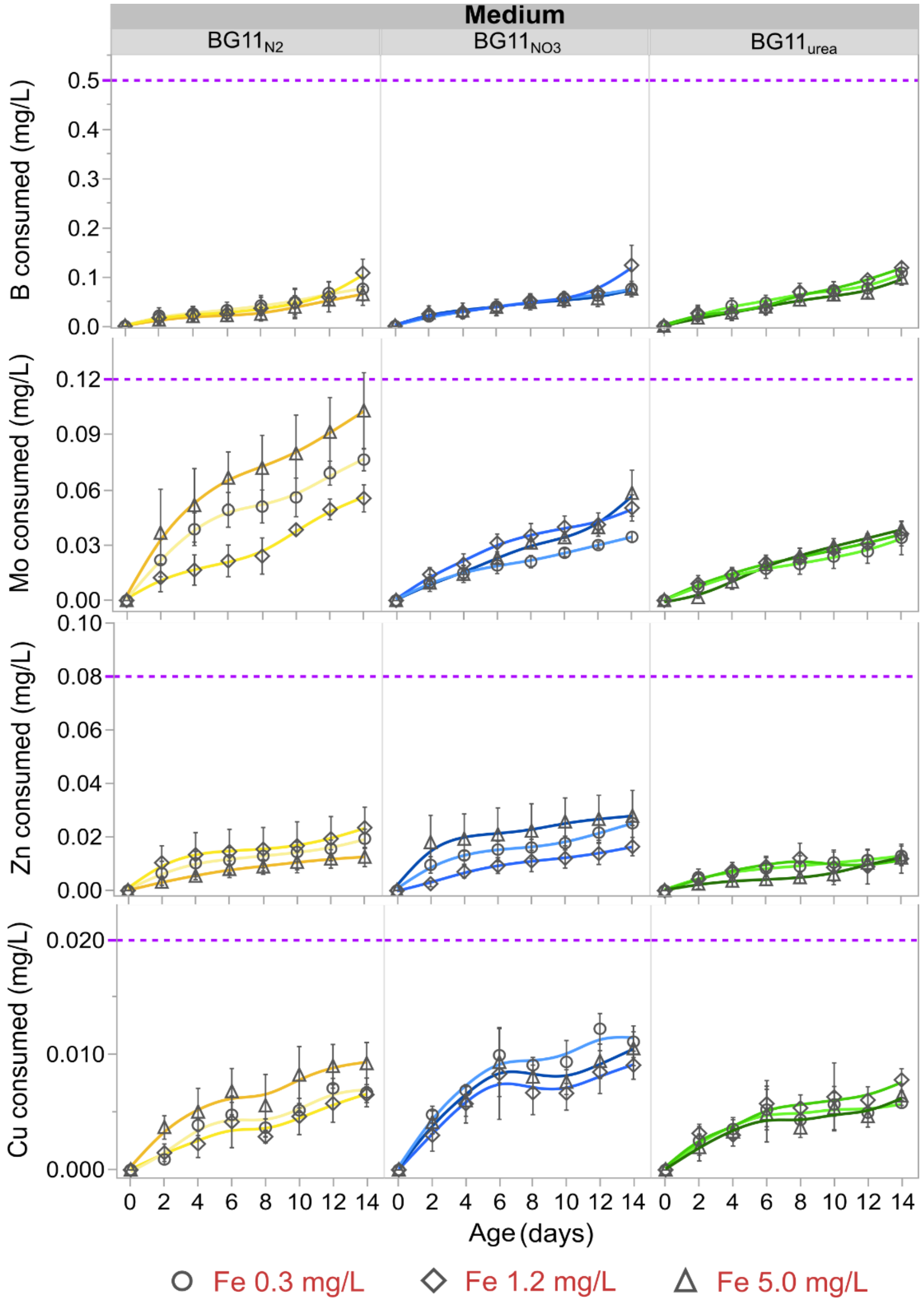

Figure S13. Consumption profiles of B, Mo, Zn and Cu

B, Mo, Zn and Cu were consumed over time. Consumption profiles were affected by starting Fe-levels in the growth medium. Purple dashed lines represent the initial concentration of each element in fresh growth media. Mo was closest to exhaustion at the end of growth experiments, especially in $BG11_{N2}$ cultures. Markers represent mean values for three biological replicates and error bars are constructed using one standard error from the mean. Lighter color shades represent lower Fe-levels.

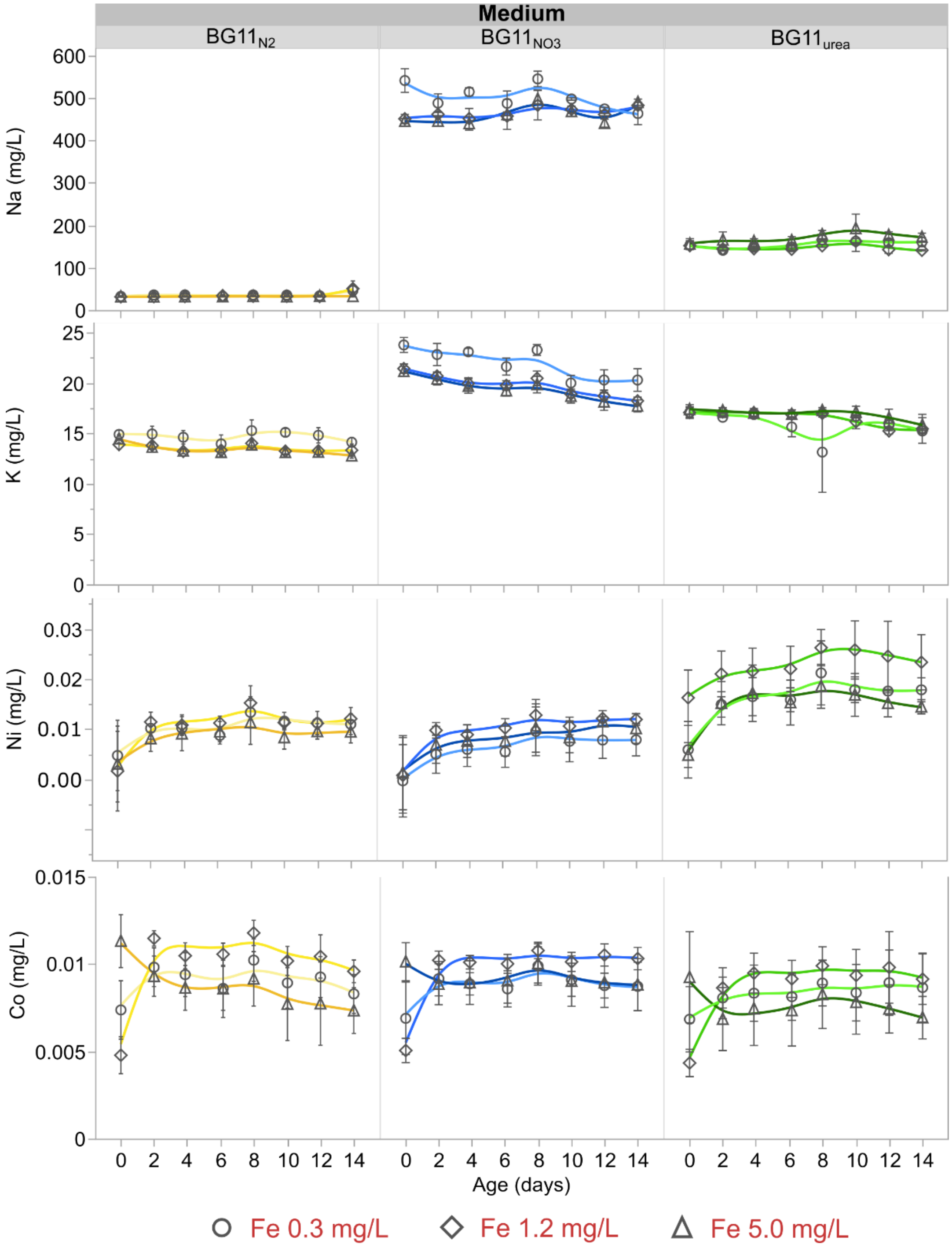

Figure S14. Concentration profiles of Na, K, Ni and Co

Na, K, Ni and Co were not apparently consumed over time. Co levels were very close to the minimum detection level. Markers represent mean values for three biological replicates and error bars are constructed using one standard error from the mean. Lighter color shades represent lower Fe-levels

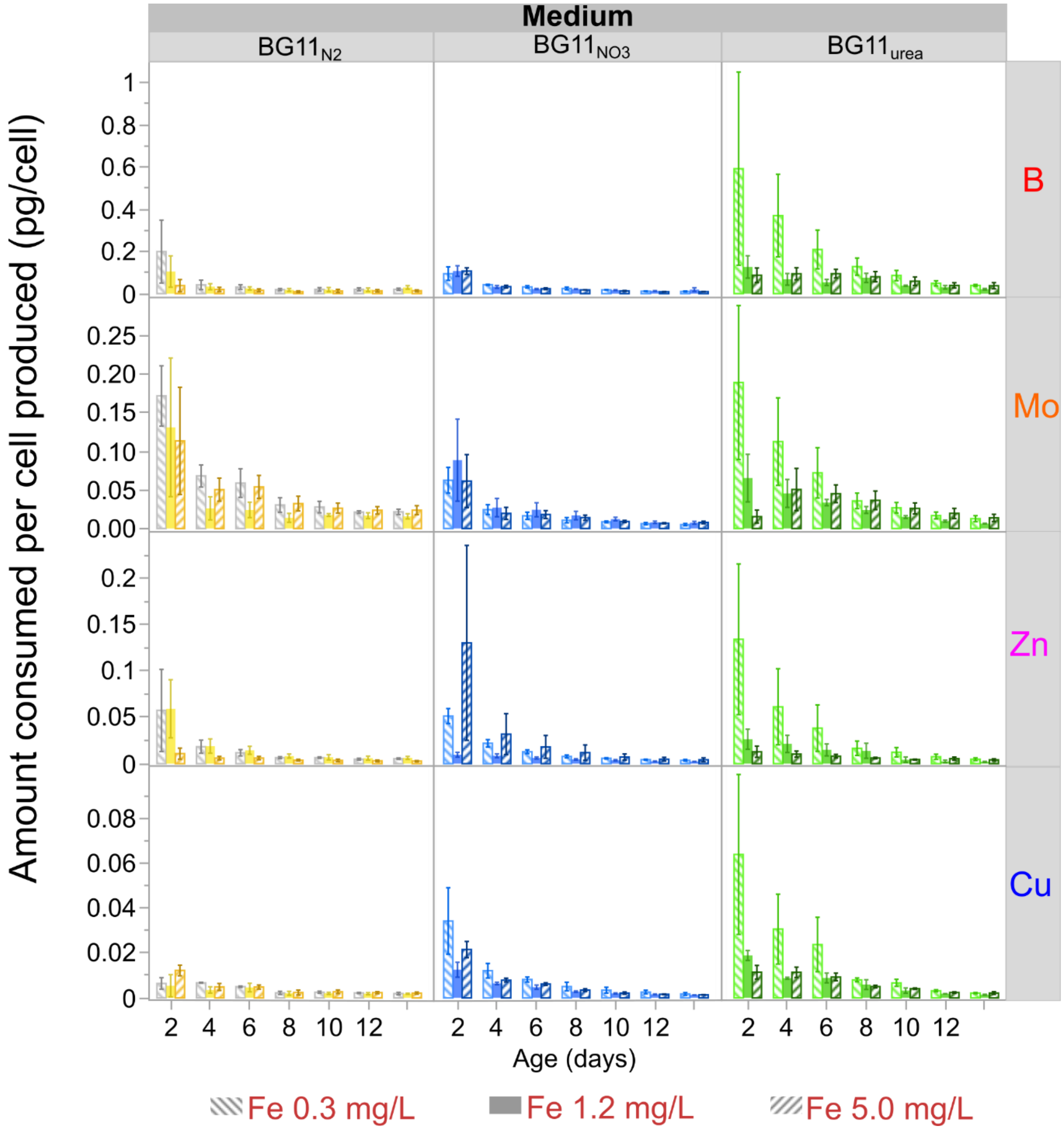

Figure S15. Consumption profiles of B, Mo, Zn and Cu per cell

The demand of these nutrient elements per cell is pronounced during the early stages of cellular growth. High initial Fe-levels maximize the demand of Zn in $BG11_{NO3}$. Low initial Fe-levels increased B, Mo, Zn and Cu consumption in $BG11_{urea}$ medium. Markers represent mean values for three biological replicates and error bars are constructed using one standard error from the mean. Lighter color shades represent lower Fe-levels.

Table S7. P, Ca and Mn requirements per cell produced in different growth media

Values represent the average and standard errors from three independent biological replicates per Medium – Fe level treatment. Multiple linear regression was used to analyze statistically significant differences with a significance level α=0.05. Culture age, medium type and Fe-level used as independent predictors. Cellular abundance data correspond to P, Ca and Mn demands per cell produced on day 4. Medium levels not connected by same group letter are significantly different. Fe-levels not connected by same number of stars within medium type are significantly different. P-demand (Medium p-Value = 0.0692; Fe-level p-Value =0.1970; interaction p-Value = 0.3151), Ca-demand (Medium p-Value = 0.0961; Fe-level p-Value = 0.2411; interaction p-Value = 0.2480), Mn-demand (Medium p-Value =0.0548; Fe-level p-Value = 0.1224; Interaction p-Value = 0.8958)

Medium	P req (pg/cell)	Group	Ca req (pg/cell)	Group	Mn req (pg/cell)	Group
**a.BG11$_{N2}$**	**5.34 ± 0.62**	**A**	**3.26 ± 0.635**	**A**	**0.06 ± 0.02**	**A**
Fe 0.3 ppm	4.9 ± 0.66	*	2.94 ± 0.72	*	0.05 ± 0.02	*
Fe 1.2 ppm	5.76 ± 0.755	*	3.24 ± 0.935	*	0.04 ± 0.02	*
Fe 5.0 ppm	5.36 ± 0.34	*	3.59 ± 0.44	*	0.11 ± 0	**
**b.BG11$_{NO3}$**	**5.3 ± 0.625**	**A**	**3.16 ± 0.68**	**A**	**0.1 ± 0.06**	**A**
Fe 0.3 ppm	4.98 ± 0.57	*	3.06 ± 0.855	*	0.05 ± 0.03	*
Fe 1.2 ppm	4.43 ± 0.615	*	3.23 ± 0.91	*	0.04 ± 0.02	*
Fe 5.0 ppm	6.5 ± 0.535	**	3.2 ± 0.525	*	0.21 ± 0.05	**
**c.BG11$_{urea}$**	**9.21 ± 1.68**	**B**	**6.43 ± 1.55**	**B**	**0.25 ± 0.14**	**B**
Fe 0.3 ppm	10.91 ± 1.825	*	7.89 ± 1.35	*	0.24 ± 0.21	*
Fe 1.2 ppm	4.17 ± 0.66	**	1.73 ± 0.195	**	0.13 ± 0.05	*
Fe 5.0 ppm	12.56 ± 1.185	*	9.67 ± 1.85	*	0.37 ± 0.15	**

Calibration curves for β-Carotene, urea, and mineral element consumption

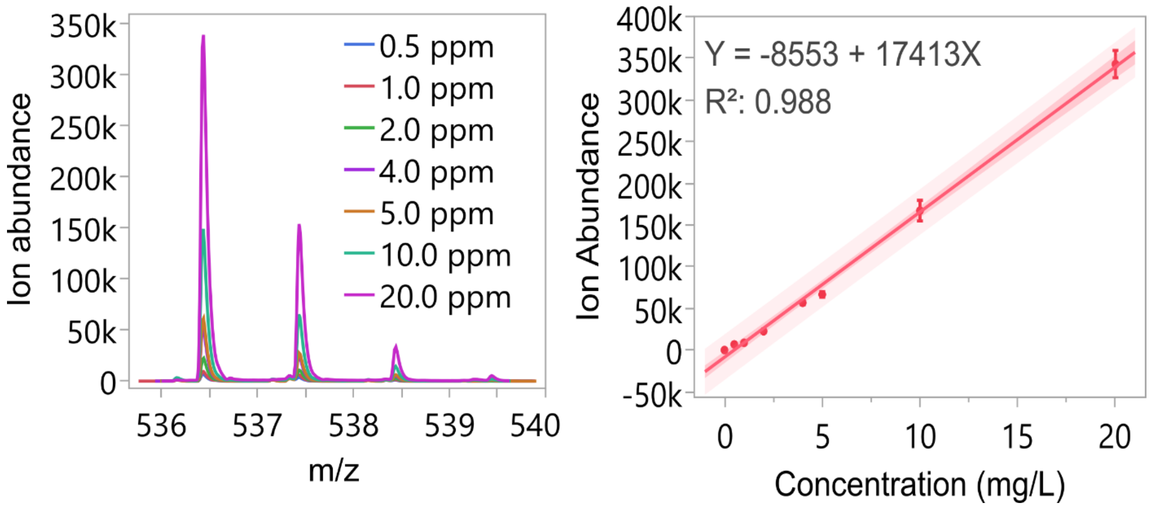

Figure S16. Calibration curve for quantification of β-Carotene

The ion abundance of β-Carotene changes with its concentration in the methanolic extract. A linear calibration curve was determined for the β-Carotene concentration range between 0 and 20 μg ml^{-1}, using the ion abundance signal for the m/z = 536.4382 peak. Markers represent mean values for three independent replicates per calibration point. Shaded area represents the confidence interval of the predicted values, with significance level α=0.05. Error bars were constructed using one standard error from the mean.

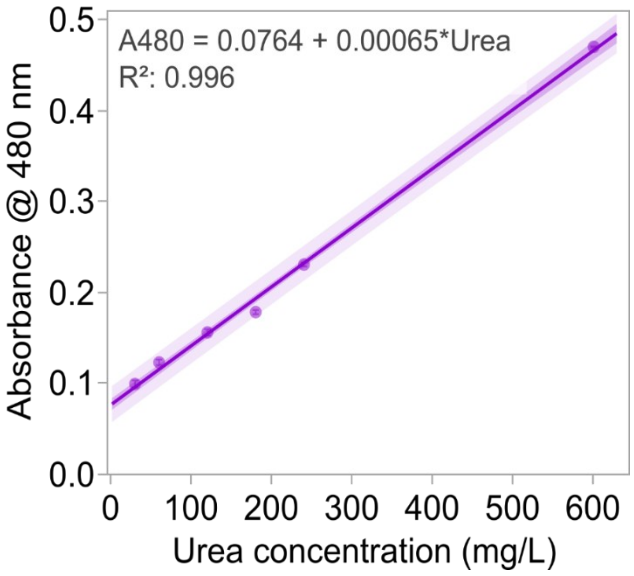

Figure S17. Calibration curve for urea quantification

Urea was determined using a UV-visible spectrophotometry method based on the O-phthalaldehyde method. Eighteen urea standards were employed to build this curve. Markers represent mean values for three independent replicates per calibration point. Shaded area represents the confidence interval of the prediction with a significance level α=0.05.

Analysis of mineral element utilization in *Anabaena* cultures

The main text discuses consumption of P, Ca, Mg, and Mn. Here, the discussion is expanded to consider the remaining micronutrients from BG11 medium. While Fe was almost immediately exhausted, Na, K, Ni, and Co concentration profiles in the growth medium presented nonapparent variations. This can be explained because Na^+ and K^+ are continuously exchanged between the cells and their surroundings for homeostatic reasons [11]. Although Ni is needed for urease activity, the Ni-consumption in $BG11_{NO3}$, $BG11_{N2}$, and $BG11_{urea}$ cultures was insignificant. This is explained because Ni-requirements in urea-consuming cyanobacteria are extremely low [28,29]. Urease activity was mainly affected by the presence of urea and Fe-availability. While Co is necessary for the biosynthesis of Vitamin B12 (Cobalamin) in cyanobacteria, the requirements of these element are also extremely low [44].

The remaining elements presented in the ranking of Figure 6 are B, Mo, Zn, and Cu. Although cell requirements of these elements are low, these micronutrients have essential functions in *Anabaena*. B is utilized for bacterial quorum sensing, a process that confers communal response during changing environmental conditions [45]. This element is also involved in heterocyst stabilization and Nitrogenase activity [46]. Mo is incorporated in molybdoenzymes involved in N assimilation processes. Therefore, a basal consumption of this element is necessary for metabolizing N_2, nitrates and urea in photosynthetic organisms [47,48]. Since Mo demands are higher for Nitrogenase activity, increased Mo consumption is expected for N_2-fixing organisms [49]. While Zn participates in DNA protection, gene regulation and carbonic anhydrase activation, Cu participates electron transport through photosystem I as Plastocyanin [32,33,50]. Interestingly, the Zn requirements per cell were lower with increasing Fe-levels in all growth media. This effect can be associated with Fe and Zn homeostasis processes involving the Zn uptake regulator (*Zur*), which is essential for cellular defense during oxidative stress conditions [33].

The relative importance of each of these elements may be related to their abundance in standard BG11 [51]. However, the element demands presented here are not necessarily proportional to the concentrations of these minerals in the growth media.

Calibration curves of the ICP method used for mineral element quantification

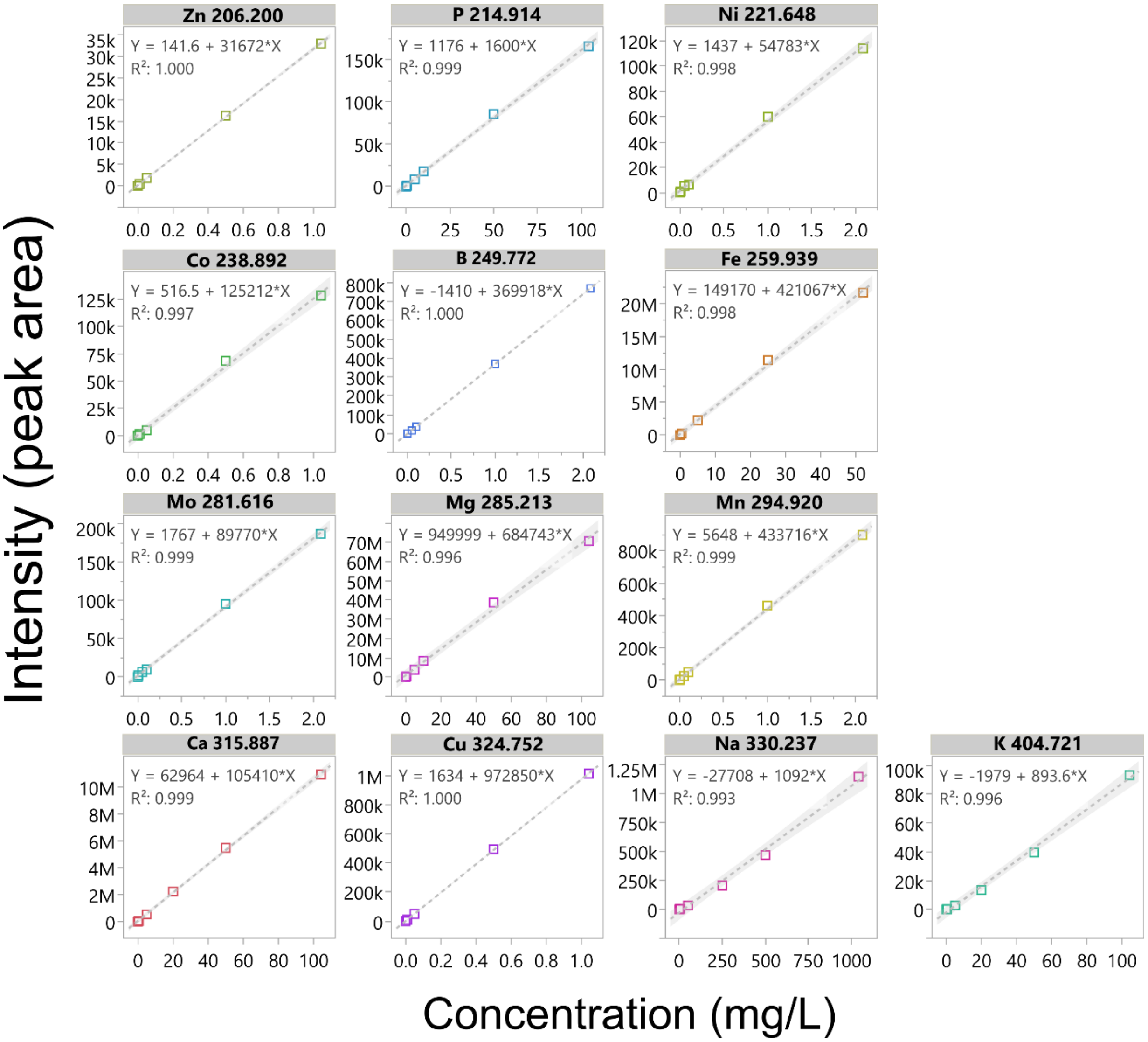

Figure S18. ICP-OES Calibration for elemental analysis of BG11 media

Simultaneous quantification of Zn, P, Ni, Co, B, Fe, Mo, Mg, Mn, Ca, Cu, Na, and K levels in cyanobacterial media was conducted using an atomic emission technique. The calibration curves and detection wavelengths (in nm) for each element are presented. Markers represent mean values for two independent replicates per calibration point. Shaded regions represent the confidence interval of the predictions with a significance level α=0.05.

Table S8. Regression equations for mineral consumption

Each regression was obtained from N=16 pairs per medium type and Fe-level combination for each element consumed. Errors are estimated from confidence fit intervals with significance level α=0.05.

Element	Medium	Fe level (ppm)	Nutrient consumption expression	R^2
P (μg/mL)	$BG11_{NO3}$	0.3	$(0.66 \pm 0.19) + (1.71 \times 10^{-3} \pm 1.16 \times 10^{-4}) * \sqrt{CD}$	0.94
		1.2	$(0.78 \pm 0.42) + (1.94 \times 10^{-3} \pm 2.51 \times 10^{-4}) * \sqrt{CD}$	0.81
		5.0	$(1.81 \pm 0.51) + (2.28 \times 10^{-3} \pm 3.02 \times 10^{-4}) * \sqrt{CD}$	0.80
	$BG11_{N2}$	0.3	$(0.43 \pm 0.36) + (2.42 \times 10^{-3} \pm 3.87 \times 10^{-4}) * \sqrt{CD}$	0.74
		1.2	$(0.60 \pm 0.43) + (3.19 \times 10^{-3} \pm 4.48 \times 10^{-4}) * \sqrt{CD}$	0.78
		5.0	$(1.67 \pm 0.69) + (3.77 \times 10^{-3} \pm 6.42 \times 10^{-4}) * \sqrt{CD}$	0.71
	$BG11_{urea}$	0.3	$(0.65 \pm 0.36) + (1.35 \times 10^{-6} \pm 3.12 \times 10^{-7}) * CD$	0.57
		1.2	$(-0.23 \pm 0.25) + (2.42 \times 10^{-3} \pm 2.21 \times 10^{-4}) * \sqrt{CD}$	0.92
		5.0	$(0.79 \pm 0.36) + (2.99 \times 10^{-3} \pm 3.26 \times 10^{-4}) * \sqrt{CD}$	0.86
Ca (μg/mL)	$BG11_{NO3}$	0.3	$(0.07 \pm 0.10) + (9.77 \times 10^{-4} \pm 6.45 \times 10^{-5}) * \sqrt{CD}$	0.94
		1.2	$(0.18 \pm 0.13) + (1.13 \times 10^{-3} \pm 7.85 \times 10^{-5}) * \sqrt{CD}$	0.94
		5.0	$(0.40 \pm 0.17) + (1.29 \times 10^{-3} \pm 1.02 \times 10^{-4}) * \sqrt{CD}$	0.92
	$BG11_{N2}$	0.3	$(0.02 \pm 0.43) + (1.57 \times 10^{-3} \pm 4.66 \times 10^{-4}) * \sqrt{CD}$	0.45
		1.2	$(-0.11 \pm 0.33) + (1.95 \times 10^{-3} \pm 3.45 \times 10^{-4}) * \sqrt{CD}$	0.70
		5.0	$(0.99 \pm 0.53) + (2.26 \times 10^{-3} \pm 4.91 \times 10^{-4}) * \sqrt{CD}$	0.60
	$BG11_{urea}$	0.3	$(0.36 \pm 0.27) + (1.76 \times 10^{-3} \pm 3.05 \times 10^{-4}) * \sqrt{CD}$	0.71
		1.2	$(0.08 \pm 0.08) + (6.55 \times 10^{-4} \pm 7.25 \times 10^{-5}) * \sqrt{CD}$	0.88
		5.0	$(1.33 \pm 0.42) + (9.18 \times 10^{-4} \pm 3.80 \times 10^{-4}) * \sqrt{CD}$	0.29
Mg (μg/mL)	$BG11_{NO3}$	0.3	$(0.12 \pm 0.05) + (2.27 \times 10^{-7} \pm 1.22 \times 10^{-8}) * CD$	0.96
		1.2	$(-0.04 \pm 0.33) + (7.24 \times 10^{-4} \pm 1.98 \times 10^{-4}) * \sqrt{CD}$	0.49
		5.0	$(0.003 \pm 0.18) + (7.24 \times 10^{-4} \pm 1.98 \times 10^{-4}) * \sqrt{CD}$	0.83
	$BG11_{N2}$	0.3	$(0.05 \pm 0.13) + (6.08 \times 10^{-7} \pm 1.13 \times 10^{-7}) * CD$	0.62
		1.2	$(-0.25 \pm 0.22) + (1.06 \times 10^{-3} \pm 2.27 \times 10^{-4}) * \sqrt{CD}$	0.61
		5.0	$(-0.09 \pm 0.29) + (1.05 \times 10^{-3} \pm 2.67 \times 10^{-4}) * \sqrt{CD}$	0.53
	$BG11_{urea}$	0.3	$(0.18 \pm 0.14) + (5.41 \times 10^{-4} \pm 1.52 \times 10^{-4}) * \sqrt{CD}$	0.47
		1.2	$(0.05 \pm 0.07) + (5.21 \times 10^{-4} \pm 6.03 \times 10^{-5}) * \sqrt{CD}$	0.87
		5.0	$(0.10 \pm 0.06) + (4.34 \times 10^{-4} \pm 5.02 \times 10^{-5}) * \sqrt{CD}$	0.84
Mn (μg/mL)	$BG11_{NO3}$	0.3	$(-0.012 \pm 0.008) + (3.08 \times 10^{-5} \pm 5.14 \times 10^{-6}) * \sqrt{CD}$	0.72
		1.2	$(-0.005 \pm 0.010) + (3.18 \times 10^{-5} \pm 6.27 \times 10^{-6}) * \sqrt{CD}$	0.65
		5.0	$(-0.005 \pm 0.010) + (3.18 \times 10^{-5} \pm 6.27 \times 10^{-6}) * \sqrt{CD}$	0.78
	$BG11_{N2}$	0.3	$(0.006 \pm 0.011) + (3.52 \times 10^{-8} \pm 9.57 \times 10^{-9}) * CD$	0.49
		1.2	$(0.002 \pm 0.011) + (4.47 \times 10^{-8} \pm 9.09 \times 10^{-9}) * CD$	0.63
		5.0	$(0.029 \pm 0.013) + (9.72 \times 10^{-5} \pm 1.20 \times 10^{-5}) * \sqrt{CD}$	0.82
	$BG11_{urea}$	0.3	$(0.015 \pm 0.010) + (3.39 \times 10^{-5} \pm 1.16 \times 10^{-5}) * \sqrt{CD}$	0.38
		1.2	$(0.007 \pm 0.006) + (3.66 \times 10^{-5} \pm 5.04 \times 10^{-6}) * \sqrt{CD}$	0.83
		5.0	$(0.025 \pm 0.008) + (5.42 \times 10^{-5} \pm 7.28 \times 10^{-6}) * \sqrt{CD}$	0.80
B (μg/mL)	$BG11_{NO3}$	0.3	$(0.006 \pm 0.004) + (2.59 \times 10^{-5} \pm 2.53 \times 10^{-6}) * \sqrt{CD}$	0.88
		1.2	$(0.003 \pm 0.007) + (3.16 \times 10^{-5} \pm 4.14 \times 10^{-6}) * \sqrt{CD}$	0.80
		5.0	$(0.003 \pm 0.01) + (2.82 \times 10^{-5} \pm 5.54 \times 10^{-6}) * \sqrt{CD}$	0.65
	$BG11_{N2}$	0.3	$(-0.001 \pm 0.01) + (6.17 \times 10^{-5} \pm 1.47 \times 10^{-5}) * \sqrt{CD}$	0.56
		1.2	$(-0.007 \pm 0.02) + (6.20 \times 10^{-5} \pm 1.59 \times 10^{-5}) * \sqrt{CD}$	0.52
		5.0	$(-0.005 \pm 0.02) + (3.97 \times 10^{-5} \pm 1.42 \times 10^{-5}) * \sqrt{CD}$	0.36
	$BG11_{urea}$	0.3	$(0.01 \pm 0.01) + (4.98 \times 10^{-5} \pm 1.26 \times 10^{-5}) * \sqrt{CD}$	0.53
		1.2	$(0.006 \pm 0.009) + (4.89 \times 10^{-5} \pm 7.82 \times 10^{-6}) * \sqrt{CD}$	0.78
		5.0	$(0.01 \pm 0.008) + (3.32 \times 10^{-5} \pm 7.06 \times 10^{-6}) * \sqrt{CD}$	0.61

Table S8. Continued

Element	Medium	Fe level (ppm)	Nutrient consumption expression	R^2
Mo (µg/mL)	$BG11_{NO3}$	0.3	$(0.006 \pm 0.002) + (1.16 \times 10^{-5} \pm 1.14 \times 10^{-6}) * \sqrt{CD}$	0.88
		1.2	$(0.0003 \pm 0.003) + (1.80 \times 10^{-5} \pm 1.94 \times 10^{-6}) * \sqrt{CD}$	0.86
		5.0	$(-0.002 \pm 0.002) + (1.77 \times 10^{-5} \pm 1.10 \times 10^{-6}) * \sqrt{CD}$	0.95
	$BG11_{N2}$	0.3	$(0.011 \pm 0.009) + (4.36 \times 10^{-5} \pm 9.45 \times 10^{-6}) * \sqrt{CD}$	0.60
		1.2	$(0.007 \pm 0.004) + (2.75 \times 10^{-5} \pm 4.05 \times 10^{-6}) * \sqrt{CD}$	0.77
		5.0	$(0.018 \pm 0.018) + (5.05 \times 10^{-5} \pm 1.68 \times 10^{-5}) * \sqrt{CD}$	0.39
	$BG11_{urea}$	0.3	$(0.005 \pm 0.002) + (2.38 \times 10^{-5} \pm 2.13 \times 10^{-6}) * \sqrt{CD}$	0.90
		1.2	$(0.001 \pm 0.002) + (1.72 \times 10^{-5} \pm 1.65 \times 10^{-6}) * \sqrt{CD}$	0.91
		5.0	$(0.004 \pm 0.004) + (1.94 \times 10^{-5} \pm 3.58 \times 10^{-6}) * \sqrt{CD}$	0.68
Zn (µg/mL)	$BG11_{NO3}$	0.3	$(0.004 \pm 0.001) + (8.50 \times 10^{-6} \pm 9.21 \times 10^{-7}) * \sqrt{CD}$	0.86
		1.2	$(-0.001 \pm 0.002) + (7.75 \times 10^{-6} \pm 1.19 \times 10^{-6}) * \sqrt{CD}$	0.75
		5.0	$(0.018 \pm 0.006) + (6.56 \times 10^{-6} \pm 3.82 \times 10^{-6}) * \sqrt{CD}$	0.17
	$BG11_{N2}$	0.3	$(0.002 \pm 0.003) + (1.36 \times 10^{-5} \pm 3.24 \times 10^{-6}) * \sqrt{CD}$	0.56
		1.2	$(0.002 \pm 0.005) + (1.95 \times 10^{-5} \pm 5.31 \times 10^{-6}) * \sqrt{CD}$	0.49
		5.0	$(-0.001 \pm 0.003) + (8.99 \times 10^{-6} \pm 2.38 \times 10^{-6}) * \sqrt{CD}$	0.50
	$BG11_{urea}$	0.3	$(0.005 \pm 0.002) + (7.94 \times 10^{-6} \pm 2.28 \times 10^{-6}) * \sqrt{CD}$	0.46
		1.2	$(0.005 \pm 0.002) + (6.67 \times 10^{-6} \pm 1.67 \times 10^{-6}) * \sqrt{CD}$	0.59
		5.0	$(0.001 \pm 0.001) + (5.63 \times 10^{-6} \pm 7.76 \times 10^{-7}) * \sqrt{CD}$	0.79
Cu (µg/mL)	$BG11_{NO3}$	0.3	$(0.003 \pm 0.001) + (4.25 \times 10^{-6} \pm 6.49 \times 10^{-7}) * \sqrt{CD}$	0.75
		1.2	$(0.001 \pm 0.001) + (4.50 \times 10^{-6} \pm 5.06 \times 10^{-7}) * \sqrt{CD}$	0.85
		5.0	$(0.001 \pm 0.001) + (4.29 \times 10^{-6} \pm 4.23 \times 10^{-7}) * \sqrt{CD}$	0.88
	$BG11_{N2}$	0.3	$(0.00 \pm 0.00) + (5.42 \times 10^{-6} \pm 5.61 \times 10^{-7}) * \sqrt{CD}$	0.87
		1.2	$(0.0001 \pm 0.000) + (3.44 \times 10^{-9} \pm 5.33 \times 10^{-10}) * CD$	0.75
		5.0	$(0.00 \pm 0.001) + (6.85 \times 10^{-6} \pm 1.06 \times 10^{-7}) * \sqrt{CD}$	0.75
	$BG11_{urea}$	0.3	$(0.002 \pm 0.001) + (3.52 \times 10^{-6} \pm 7.70 \times 10^{-7}) * \sqrt{CD}$	0.60
		1.2	$(0.001 \pm 0.001) + (4.14 \times 10^{-6} \pm 5.06 \times 10^{-7}) * \sqrt{CD}$	0.86
		5.0	$(0.000 \pm 0.001) + (4.45 \times 10^{-6} \pm 5.01 \times 10^{-7}) * \sqrt{CD}$	0.85

CD: Cellular density produced (cells/mL)

Table S9. Element concentrations of calibrations standards for ICP-OES.

These concentration levels were defined considering typical element levels in standard BG11 medium. Standards were prepared in HNO_3 2% v/v from commercial standards for ICP-OES.

**Element**	**ST1**	**ST2**	**ST3**	**ST4**	**ST5**
Na (mg/L)	5	50	250	500	1042
K (mg/L)	0.5	5	20	50	104.2
Ca (mg/L)	0.5	5	20	50	104.2
Mg (mg/L)	0.5	5	10	50	104.2
Fe (mg/L)	0.05	0.5	5	25	52.1
P (mg/L)	0.5	5	10	50	104.2
B (mg/L)	0.005	0.05	0.1	1	2.084
Ni (mg/L)	0.005	0.05	0.1	1	2.084
Mo (mg/L)	0.005	0.05	0.1	1	2.084
Cu (mg/L)	0.001	0.01	0.05	0.5	1.042
Co (mg/L)	0.001	0.01	0.05	0.5	1.042
Mn (mg/L)	0.005	0.05	0.1	1	2.084
Zn (mg/L)	0.001	0.01	0.05	0.5	1.042

Table S10. N_2-fixation rates of diazotrophic cyanobacteria

	**Mean or median N_2-fixation rate**			
Cyanobacteria	fmol-N cell $^{-1}$ h^{-1}	nmol-N_2 (µg Chl *a*)$^{-1}$ h^{-1}	mg-N L^{-1} day $^{-1}$	Reference
Anabaena sp. UTEX 2576*	3.8	0.92	1.55	This study
Aphanizomenon sp.	5	-	-	[52]
Dolichospermum spp.	10	-	-	[52]
Nodularia spumigena	20	-	-	[52]
Aphanizomenon sp.	2.1	-	-	[53]
Nodularia spumigena	13	-	-	[52]
Nostoc sp. from *Macrozamia riedlei*	-	0.3	-	[54]
Nostoc muscorum	-	-	2.17	[55]
Filamentous heterocystous	3.5	-	-	[52]
Trichodesmium sp.	2	-	-	[52]
Unicellular cyanobacteria	2.5	-	-	[52]
Symbiotic cyanobacteria	0.8	-	-	[52]

*Values are representative for exponential-phase $BG11_{N2}$ cultures on day 4. Different units are provided to easily compare reported N_2-fixation rates from different studies

Supplementary References

1. Adolph, K.W.; Haselkorn, R. Isolation and Characterization of a Virus Infecting the Blue-Green Alga Nostoc Muscorum. *Virology* **1971**, *46*, 200–208, doi:https://doi.org/10.1016/0042-6822(71)90023-7.
2. Rippka, R.; Deruelles, J.; Waterbury, J.B.; Herdman, M.; Stanier, R.Y. Generic Assignments, Strain Histories and Properties of Pure Cultures of Cyanobacteria. *Microbiology* **1979**, *111*, 1–61, doi:10.1099/00221287-111-1-1.
3. *Algal Culturing Techniques*; Andersen, R.A., Ed.; Elsevier/Academic Press: Burlington, Mass, 2005; ISBN 978-0-12-088426-1.
4. Allen, M.M.; Stanier, R.Y. Growth and Division of Some Unicellular Blue-Green Algae. *Journal of General Microbiology* **1968**, *51*, 199–202, doi:10.1099/00221287-51-2-199.
5. Thomas, D.L.; Mantes, J.G. Spectrophotometrically Assayed Inhibitory Effects of Mercuric Compounds on Anabaena Flos-Aquae and Anacystis Nidulans (Cyanophyceae)1. *Journal of Phycology* **1978**, *14*, 494–499, doi:https://doi.org/10.1111/j.1529-8817.1978.tb02475.x.
6. Cordeiro, R.S.; Vaz, I.C.D.; Magalhães, S.M.S.; Barbosa, F.A.R. Effects of Nutritional Conditions on Lipid Production by Cyanobacteria. *An. Acad. Bras. Ciênc.* **2017**, *89*, 2021–2031, doi:10.1590/0001-3765201720150707.
7. George, B.; Pancha, I.; Desai, C.; Chokshi, K.; Paliwal, C.; Ghosh, T.; Mishra, S. Effects of Different Media Composition, Light Intensity and Photoperiod on Morphology and Physiology of Freshwater Microalgae Ankistrodesmus Falcatus – A Potential Strain for Bio-Fuel Production. *Bioresource Technology* **2014**, *171*, 367–374, doi:10.1016/j.biortech.2014.08.086.
8. Atkinson, M.J.; work(s):, S.V.S.R. C:N:P Ratios of Benthic Marine Plants. *Limnology and Oceanography* **1983**, *28*, 568–574.
9. Madkour, F.F.; Kamil, A.E.-W.; Nasr, H.S. Production and Nutritive Value of Spirulina Platensis in Reduced Cost Media. *The Egyptian Journal of Aquatic Research* **2012**, *38*, 51–57, doi:10.1016/j.ejar.2012.09.003.
10. Noreña-Caro, D.; Benton, M.G. Cyanobacteria as Photoautotrophic Biofactories of High-Value Chemicals. *Journal of CO2 Utilization* **2018**, *Manuscript submitted for publication*.
11. Flores, E.; Herrero, A. *Cell Biology of Cyanobacteria*; Caister Academic Press, 2014; ISBN 978-1-908230-92-8.
12. Takenaka, H.; Yamaguchi, Y. Commercial-scale culturing of cyanobacteria: an industrial experience. In *Cyanobacteria*; Sharma, N.K., Rai, A.K., Stal, L.J., Eds.; John Wiley & Sons, Ltd: Chichester, UK, 2013; pp. 293–301 ISBN 978-1-118-40223-8.
13. Bermejo, R. Phycocyanins. In *Cyanobacteria*; Sharma, N.K., Rai, A.K., Stal, L.J., Eds.; John Wiley & Sons, Ltd, 2014; pp. 209–225 ISBN 978-1-118-40223-8.
14. Jacob-Lopes, E.; Gimenes Scoparo, C.H.; Queiroz, M.I.; Franco, T.T. Biotransformations of Carbon Dioxide in Photobioreactors. *Energy Conversion and Management* **2010**, *51*, 894–900, doi:10.1016/j.enconman.2009.11.027.
15. Zhang, K.; Miyachi, S.; Kurano, N. Evaluation of a Vertical Flat-Plate Photobioreactor for Outdoor Biomass Production and Carbon Dioxide Bio-Fixation: Effects of Reactor Dimensions, Irradiation and Cell Concentration on the Biomass Productivity and Irradiation Utilization Efficiency. *Appl. Microbiol. Biotechnol.* **2001**, *55*, 428–433.
16. Martínez, L.; Redondas, V.; García, A.-I.; Morán, A. Optimization of Growth Operational Conditions for CO2 Biofixation by Native Synechocystis Sp. *Journal of Chemical Technology & Biotechnology* **2011**, *86*, 681–690, doi:10.1002/jctb.2568.
17. González López, C.V.; Acién Fernández, F.G.; Fernández Sevilla, J.M.; Sánchez Fernández, J.F.; Cerón García, M.C.; Molina Grima, E. Utilization of the Cyanobacteria Anabaena Sp. ATCC 33047 in CO2 Removal Processes. *Bioresource Technology* **2009**, *100*, 5904–5910, doi:10.1016/j.biortech.2009.04.070.
18. Kumar, A.; Yuan, X.; Sahu, A.K.; Dewulf, J.; Ergas, S.J.; Langenhove, H.V. A Hollow Fiber Membrane Photo-Bioreactor for CO2 Sequestration from Combustion Gas Coupled with Wastewater Treatment: A Process Engineering Approach. *Journal of Chemical Technology & Biotechnology* **2010**, *85*, 387–394, doi:10.1002/jctb.2332.
19. Chiang, C.-L.; Lee, C.-M.; Chen, P.-C. Utilization of the Cyanobacteria Anabaena Sp. CH1 in Biological Carbon Dioxide Mitigation Processes. *Bioresource Technology* **2011**, *102*, 5400–5405, doi:10.1016/j.biortech.2010.10.089.

20. Nayak, B.K.; Das, D. Improvement of Carbon Dioxide Biofixation in a Photobioreactor Using Anabaena Sp. PCC 7120. *Process Biochemistry* **2013**, *48*, 1126–1132, doi:10.1016/j.procbio.2013.05.015.
21. Kajiwara, S.; Yamada, H.; Ohkuni, N.; Ohtaguchi, K. Design of the Bioreactor for Carbon Dioxide Fixation by Synechococcus PCC7942. *Energy Conversion and Management* **1997**, *38*, S529–S532, doi:10.1016/S0196-8904(96)00322-6.
22. Jin, H.-F.; Lim, B.-R.; Lee, K. Influence of Nitrate Feeding on Carbon Dioxide Fixation by Microalgae. *Journal of Environmental Science and Health, Part A* **2006**, *41*, 2813–2824, doi:10.1080/10934520600967928.
23. Sydney, E.B.; Sturm, W.; de Carvalho, J.C.; Thomaz-Soccol, V.; Larroche, C.; Pandey, A.; Soccol, C.R. Potential Carbon Dioxide Fixation by Industrially Important Microalgae. *Bioresource Technology* **2010**, *101*, 5892–5896, doi:10.1016/j.biortech.2010.02.088.
24. Gonçalves, A.L.; Rodrigues, C.M.; Pires, J.C.M.; Simões, M. The Effect of Increasing CO 2 Concentrations on Its Capture, Biomass Production and Wastewater Bioremediation by Microalgae and Cyanobacteria. *Algal Research* **2016**, *14*, 127–136, doi:10.1016/j.algal.2016.01.008.
25. Fischer, E.R.; Hansen, B.T.; Nair, V.; Hoyt, F.H.; Dorward, D.W. Scanning Electron Microscopy. *Curr Protoc Microbiol* **2012**, *CHAPTER*, Unit2B.2, doi:10.1002/9780471729259.mc02b02s25.
26. Liang, F.T.; Xu, Q.; Sikdar, R.; Xiao, Y.; Cox, J.S.; Doerrler, W.T. BB0250 of Borrelia Burgdorferi Is a Conserved and Essential Inner Membrane Protein Required for Cell Division. *J. Bacteriol.* **2010**, *192*, 6105–6115, doi:10.1128/JB.00571-10.
27. Herrero, A.; Muro-Pastor, A.M.; Valladares, A.; Flores, E. Cellular Differentiation and the NtcA Transcription Factor in Filamentous Cyanobacteria. *FEMS Microbiology Reviews* **2004**, *28*, 469–487, doi:10.1016/j.femsre.2004.04.003.
28. Sakamoto, T.; Delgaizo, V.B.; Bryant, D.A. Growth on Urea Can Trigger Death and Peroxidation of the Cyanobacterium Synechococcus Sp. Strain PCC 7002. *APPL. ENVIRON. MICROBIOL.* **1998**, *64*, 6.
29. Sakamoto, T.; Bryant, D.A. Requirement of Nickel as an Essential Micronutrient for the Utilization of Urea in the Marine Cyanobacterium Synechococcus Sp. PCC 7002. *Microbes and Environments* **2001**, *16*, 177–184, doi:10.1264/jsme2.2001.177.
30. Krajewska, B. Ureases I. Functional, Catalytic and Kinetic Properties: A Review. *Journal of Molecular Catalysis B: Enzymatic* **2009**, *59*, 9–21, doi:10.1016/j.molcatb.2009.01.003.
31. Veaudor, T.; Cassier-Chauvat, C.; Chauvat, F. Genomics of Urea Transport and Catabolism in Cyanobacteria: Biotechnological Implications. *Front. Microbiol.* **2019**, *10*, 2052, doi:10.3389/fmicb.2019.02052.
32. Blindauer, C.A. Zinc-Handling in Cyanobacteria: An Update. *Chemistry & Biodiversity* **2008**, *5*, 1990–2013, doi:10.1002/cbdv.200890183.
33. Sein-Echaluce, V.C.; González, A.; Napolitano, M.; Luque, I.; Barja, F.; Peleato, M.L.; Fillat, M.F. Zur (FurB) Is a Key Factor in the Control of the Oxidative Stress Response in Anabaena Sp. PCC 7120. *Environmental Microbiology* **2015**, *17*, 2006–2017, doi:10.1111/1462-2920.12628.
34. Meeks, J.C.; Elhai, J. Regulation of Cellular Differentiation in Filamentous Cyanobacteria in Free-Living and Plant-Associated Symbiotic Growth States. *Microbiol. Mol. Biol. Rev.* **2002**, *66*, 94–121, doi:10.1128/MMBR.66.1.94-121.2002.
35. Canini, A.; Grilli Caiola, M.; Mascini, M. Ammonium Content, Nitrogenase Activity and Heterocyst Frequency within the Leaf Cavities of Azolla Filiculoides Lam. *FEMS Microbiology Letters* **1990**, *71*, 205–210, doi:10.1111/j.1574-6968.1990.tb03823.x.
36. Kerby, N.W.; Rowell, P.; Stewart, W.D.P. Cyanobacterial Ammonium Transport, Ammonium Assimilation, and Nitrogenase Regulation. *New Zealand Journal of Marine and Freshwater Research* **1987**, *21*, 447–455, doi:10.1080/00288330.1987.9516240.
37. Yoch, D.C.; Gotto, J.W. Effect of Light Intensity and Inhibitors of Nitrogen Assimilation on NH4+ Inhibition of Nitrogenase Activity in Rhodospirillum Rubrum and Anabaena Sp. *Journal of Bacteriology* **1982**, *151*, 800–806, doi:10.1128/JB.151.2.800-806.1982.
38. Takaichi, S.; Mochimaru, M.; Maoka, T.; Katoh, H. Myxol and 4-Ketomyxol 2′-Fucosides, Not Rhamnosides, from Anabaena Sp. PCC 7120 and Nostoc Punctiforme PCC 73102, and Proposal for the Biosynthetic Pathway of Carotenoids. *Plant Cell Physiol* **2005**, *46*, 497–504, doi:10.1093/pcp/pci049.
39. Takaichi, S.; Maoka, T.; Mochimaru, M. Unique Carotenoids in the Terrestrial Cyanobacterium Nostoc Commune NIES-24: 2-Hydroxymyxol 2′-Fucoside, Nostoxanthin and Canthaxanthin. *Current Microbiology* **2009**, *59*, 413–419, doi:10.1007/s00284-009-9453-4.
40. Amorim-Carrilho, K.T.; Cepeda, A.; Fente, C.; Regal, P. Review of Methods for Analysis of Carotenoids. *TrAC Trends in Analytical Chemistry* **2014**, *56*, 49–73, doi:10.1016/j.trac.2013.12.011.

41. HACH NitraVer® 5 Nitrate Reagent Powder Pillows, 10 ML, Pk/100 | Hach USA - Overview Available online: https://www.hach.com/nitraver-5-nitrate-reagent-powder-pillows-10-ml-pk-100/product?id=7640208933 (accessed on 7 February 2021).
42. Jung, D.; Biggs, H.; Erikson, J.; Ledyard, P.U. New Colorimetric Reaction for End-Point, Continuous-Flow, and Kinetic Measurement of Urea. *Clinical Chemistry* **1975**, *21*, 1136–1140, doi:10.1093/clinchem/21.8.1136.
43. HACH Total Nitrogen Reagent Set, HR, TNT | Hach USA - Overview Available online: https://www.hach.com/total-nitrogen-reagent-set-hr-tnt/product?id=7640209860 (accessed on 25 July 2019).
44. Helliwell, K.E.; Lawrence, A.D.; Holzer, A.; Kudahl, U.J.; Sasso, S.; Kräutler, B.; Scanlan, D.J.; Warren, M.J.; Smith, A.G. Cyanobacteria and Eukaryotic Algae Use Different Chemical Variants of Vitamin B12. *Curr Biol* **2016**, *26*, 999–1008, doi:10.1016/j.cub.2016.02.041.
45. Breydo, L. Boron, Biologically Active Compounds. In *Encyclopedia of Metalloproteins*; Kretsinger, R.H., Uversky, V.N., Permyakov, E.A., Eds.; Springer: New York, NY, 2013; pp. 295–299 ISBN 978-1-4614-1533-6.
46. Bonilla, I.; Garcia-González, M.; Mateo, P. Boron Requirement in Cyanobacteria: Its Possible Role in the Early Evolution of Photosynthetic Organisms. *Plant Physiology* **1990**, *94*, 1554–1560, doi:10.1104/pp.94.4.1554.
47. Moraes, M.F.; Reis, A.R.; Moraes, L.A.C.; Lavres-Junior, J.; Vivian, R.; Cabral, C.P.; Malavolta, E. Effects of Molybdenum, Nickel, and Nitrogen Sources on the Mineral Nutrition and Growth of Rice Plants. *Communications in Soil Science and Plant Analysis* **2009**, *40*, 3238–3251, doi:10.1080/00103620903267590.
48. Cole, J.J.; Lane, J.M.; Marino, R.; Howarth, R.W. Molybdenum Assimilation by Cyanobacteria and Phytoplankton in Freshwater and Salt Water. *Limnology and Oceanography* **1993**, *38*, 25–35, doi:10.4319/lo.1993.38.1.0025.
49. Glass, J.B.; Wolfe-Simon, F.; Anbar, A.D. Coevolution of Metal Availability and Nitrogen Assimilation in Cyanobacteria and Algae. *Geobiology* **2009**, *7*, 100–123, doi:10.1111/j.1472-4669.2009.00190.x.
50. Jordan, P.; Fromme, P.; Witt, H.T.; Klukas, O.; Saenger, W.; Krauß, N. Three-Dimensional Structure of Cyanobacterial Photosystem I at 2.5 Å Resolution. *Nature* **2001**, *411*, 909–917, doi:10.1038/35082000.
51. Rippka, R.; Deruelles, J.; Waterbury, J.B.; Herdman, M.; Stanier, R.Y. Generic Assignments, Strain Histories and Properties of Pure Cultures of Cyanobacteria. *Microbiology* **1979**, *111*, 1–61, doi:10.1099/00221287-111-1-1.
52. Klawonn, I.; Nahar, N.; Walve, J.; Andersson, B.; Olofsson, M.; Svedén, J.B.; Littmann, S.; Whitehouse, M.J.; Kuypers, M.M.M.; Ploug, H. Cell-Specific Nitrogen- and Carbon-Fixation of Cyanobacteria in a Temperate Marine System (Baltic Sea). *Environmental Microbiology* **2016**, *18*, 4596–4609, doi:https://doi.org/10.1111/1462-2920.13557.
53. Svedén, J.B.; Adam, B.; Walve, J.; Nahar, N.; Musat, N.; Lavik, G.; Whitehouse, M.J.; Kuypers, M.M.M.; Ploug, H. High Cell-Specific Rates of Nitrogen and Carbon Fixation by the Cyanobacterium Aphanizomenon Sp. at Low Temperatures in the Baltic Sea. *FEMS Microbiology Ecology* **2015**, *91*, doi:10.1093/femsec/fiv131.
54. Lindblad, P.; Atkins, C.A.; Pate, J.S. N2-Fixation by Freshly Isolated Nostoc from Coralloid Roots of the Cycad Macrozamia Riedlei (Fisch. Ex Gaud.) Gardn. *Plant Physiology* **1991**, *95*, 753–759, doi:10.1104/pp.95.3.753.
55. Allison, F.E.; Hoover, S.R.; Morris, H.J. Physiological Studies with the Nitrogen-Fixing Alga, Nostoc Muscorum. *Botanical Gazette* **2015**, doi:10.1086/334654.
